# De Novo-Designed Bifunctional Proteins for Targeted Protein Degradation

**DOI:** 10.1021/jacs.6c07593

**Published:** 2026-08-05

**Authors:** Bram Mylemans, Boguslawa Korona, Amanda M. Acevedo-Jake, Ailsa MacRae, Thomas A. Edwards, Danny T. Huang, Andrew J. Wilson, Laura S. Itzhaki, Derek N. Woolfson

**Affiliations:** † School of Chemistry, 1980 University of Bristol, Cantock’s Close, Bristol BS8 1TS, U.K.; ‡ Novo Nordisk Foundation Center for Protein Design, Department of Biology and Department of Drug Design and Pharmacology, University of Copenhagen, Jagtvej 160, Copenhagen 2100, Denmark; § Department of Pharmacology, 2152 University of Cambridge, Cambridge CB2 1PD, U.K.; ∥ School of Chemistry, 1724 University of Birmingham, Edgbaston B15 2TT, U.K.; ⊥ College of Biomedical Sciences, Larkin University, 18301 N Miami Ave #1, Miami, Florida 33169, U.S.A.; # Cancer Research UK Scotland Institute, Garscube Estate, Switchback Road, Glasgow G61 1BD, U.K.; ∇ School of Cancer Sciences, University of Glasgow, Garscube Estate, Switchback Road, Glasgow G61 1QH, U.K.; ○ School of Chemistry, University of Leeds, Woodhouse Lane, Leeds LS2 9JT, U.K.; ◆ Astbury Centre for Structural Molecular Biology, University of Leeds, Woodhouse Lane, Leeds LS2 9JT, U.K.; ¶ School of Biochemistry, University of Bristol, Medical Sciences Building, University Walk, Bristol BS8 1TD, U.K.; ▼ Max Planck-Bristol Centre for Minimal Biology, University of Bristol, Cantock’s Close, Bristol BS8 1TS, U.K.

## Abstract

In targeted protein degradation (TPD), specific subcellular proteins are removed by routing them to the ubiquitin-proteasome, autophagy, or lysosome machinery. For instance, proteolysis-targeting chimeras (PROTACs) are synthetic heterobifunctional small molecules that simultaneously bind the target and an E3 ubiquitin ligase to drive ubiquitination and degradation by the proteasome. Despite considerable success, designing such molecules is challenging, and the number of currently addressable ubiquitin E3 ligases is limited. Here, we design a heterobifunctional *de novo* protein to trigger the degradation of a common cancer target, resulting in a desired phenotypic output. First, we developed a highly stable and adaptable helix–turn–helix scaffold for presenting multiple binding sites. Next, we use computational protein design to incorporate and embellish hot-spot-binding sites to target the antiapoptotic mediators BCL-x_L_ and MCL-1. We show a 75% success rate for creating submicromolar binders against these targets. Crystal structures of the complexes confirmed the designed binding poses. Then, we designed short linear motifs (SLiMs) into the loop of the scaffold to recruit KLHL20 and the ubiquitin ligase machinery. These designs have low micromolar affinity for KLHL20 comparable to that of the natural SLiMs. Moreover, the bifunctionalized proteins degrade BCL-x_L_ in cells, leading to apoptosis.

## Graphical abstract

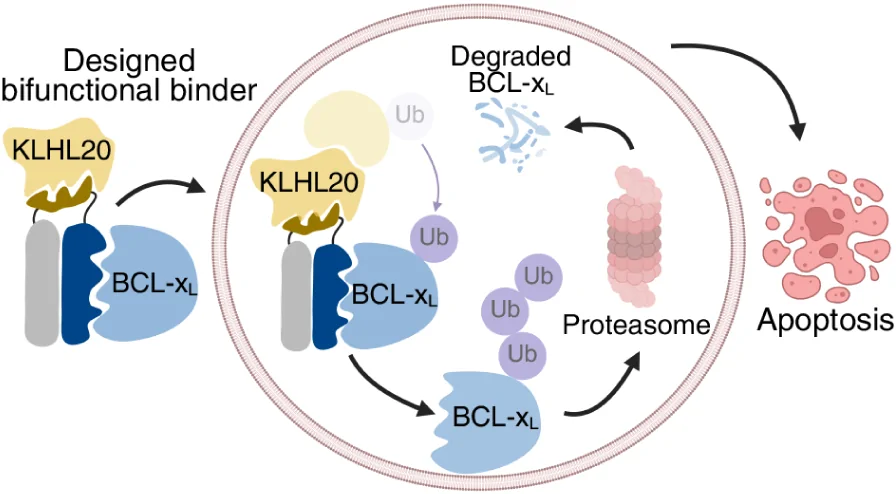

## Introduction

Over the past two decades, targeted protein degradation (TPD) has developed into a valuable tool for removing specified proteins in cells, including as a promising therapeutic strategy.^1,2^ While multiple degradation machineries are under investigation,^3,4^ the proteasome remains the most extensively studied.^1,2,5^ For instance, molecular glue degraders^6^ and proteolysis-targeting chimeras (PROTACs)^7^ simultaneously bind a protein of interest (POI) and an E3 ubiquitin ligase (E3), inducing the ubiquitination of the POI and its subsequent proteasomal degradation. Unlike classical inhibitors, PROTACs can work by engaging any region of the POI and are not limited to the recognition of functional sites. Through this encounter-driven mechanism, the process can be catalytic, with multiple copies of a POI being eliminated by each PROTAC molecule.^1,2,8^ As a result, for therapeutic applications, lower doses of cytotoxic compounds can be used.^9^

Despite encouraging progress in clinical trials,^5^ bottlenecks remain for small-molecule PROTACs. The degradation efficiency is driven by ternary complex formation, which depends on multiple factors, including individual binding constants of the two recognition elements, linker lengths between them, the locations of binding sites on the targets, and the proximity of suitable lysine residues to which ubiquitin can be conjugated.^8^ Second, despite the vast potential of the E3 repertoire, most PROTACs harness just two E3s—Cereblon (CRBN) and von Hippel-Lindau (VHL) protein.^5,10,11^ While this is changing with new E3 ligands being described,^12–14^ exploring new modes of targeting and binding the POI and ligases will help advance and generalize the technology.^1^ For instance, peptide-based PROTACs^15^ have the potential to overcome these limitations by using natural short linear sequence motifs (SLiMs) as common mediators of protein–protein interactions (PPIs) to access more targets and E3s.^7,16–18^ However, most SLiMs involved in degradation pathways—often referred to as degrons—are intrinsically disordered which presents challenges to direct, precise, and stable complex formation. Recent advances in protein-structure prediction and protein design could address these limitations.

To summarize the latter, deep learning-based methods have accelerated the field dramatically,^19–22^ with AlphaFold2 ^23^ and related methods^24^ for rapid and accurate modeling, and tools such as RFdiffusion^25^ and ProteinMPNN^26^ for *de novo* protein backbone and sequence design, respectively. User-friendly computational design pipelines, such as BindCraft,^27^ are achieving protein binders with affinities approaching the picomolar range.^27–29^ These successes have encouraged the field to move beyond binding a single protein toward multifunctional proteins observed in nature. For example, proteins have been designed to inhibit multiple different POIs^30^ or to display multiple viral epitopes.^31^ Designed proteins have been fused together via a flexible linker to drive the internalization of membrane proteins^32^ or to cause degradation or stabilization of intracellular targets.^33^ Also related to the work presented here, peptides designed with PepMLM, a fine-tuned version of the ESM-2 protein language model, have been linked to the catalytic domain of the CHIP E3 ligase leading to the degradation of Huntington’s disease-related proteins.^34^ Nonetheless, these strategies do not leverage the full potential of protein design, which, in principle, allows for the precise positioning of multiple binding sites to drive specific ternary complex formation. This is what we attempt to here.

Specifically, we describe a *de novo*-designed protein-based PROTAC. For the POI, we target BCL-x_L_, a member of the therapeutically important protein family of apoptosis regulators, namely, the B-cell lymphoma 2 (BCL-2) family. BCL-x_L_ is an antiapoptotic protein that can be inhibited by binding helical peptide effectors leading to apoptosis.^35,36^ As this interaction is well characterized with high-resolution X-ray crystal structures,^37^ together with homologues such as MCL-1 it has served as a testing ground for developing methods leading to the design of multiple peptide-, protein-, and small-molecule-based binders.^38–41^ Moreover, due to interest in BCL-x_L_ as an antiapoptotic cancer target, small-molecule inhibitors and PROTACs have been developed for it;^42^ e.g., a synthetic PROTAC molecule, DT2216, is currently undergoing clinical trials.^43^ Like many PROTACs, this uses a VHL-binding ligand to induce ubiquitination. Recently, we have identified other E3s compatible with TPD of BCL-x_L_, including the adapter Kelch-like protein 20 (KLHL20) with an in-cell, GFP-based degradation assay.^44^ X-ray crystal structures^45^ indicate that the KLHL20-binding competent SLiM adopts a compact loop conformation. Indeed, it has been reported that binding affinity can be improved by constraining the SLiM as a cyclic peptide, and these have been used in the design of small-molecule/peptide hybrid PROTACs.^17^

To generate and validate a *de novo* protein-based PROTAC against BCL-x_L_, we develop the following design pipeline (Figure 1a). First, using rationally seeded computational protein design,^46^ we make a straightforward helix–loop–helix scaffold. Next, we add a binding site for BCL-x_L_ using hot-spot grafting combined with ProteinMPNN optimization and AlphaFold2 modeling and evaluation. To test the versatility of the scaffold and approach, we design and compare binders for BCL-x_L_ and MCL-1, exploring specificity with *in vitro* binding assays and structural studies. Subsequently, we graft on a SLiM for KLHL20 and optimize its binding affinity. These binding sites were tested individually in the above in-cell, GFP-based degradation assay for their capacity to recruit the degradation target, BCL-x_L_, or the E3 ligase adapter, KLHL20. Finally, the best-performing binding sequences were combined and assessed for their ability to degrade BCL-x_L_ and induce apoptosis in a lung cancer cell line. Specifically, we compare single and bifunctional constructs with binding sites against BCL-x_L_ and/or KLHL20, and the small molecule in clinical trials, DT2216. Only the constructs with both modalities—i.e., for target and E3 ligase binding—function as BCL-x_L_ degraders in cells.

**Figure 1:**
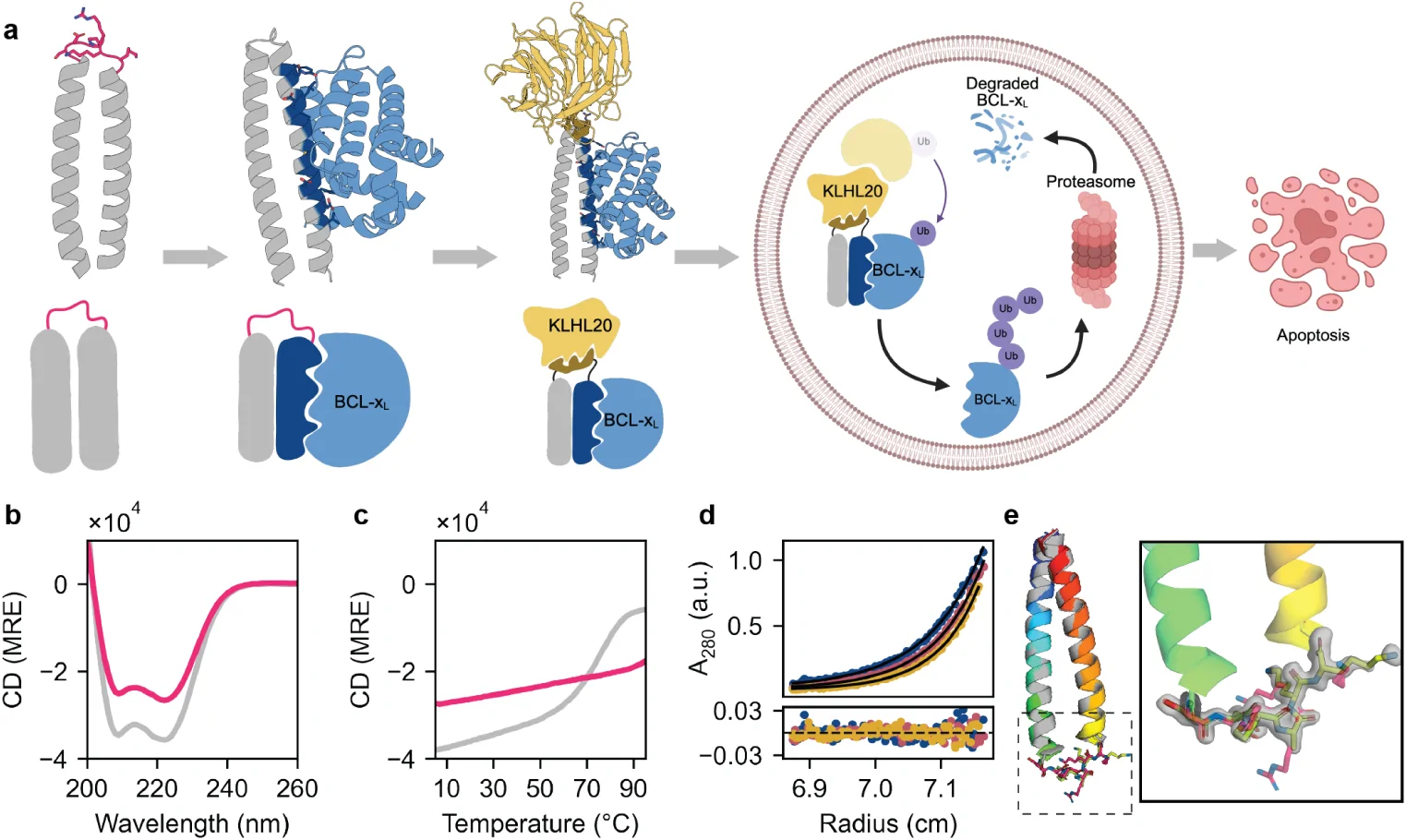
*De novo* design of a scaffold for the display of multiple binding motifs. **a**, Schematic overview of this work. From left to right: helix–loop–helix scaffold (sc-apCC-2) designed based on the heterodimeric, antiparallel, coiled-coil peptides, apCC-Di-AB;^47^ grafting of the BCL-x_L_ binding site; addition of a second interaction site for the E3 ligase adapter, KLHL20, into the loop; and testing for BCL-x_L_ degradation and apoptosis in human cell lines. **b**, Circular dichroism (CD) spectra for apCC-Di-AB (gray) and sc-apCC-2 (magenta). CD spectra were recorded at 20 °C and 50 μM total peptide/protein concentrations. **c**, Thermal response of CD signals at 222 nm for apCC-Di-AB (gray) and sc-apCC-2 (magenta), at 50 μM and 10 μM, respectively. **d**, Sedimentation-equilibrium analytical ultracentrifugation measurements at 52,000 rpm (blue), 56,000 rpm (red), and 60,000 rpm (yellow). The ratio between experimentally fitted and theoretical mass was 0.9 over the three speeds. **e**, X-ray crystal structure of the sc-apCC-2 scaffold, electron density around the loop shown at sigma level 1 (PDB ID: 9TLL; 1.5 Å). Parts of this figure were made with BioRender.

## Results

### Rational Design Yields a Highly Stable Display Scaffold

To create a small, highly thermostable, and highly designable scaffold for displaying helical target-binding sites and additional sites for E3 recruitment, we applied our rationally seeded peptide-to-protein approach.^46^ Specifically, we generated a helix–loop–helix scaffold from a previously characterized antiparallel, heterodimeric *de novo* coiled coil, apCC-Di-AB.^47^ Starting with the X-ray crystal structure of apCC-Di-AB, motif searching with MASTER^48^ was used to find loops from the PDB to connect the two helices. Next, the sequence of one loop and flanking residues was optimized for the scaffold using Rosetta remodel^49^ (Supplementary Figure 1). Initially, this designed hairpin was made by Fmoc-based solid-phase peptide synthesis. Unlike the dimeric parent peptide, this was highly thermostable, as confirmed by CD spectroscopy. An X-ray crystal structure at 1.5 Å resolution (PDB ID: 9TLL) had an overall backbone RMSD of 0.36 Å between the design model and experimental structure, and it confirmed the loop design. We called this new 67-residue miniprotein scaffold, sc-apCC-2, using our systematic naming system.^46^

### ProteinMPNN-Embellished Motif Grafting Gives Robust Binders to the POI

Previously, we have shown that dimeric coiled-coil peptides can bind to BCL-2 family proteins by grafting hot-spot residues onto the outer helical surfaces.^50,51^ To improve on this, we added modern AI tools such as ProteinMPNN^26^ and AlphaFold2 ^23^ to the following computational design pipeline, which is depicted in Figure 2a. To seed binder designs, first, we examined experimental structures of natural binders and targets in the BH3-only BCL-2 family and identified six hot-spot positions. To generate starting sequences for these sites, we used a small amino acid palette reflecting the sequences of BCL-2 effector proteins. For example, to target BCL-X_L_ binding, we started with 18 different sequence motifs (Supplementary Table 10) and grafted these onto 3 plausible locations on the solvent-exposed surface of the *de novo* scaffold. Models for each of the resulting 54 seed sequences were aligned onto 5 computationally generated models for BCL-x_L_:effector complexes where the pose of the effector peptide was rotated incrementally by 10° (Supplementary Figure 2), leading to 270 models in total. Next, to explore the potential to extend the complementary binding surfaces, we applied ProteinMPNN-RosettaRelax protocols^26^ and varied the number of residues in the scaffold to be mutated. This yielded 609 different sequences. For each of these, AlphaFold2 was used to predict complexes with BCL-x_L_. These models were filtered and assessed based on pLDDT, different AlphaFold2 metrics, shape complementarity (Sc),^52^ and Rosetta energy (ΔG)^53^ to select candidates for experimental testing. We repeated this protocol for MCL-1 as a secondary target. While also a member of the BCL-2 family, it has a distinct selectivity profile and frequently shows increased levels in response to BCL-x_L_ inhibition.^54^

**Figure 2:**
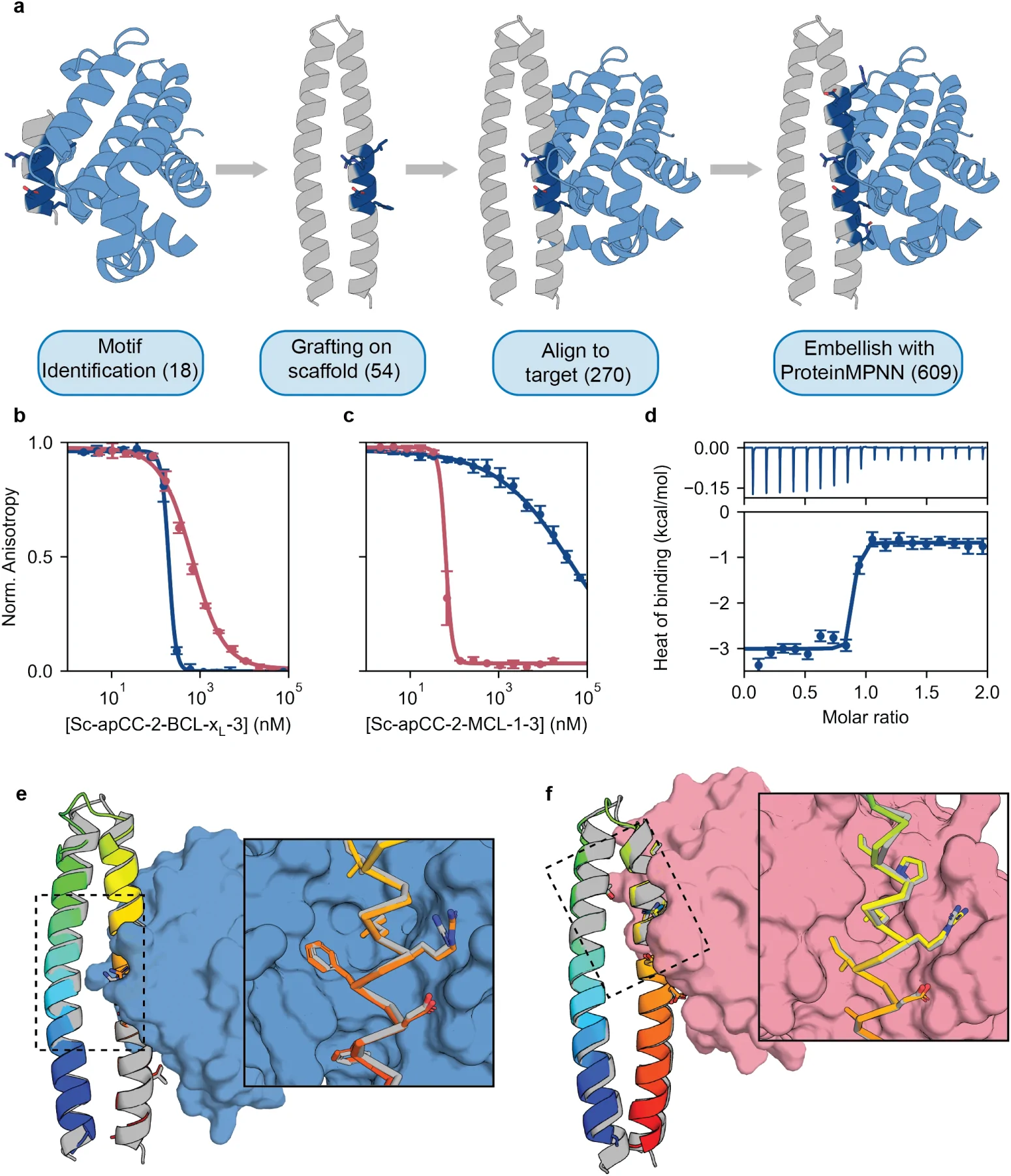
*De novo* designed binders for BCL-2-family proteins. **a**, Design schematic. From left to right: hot-spot residues selected from natural BCL-x_L_ and MCL-1 effectors were grafted onto the scaffold at all feasible surface-exposed locations of the scaffold and then aligned to the binding cleft; ProteinMPNN was used to add residues at the interface to improve binding. All final designed sequences were predicted as complexes with the target using AlphaFold2. Numbers in brackets refer to the motifs/designs at each step in the pipeline. **b**, Fluorescence anisotropy (FA) data for a BCL-x_L_ binder against BCL-x_L_ (dark blue, IC_50_: 196 ± 14 nM) and MCL-1 (red, IC_50_: 660 ± 50 nM). **c**, Similar FA data for an MCL-1 binder against MCL-1 (red, IC_50_: 64 ± 2 nM) and BCL-x_L_ (dark blue, IC_50_: 40 ± 20 μM). **d**, ITC data and analysis for the BCL-x_L_ binder against BCL-x_L_ (K_D_: 3 nM. **e**, Co-crystal X-ray structure of the BCL-x_L_ binder with BCL-x_L_ (PDB ID: 9TLU). The experimental structure of the sc-apCC-2 component (shown in rainbow colors) is aligned with its predicted model (gray), and the experimental structure of BCL-x_L_ is rendered as a surface (blue). The zoom shows details of the interacting side chains; the noninteracting helix and side chains are not shown for clarity. **f**, Similar images to **e**, but for the cocrystal X-ray structure of the MCL-1 binder and MCL-1 (PDB ID: 9TLV). The scaffold components in **e** and **f** are aligned vertically to highlight the shift in the designed binding sites.

We chose 14 designs against the primary target BCL-x_L_ and 4 against MCL-1 for expression from synthetic genes in *E. coli*. All but one of these could be purified as soluble proteins, and the remaining 17 were highly thermostable helical proteins (Supplementary Figure 3). We determined X-ray crystal structures for several of these designs at resolutions in the range of 1.15 – 2.6 Å (PDB IDs: 9TLM, 9TLN, 9TLO, 9TLP, 9TLQ, 9TLR, Supplementary Table 16, and Supplementary Figures 4–5). These revealed that the surface mutations were readily accommodated by the scaffold.

Initial binding to both targets was probed using a competitive fluorescent anisotropy (FA) assay with the natural effector peptide, BID, as the labeled competitor peptide that was displaced (K_D_ = 71 nM against BCL-x_L_ and K_D_ = 21.5 nM against MCL-1, Supplementary Figure 6 and Supplementary Table 11).^50,51^ Twelve of the expressed designs had sub-μM IC_50_ values against their intended targets, near the edge of the dynamic range for this assay (Supplementary Table 12). The MCL-1-targeting sequences were highly selective (Supplementary Figures 3 and 4 uppermost panels), while nearly all BCL-x_L_ targeting designs bound both targets equally (Supplementary Figure 3).

Isothermal titration calorimetry (ITC) was used to obtain a K_D_ value for BCL-x_L_ binding to a selected binder, Sc-apCC-2-BCL-x_L_-3. This binder had an IC_50_ of 196 ± 14 nM by FA and a K_D_ of 3 nM at 37 °C by ITC (Figure 2d and Supplementary Table 14). The difference between the two values arises because the K_D_ from ITC is a direct measurement, whereas the IC_50_ is an indirect measure from a competition assay. A 3 Å resolution X-ray crystal structure for this complex revealed that side chains in the binding interface matched the predicted conformation (Figure 2e; PDB ID: 9TLU). Crystal structures of two MCL-1 binders in complex with the target were also obtained (Figure 2f and Supplementary Figure 7; PDB IDs: 9TLV (2 Å resolution) and 9TLW (2.8 Å resolution)), likewise confirming the accuracy of the designs and AlphaFold2 predictions.

### Computational Negative Selection Improves Selectivity for the POI

Presumably due to the similarity between the peptide-binding interfaces on MCL-1 and BCL-x_L_, the designed BCL-x_L_ binders showed little specificity. To improve this, we added the following computational negative-selection step to identify selective designs for BCL-x_L_. AlphaFold2 was used to model the complexes of all previous designs with both target proteins (Figure 3a). These models were then scored and ranked based on the pLDDT of the motif residues, overall pAE score, and Sc. Sequences with large differences in rank for each metric and a combined metric were selected (Figure 3b and Supplementary Figure 8). In total, four further designs for selective targeting of BCL-x_L_ and two for MCL-1 were expressed in *E. coli*. Five of these could be purified as soluble proteins. Their biophysical characteristics were similar to the foregoing designs (Supplementary Figure 9). From the FA assay, the BCL-x_L_ binder selected by Sc, Sc-apCC-2-BCL-x_L_-17, retained a high inhibitory potency against BCL-x_L_ (IC_50_ = 52 ± 3 nM); moreover, it inhibited MCL-1 >1000-fold more weakly (IC_50_ = 68 ± 80 μM, Figure 3c). The other designs selected using the other metrics—pLDDT and pAE, and a combination of these plus Sc—either showed little specificity or had decreased affinity for both targets (Supplementary Figures 7 and 8). Thus, ProteinMPNN-enhanced motif grafting with some filtering—in this case based on shape-complementarity scores—can generate tight and selective binders. That said, while the number of designs tested here is limited, these data suggest that current confidence-based metrics do not reliably distinguish between candidates for experimental testing. Clearly, further work is needed in the field to assess computational designs more effectively and to make pipelines like those outlined here more predictive.

**Figure 3:**
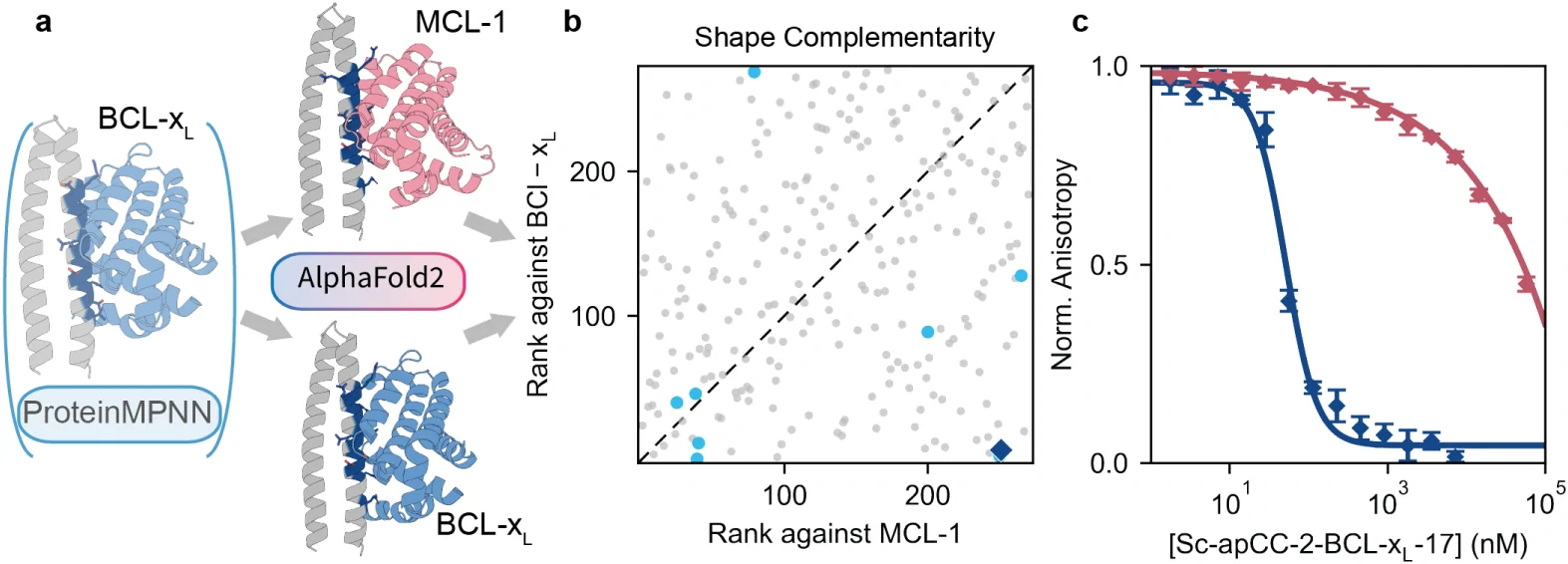
Designing selective binders for BCL-x_L_ through computational negative selection. **a**, Schematic of the selectivity procedure. After the ProteinMPNN design step against the desired target, AlphaFold2 was used to predict the complex with both targets, MCL-1 (red) and BCL-x_L_ (blue). **b**, Designs were ranked for binding to both targets based on metrics such as shape complementarity (Sc, shown in this scatterplot; for others Supplementary Figure 6). Designs predicted to be selective for BCL-x_L_ are located in the bottom right corner. The design selected based on Sc is shown as a dark blue diamond, and all previously tested anti-BCL-x_L_ designs as cyan circles. **c**, FA binding data for this BCL-x_L_ selective design based on Sc, against BCL-x_L_ (dark blue, IC_50_: 52 ± 3 nM) and MCL-1 (red, IC_50_: 68 ± 80 μM).

### E3 Targeting by Grafting a SLiM for KLHL20 into the Designed Loop

With potent BCL-x_L_ binders in hand, we turned to E3-targeting. The SLiM for KLHL20 adopts a turn-like conformation upon binding.^45^ Therefore, we hypothesized that grafting the SLiM into the loop between the two helices of sc-apCC-2 might promote an active conformation and improve binding affinity. We replaced the loop with the natural SLiM in two different lengths (short, 5 residues; or long, 11 residues) based on previous studies of KLHL20 binding,^45^ and we predicted models for the complexes with KLHL20 using AlphaFold2. Encouragingly, the positions of the SLiM hot-spot residues matched those in a known KLHL20/DAPK1 peptide complex.^45^ However, the confidence of these initial predictions was low with a pLDDT of the grafted motif of less than 60. Therefore, we applied the ProteinMPNN-RosettaRelax protocol keeping the four central hot-spot residues of the SLiM fixed. This increased the pLDDT to >90 (Figure 4a).

**Figure 4:**
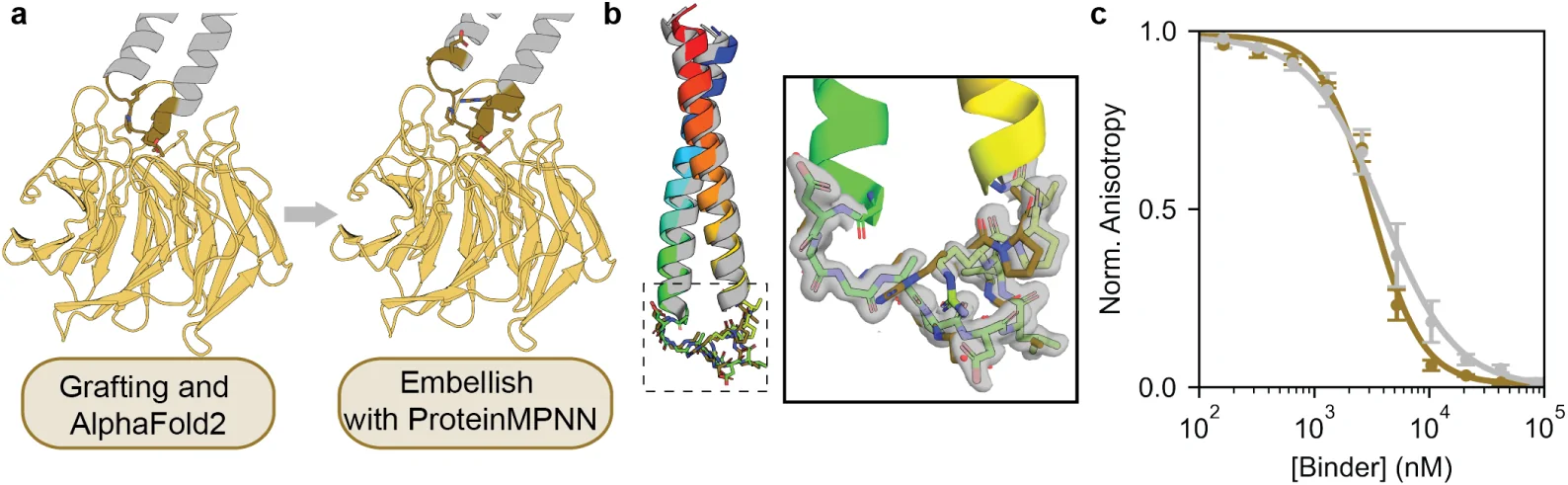
Incorporation of a SLiM for E3 ligase recruitment into the *de novo* scaffold. **a**, A natural SLiM sequence (brown) was grafted into the loop of the scaffold, and AlphaFold2 was used to predict the complex with the recognition domain of KLHL20. ProteinMPNN was used to alter residues close to the target. **b**, X-ray crystal structure of the sc-apCC2-KLHL20–3 (ProteinMPNN short-loop design; PDB ID: 9TLS), electron density shown around the loop at sigma level 1. **c**, FA data for KLHL20 binding with the natural short-loop design (gray) (IC_50_: 3.9 ± 0.2 μM) and the ProteinMPNN short-loop design (brown) (IC_50_: 3.16 ± 0.18 μM).

Both the natural and optimized sequences were tested experimentally with two designs for each length. Biophysical characterization and X-ray crystal structures confirmed that the SLiM-based loops were tolerated (Figure 4b; Supplementary Figures 10 and 11; PDB IDs: 9TLS (1.45 Å) and 9TLT (1.95 Å); Supplementary Table 16). In a competitive FA-binding assay for KLHL20 inhibition, all designs had low micromolar IC_50_ values (direct titration of the tracer indicated a K_D_ of 12 μM, see Supplementary Figure 5, similar to previous reports^45^). Thus, the binding sequence can be grafted into the loop of sc-apCC-2 to yield KLHL20 binders without compromising other scaffold properties. Although the designs with the longer, 11-residue loops had higher potencies as did the ProteinMPNN-optimized designs relative to the natural sequences, the differences were small with all IC_50_ values in the low μM range (Supplementary Figure 10; Supplementary Table 13). While the binding affinity for KLHL20 is only in the micromolar range, it is comparable to that observed for natural binders that are sufficient to cause protein degradation.^17,44^

### BCL-x_L_ and KLHL20 Binders Recruit Their Targets in a GFP-Based Degradation Assay

To test the capacity of the monofunctional BCL-x_L_ and KLHL20 binders to recruit their targets, we used a previously established GFP-based degradation assay in HEK293 cells.^44^ This follows the degradation of transiently expressed protein and allows a medium-throughput assessment of individual binders to identify those that could be combined in a single construct. In brief, to test the BCL-x_L_ binders, HEK293 cells engineered to express a BCL-x_L_:GFP fusion protein stably were transfected with BCL-x_L_ binders fused to the full-length SPOP E3 ligase. 4 out of 8 binders showed significantly decreased GFP fluorescence compared to the negative control. To test the KLHL20 binders, the same HEK293 cells were transfected with the KLHL20 binders fused to an anti-GFP nanobody. All 4 binders lowered GFP fluorescence (Supporting Information Figure 12).

### Bifunctional Designs Are Potent Degraders of BCL-x_L_ and Induce Apoptosis

Next, with their degradation capacity validated individually, we combined the BCL-x_L_ and KLHL20 binding sites into a single scaffold and tested them for endogenous BCL-x_L_ degradation in a lung cancer cell line (A549). For this, we chose the most potent and best-performing BCL-x_L_ binder from the GFP-based degradation assay, sc-apCC2-BCL-x_L_-5, and combined it with the KLHL20-binding module, sc-apCC2-KLHL20-3. These were configured in two ways (Figure 5a): one with two binding motifs combined within a single *de novo* module, and a second with the two monofunctional modules linked in tandem (Supporting Information Figure 13). For negative controls, we used both *de novo* binding proteins individually. As a positive control, we used PROTAC DT2216, which comprises small-molecule ligands for BCL-x_L_ and VHL (Supporting Information Figure 14). A fluorescently labeled anti-BCL-x_L_ antibody was used to measure endogenous BCL-x_L_ levels.

**Figure 5:**
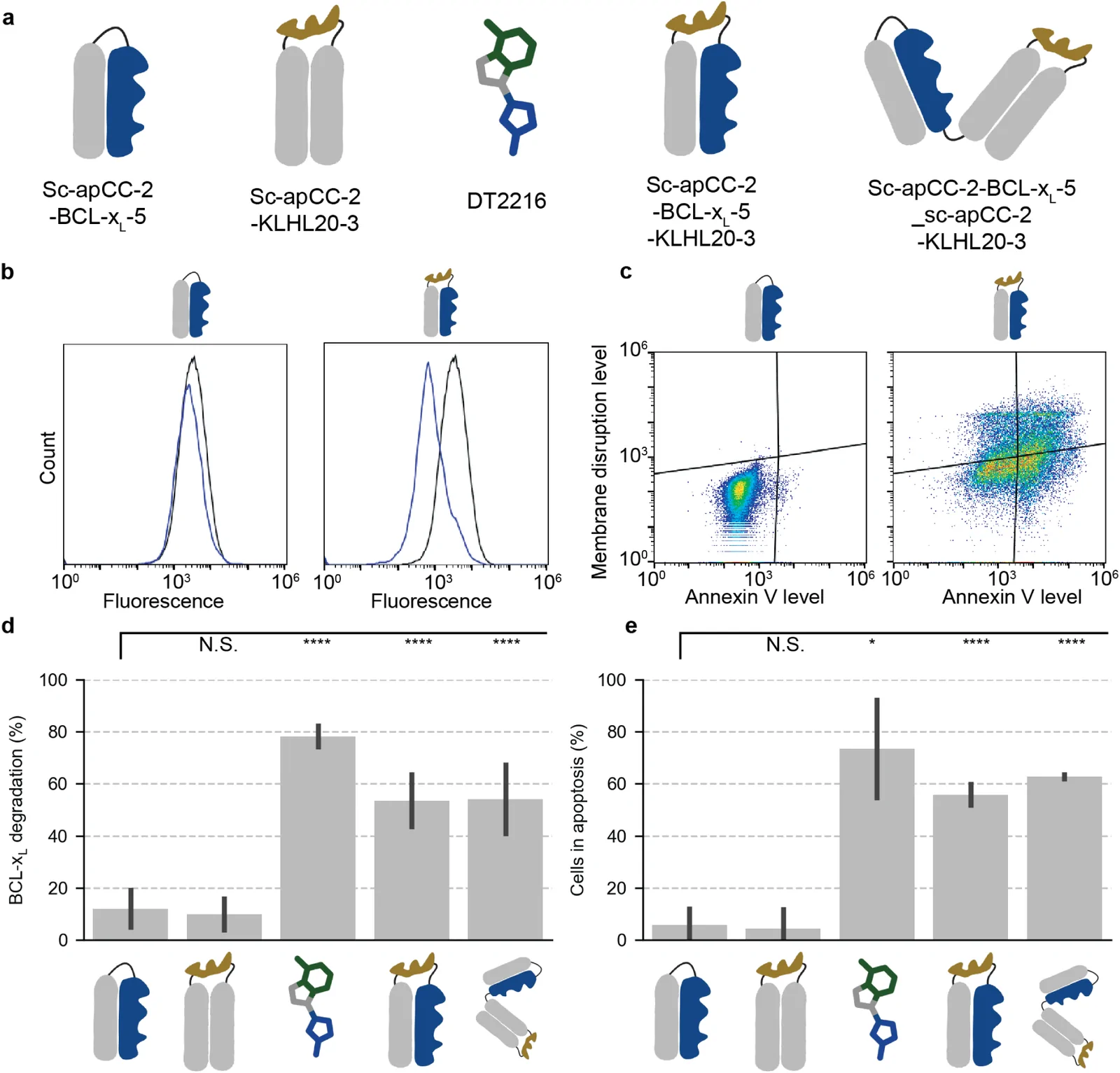
Bifunctional binders cause endogenous BCL-x_L_ degradation and apoptosis. **a**, Schematic of the molecules tested. All the designs use the same BCL-x_L_- and KLHL20-binding motifs. Negative controls are monofunctional (i.e., contain only the BCL-x_L_-binding motif (blue) or the KLHL20-binding motif (brown). Small-molecule PROTAC DT2216 was used as a positive control (the chemical structure is a cartoon representation). Two variants of the bifunctional designs were tested: one with both binding sites within a single scaffold protein and another with them separated into two different scaffolds connected by a flexible linker. **b**, Flow cytometry data for the endogenous degradation; representative data for the negative control and combined binders are shown. Histograms for the design (blue) and mCherry control (black). **c**, Flow cytometry data for the apoptosis assay, Annexin V levels on the *x*-axis, and the DAPI signal indicating membrane disruption on the *y*-axis. Representative data are shown for the negative control and the combined binder. **d**, Degradation assay for native BCL-x_L_ in the A549 cell line. BCL-x_L_ degradation levels were calculated by the Overton positive method, with an average of at least three biological repeats. Mean values: Sc-apCC-2-BCL-x_L_-5, 12%; Sc-apCC-2-KLHL20–3, 10%; DT2216, 78%; Sc-apCC-2-BCL-x_L_-5-KLHL20–3, 54%; Sc-apCC-2-BCL-x_L_-5_Sc-apCC-2-KLHL20–3, 54%. **e**, Apoptosis assay; bars show combined early and late apoptosis averaged over at least three biological repeats. Mean values: Sc-apCC-2-BCL-x_L_-5, 6%; Sc-apCC-2-KLHL20–3, 4%; DT2216, 73%; Sc-apCC-2-BCL-x_L_-5-KLHL20–3, 56%; Sc-apCC-2-BCL-x_L_-5_Sc-apCC-2-KLHL20–3, 63%. **d** and **e**, Significance levels were calculated using a Welch *t*-test relative to the negative control with only the BCL-x_L_ binding site. Significance levels are represented as N.S.: *P* > 0.05, ∗: *P* ≤ 0.05, ∗∗∗∗: *P* ≤ 0.0001. Parts of this figure were made with BioRender.

Neither of the separate monofunctional controls significantly decreased BCL-x_L_ levels (Figure 5b,d). However, both bifunctional designs showed significant decreases in BCL-x_L_ in a similar manner to DT2216 (Figure 5b,d). The addition of the proteasome inhibitor carfilzomib decreased the level of degradation significantly, indicating a proteasome-dependent mechanism (see Supporting Information Figure 15). Combinations of other BCL-x_L_ and KLHL20 binding sequences yielded similar results, highlighting the versatility of the scaffold and the binding site design (Figure 5d and Supplementary Figure 16).

Depleting BCL-x_L_ should increase apoptosis. To test this, we used another 2D flow cytometry assay to measure (i) externalized phosphatidylserine indicative of early-stage apoptosis using FITC-labeled Annexin V (*x*-axis), and (ii) cell viability using DAPI to monitor the loss of membrane integrity associated with late-stage apoptosis (*y*-axis)^55^ (Figure 5c). Overall apoptosis (combined early and late) mirrored the protein-degradation assay (compare Figure 5c,e): the individual binders did not increase apoptosis above baseline, while the bifunctional helix–loop–helix constructs showed significant increases. These results were mirrored in an ApoTox-Glo Triplex Assay, which assesses cell viability, cytotoxicity, and apoptosis events simultaneously (Supplementary Figure 17). Combined, these experiments show that transfection of plasmids encoding bifunctionalized protein scaffolds into live cells causes robust degradation and subsequent induction of apoptosis. While a direct comparison to the small-molecule PROTAC is difficult due to our use of transfection and, consequently, uncertain protein concentrations, our results indicate that we achieve similar levels of activity to those obtained using the recommended dose of the drug.

## Discussion

In summary, to develop *de novo* protein-based PROTACs, we report the computational design of a small, highly stable protein scaffold that presents multiple epitopes engaging different subcellular proteins. Using this, we introduce a binding site for the antiapoptotic, cancer target BCL-x_L_ and another for an E3 ubiquitin ligase adapter, KLHL20. Effectively, this *de novo* protein design brings these natural proteins together in cells to cause the degradation of BCL-x_L_ and increase apoptosis in a cancer cell line. The designed proteins are characterized fully *in vitro*, allowing them and the design methods to be evaluated thoroughly.

Recently, the incorporation of multiple, independent binding sites into a single *de novo* scaffold has been reported.^31^ Moreover, others have combined engineered or designed proteins to render the EndoTag and GPlad systems for clearing proteins by internalization into cells or via modification followed by proteolysis within cells, respectively.^32,33,56^ In the PROTAC field, polypeptides have been used as modules.^7^ Recently, protein design tools such as RF diffusion^25^ and ProteinMPNN^26^ have been used to generate bifunctional unstructured peptides that bind the androgen receptor and the E3 ligase VHL, leading to degradation of the former.^57^ The design of a structured helical dimer comprising a SOS mimetic and a p53 mimetic within a cross-linked dimer has been shown to engage the MDM2 E3 ligase and reduce levels of RAS.^58^

Our designs are distinct from peptide-based systems. While they are small (<80 amino acids), they have well-defined secondary and tertiary structures, are highly thermostable, and are capable of binding simultaneously to a target protein of interest and an E3 ligase adapter. For the former, we show that known helical binding sites for BCL-x_L_ and MCL-1 can be grafted onto a helix of our *de novo* scaffold and tuned with computational design to improve binding and specificity. For the latter, to engage KLHL20, we incorporated a binding site into the constrained loop of the scaffold without perturbing its structure or stability. Although we have only demonstrated this for two target-binding sites grafted into a single *de* novo-designed scaffold, we posit that such bifunctional proteins are promising platforms for future studies to screen target—E3 pairings and orientations. This *de novo*-scaffold-plus-binder-design approach might also be adapted to direct other post-translational modifications such as phosphorylation.^59^

To progress such proteins toward therapeutics, significant further steps would be needed. For instance, the designed proteins would have to be delivered across cell membranes and into cells. Encouragingly, we have shown that cationic variants of our coiled-coil peptides do cross cell membranes,^47^ and we believe that a similar strategy could be implemented for bifunctional designs.

Our approach also facilitates full biophysical and structural characterization of the designs, as demonstrated by a wealth of solution-phase data and several X-ray cocrystal structures that we report. In turn, these help to critically assess the designs and design methods. For instance, we use motif grafting embellished with computational sequence design using ProteinMPNN followed by AlphaFold2 structure predictions. In almost all cases, the addition of residues in direct contact with the targets improves the AlphaFold2 and physical-scoring metrics. Interestingly, and as reported by others,^26,28^ extending ProteinMPNN to residues outside of the binding site improves the AlphaFold2 confidence metrics, but it does not change the physical-scoring metrics significantly (Supplementary Figure 19). Moreover, improvements suggested by *in silico* modeling and assessment are not necessarily reflected in experimental measurements of binding constants. For example, for the BCL-2-family binders, extending the binding site beyond the natural, hot-spot motif proved essential to achieve better binding and selectivity. However, by contrast, for targeting the KLHL20-binding site, there was no improvement beyond using the natural (SLiM) sequence. This raises the potential problem of false positives in design pipelines. In addition, we find that none of the AI confidence predictors or physical-scoring metrics correlate well with the measured experimental binding affinities (Supplementary Figures 20–24). While our current data are not sufficient to give definitive recommendations on what metrics to use, recent large-scale *de novo* binder studies^60,61^ and other benchmarks^62^ agree that there is a need for better *in silico* predictors of binding potential and ideally for binding affinity. These are clear challenges for the field.

## Supplementary Material



## Notes

Materials and methods, sequences of peptides and proteins used in this study, tables of measured IC_50_, experimental details about X-ray crystallography experiments, extra figures explaining computational design steps, figures with biophysical characterization (CD, SEC, and FA) of all proteins, figures of all X-ray crystal structures obtained in the study, figures of the GFP degradation assay, figure of the protease inhibitor carfilzomib in the endogenous degradation assay, figure of endogenous degradation and apoptosis assay of additional constructs, figure of the ApoTox-Glo assay, figures comparing the measured IC_50_ with computational metrics, figures with additional computational metrics, figures on validation of used peptides, mass spectrometry, and analytical HPLC traces (PDF)

## Abbreviations

TPD: targeted protein degradation
PROTAC: proteolysis-targeting chimera
SLiM: short linear motifs
POI: protein of interest
E3: E3 ubiquitin ligase
CRBN: cereblon
VHL: von Hippel–Lindau
PPI: protein–protein interaction
BCL-2: B-cell lymphoma 2
KLHL20: Kelch-like protein 20
CD: circular dichroism
*E. coli*: *Escherichia coli*
Sc: shape complementarity
ΔG: Rosetta energy
FA: fluorescent anisotropy
ITC: isothermal titration calorimetry
