# Supplementary material for: De Novo-Designed Bifunctional Proteins for Targeted Protein Degradation
Source: J Am Chem Soc. 2026 Aug 5;148(32):34311–9. doi: 10.1021/jacs.6c07593 (PMC13495755; doi:10.1021/jacs.6c07593)
Supplement: Supplementary file 1 [file ja6c07593_si_001.pdf]

Supporting Information for:

De novo designed bifunctional proteins for targeted protein degradation

Bram Mylemans[1,2], Boguslawa Korona[3], Amanda M. Acevedo-Jake[4], Ailsa MacRae[4], Thomas A. Edwards[5], Danny T. Huang[6,7], Andrew J. Wilson[4,8,9*], Laura S. Itzhaki[3*] and Derek N. Woolfson[1,2,10,11*]

[1]School of Chemistry, University of Bristol, Cantock's Close, Bristol BS8 1TS, UK

[2]Novo Nordisk Foundation Center for Protein Design, Department of Biology and Department of Drug Design and Pharmacology, University of Copenhagen, Jagtvej 160, 2100 Copenhagen, DK

[3]Department of Pharmacology, University of Cambridge, Cambridge CB2 1PD, UK

[4]School of Chemistry, University of Birmingham, Edgbaston, B15 2TT, UK

[5]College of Biomedical Sciences, Larkin University, 18301 N Miami Ave #1, Miami, Florida, 33169 USA

[6]Cancer Research UK Scotland Institute, Garscube Estate, Switchback Road, Glasgow G61 1BD, UK

[7]School of Cancer Sciences, University of Glasgow, Garscube Estate, Switchback Road, Glasgow G61 1QH, UK

[8]School of Chemistry, University of Leeds, Woodhouse Lane, Leeds LS2 9JT, UK

[9]Astbury Centre for Structural Molecular Biology, University of Leeds, Woodhouse Lane, Leeds LS2 9JT, UK

[10]School of Biochemistry, University of Bristol, Medical Sciences Building, University Walk, Bristol BS8 1TD, UK

[11]Max Planck-Bristol Centre for Minimal Biology, University of Bristol, Cantock's Close, Bristol BS8 1TS, UK

MATERIALS AND METHODS

Computational loop design

The last and first six residues from the A and B chain of the previously published crystal structure of apCC-Di-AB (PDB:7Q1T) respectively were used as a search query for MASTER[1]. The original database made available with the publication was used. Results up to 0.6 Å RMSD were separated by the number of residues in between the helices. Loops of 4 residues were the most common and the lowest RMSD hit (PDB id 2XPI residues 470-473) was chosen as the starting structure for remodeling. Starting from the aligned and inserted loop, 100 Rosetta remodel[2] trajectories were run, a total of 6 residues (the 4 loop residues and 2 flanking residues) were mutated to any amino acid but cysteine. The remaining sequence was fixed. The lowest energy model was selected for experimental testing.

Computational binding site design

For the BCL-x_L binder design, a selection of 6 residue motifs based on the natural binders (BAD and BIM) were created. All motifs have the following structure Φ_1...L..Φ_2XD.F with Φ_1 either Y,F or I, Φ_2 either F,I,M and X either G,S. The full list of motifs is shown in supplementary table 10. These motifs were grafted onto a modified model of sc-apCC-2, so that none of the core coiled-coil heptad positions (a and d) were altered. To accommodate the binding sites better, 4 residues were added to each helical termini, the R@a, D@e pairs were split and charges were more evenly

distributed at e and g heptad positions as is the case in apCC-Di[3]. This scaffold could accommodate the motifs at 3 locations resulting in a total of 54.

These models were aligned on 5 slightly modified AlphaFold2 predictions of natural effector:protein complexes. A Rosetta script introduced a small angular shift in the effector binding angle, the resulting 5 models are shown in supplementary figure 2. Subsequently, 4 different strategies were used to modify the sequence with a ProteinMPNN-RosettaRelax pipeline: Strategy 1, no additional mutations were made; Strategy 2 non-motif, non-core and non-loop residues within 6 Å from BCL-x_L are allowed to mutate; Strategy 3 all non-motif, non-core and non-loop residues are allowed to mutate; Strategy 4 all non-core residues are allowed to mutate (thus including the natural motifs and designed loop). The ProteinMPNN-RosettaRelax pipeline consisted of 3 cycles of ProteinMPNN at a temperature of 0.2, grafting of the top sequence onto the helix-loop-helix and a short relax following the InterfaceRelax2019 script. After the final relax run, Rosetta scores including shape complementarity and interface ΔG were calculated. In addition, an AlphaFold2 prediction was made of each final sequence with and without a pre-generated MSA of BCL-x_L. Designs were filtered based on pLDDT (>85 as a monomer and >90 as a complex) and a final selection was made based pLDDT, pAE, ΔG or shape complementarity. All selected sequences with their respective strategy and selection method are shown in supplementary table 4.

For MCL-1, the same method was employed but only the MPNN implementation in strategy 2 was used. Instead of strategy 1, the entire previously described binding site[4] was grafted onto the modified sc-apCC-2 model. Selected sequences are shown in supplementary table 3.

For KLHL20, the previously designed loop was replaced with the natural SLiM sequences -AGLPDLVGA- (short) or -AGLGLPDLVAKYNGA- (long). AlphaFold2 models were predicted with a pre-generated MSA for KLHL20. Subsequently the same three cycle ProteinMPNN-RosettaRelax pipeline was used keeping the middle -PDLV- of the SLiM fixed.

All code and inputs are available from the labs Github page.

Computational selectivity screening

For the BCL-x_L and MCL-1 selectivity screening, AlphaFold2 predictions were made of the final sequences of strategy 2 with the opposite target's MSA. In addition, the final sequences were grafted on sc-apCC-2 and target complexes and subsequently relaxed and scored by Rosetta. For pLDDT, pAE and Shape Complementarity the rank of each sequence against each target was determined, and sequences with the largest difference in rank favoring the desired target were selected. All code and input are available from the lab's Github page.

Solid-phase peptide synthesis and purification

All peptides used in this study (see Supplementary Table 1) were synthesized following standard Fmoc automated microwave solid-phase peptide synthesis procedures with a Liberty Blue (CEM) as described before[5,6]. N,N'-diisopropylcarbodiimide (DIC) and Oxyma Pure in N,N-dimethylformamide (DMF) were used as activators and a 20% (v/v) solution of morpholine or piperidine in DMF was used in the deprotection step. Peptides with acetyl (Ac) caps were treated with pyridine or diisopropylethylamine and acetic anhydride. 5-carboxyfluorescein (FAM) coupled peptides were prepared by coupling of 6-(amino)-hexanoic acid and FAM with the standard DIC and Oxyma Pure in DMF mixture. Peptides were cleaved from the resin with a trifluoracetic acid (TFA):water:triisopropysilane mixture in 95:2.5:2.5 (v/v) ratio at room temperature. Subsequently, cleaved peptides were precipitated with ice-cold diethyl ether.

The freeze-dried peptides were dissolved in a water/acetonitrile mixture and purified with reverse-phase HPLC using Water and acetonitrile with 0.1% (v/v) TFA as solvents. Peptide purity was assessed by analytical HPLC (see Supplementary Fig. 25, 31, 33, 35, 37), and matrix-assisted laser desorption/ionization-time of flight (MALDI-TOF) mass spectrometry (see Supplementary Fig. 25, 30, 32, 34, 36).

Protein expression and purification

The genes encoding the designed binders and target proteins (BCL-x_L, MCL-1) were cloned into a pet28a vector with Kanamycin resistance. *E. coli* BL21 DE3 cells were transformed with the plasmids (see Supplementary Tables 2-7 for protein sequences). Cells were grown in Luria Broth at 37° C until an optical cell density of 0.6 was reached. Protein production was induced by

addition of isopropyl-β-D-thiogalactoside and cultures were kept at 20 °C overnight. Afterwards, the cells were harvested by centrifugation and frozen until further use.

For purification of the binders, the cells were resuspended in binding buffer (25 mM Tris, 300 mM NaCl, 10 mM imidazole pH 8.0) and lysed by sonication. The lysate was clarified by centrifugation. The supernatant was applied on a His-trap column equilibrated with binding buffer and washed with (30 mM imidazole. The protein was eluted with 300 mM imidazole. Subsequently, the imidazole was removed by a PD-10 desalting column and incubated overnight with TEV protease at room temperature. After His-tag cleavage, the protein solution was reapplied on the His-trap column and flow-through was collected. This flow-through was applied on a HiLoad 16/600 superdex75 column equilibrated in biophysics buffer (25 mM Tris, 150 mM NaCl, pH 7.4). The peak containing the monomeric proteins was collected and concentrated for later use.

For BCL-x_L and MCL-1 protocols were followed as previously described[6]. The cells were resuspended in lysis buffer (25 mM Tris, 500 mM NaCl, pH 8.0 cOmplete, Mini, EDTA free protease inhibitor cocktail, Roche and 5 mg lysozyme and 3 mg DNAse, Roche). Afterwards, they were sonicated and clarified via centrifugation. The soluble fraction was applied on a His-trap HP column (Merck) pre-equilibrated with wash buffer (10 mM imidazole, 25 mM Tris, 500 mM NaCl, pH 8.0). The column was then washed four times with increasing concentration of imidazole up to 100 mM, before a final elution with 400 mM imidazole. The protein solution was dialyzed overnight together with His-Ulp1 to remove the SUMO-tag. The next day the solution was applied on the His-trap HP column and the flow-through was collected. This flow through was collected

and passed-through size exclusion chromatography with a HiLoad 26/60 Superdex 75 column. This column was previously equilibrated with 20 mM Tris 250 mM NaCl, 0.5 mM DTT, 2.5% glycerol, pH 8.0. The fractions containing the monomeric protein were pooled and concentrated before storage for later use. Protein purity and quality was assessed by mass spectrometry (see Supplementary Fig. 26-27) and CD (see Supplementary Fig. 29).

For KLHL20 expression and purification, Rosetta™ (DE3) Competent Cells (Novagen) were transformed with a KLH20 DNA construct (pNIC28-Bsa4-6his-KLHL20(303-605). Cells were grown to OD_{600} of 0.6 at 37 ºC, before the temperature was lowered to 18 ºC and protein expression was induced with 0.4 mM IPTG overnight. The cells were pelleted by centrifugation and resuspended in lysis buffer (50 mM HEPES pH 7.5, 500 mM NaCl 5% glycerol, 0.5 mM TCEP, 5 mg lysozyme, 3 mg DNAse and 1 cOmplete, Mini, EDTA free protease inhibitor cocktail tablet). Afterwards, the cells were sonicated and clarified by centrifugation. The supernatant was applied to 5 mL HisPur Ni-NTA resin (Thermo Fisher), preequilibrated with binding buffer (50 mM HEPES pH 7.5, 500 mM NaCl, 5% glycerol, 0.5 mM TCEP) and left rotating at 4 °C overnight. The flow through was collected using a peristaltic pump and the beads were washed four times with increasing imidazole concentration (10 mM -100 mM). Subsequently, the protein was eluted with 300 mM imidazole in the same buffer. The eluted protein was dialyzed overnight with TEV protease, before being applied again on the Ni-NTA beads. The cleaved KLHL20(303-605) was eluted with the same elution buffer. The protein was dialyzed into 50 mM HEPES pH 7.5, 300 mM NaCl, 0.5 mM TCEP, 2.5% (v/v) glycerol and concentrated before storage for later use.

Protein purity and quality was assessed by mass spectrometry (see Supplementary Fig. 28) and CD (see Supplementary Fig. 29).

Circular dichroism spectroscopy

Circular dichroism (CD) spectroscopy data were collected on a JASCO spectropolarimeter instrument fitted with a Peltier temperature controller. The binders were prepared to 20 μM concentration in 20 mM Tris 150 mM NaCl, pH 7.4 buffer unless otherwise stated in figure legends. Data were collected in a 1-mm quartz cuvette with a 200 nm to 260 nm wavelength. The following instrument settings were used: band width, 1 nm; data pitch, 1 nm; scanning speed, 100 nm/min; response time 1 s. Each spectrum is the background subtracted average of 8 scans. For the thermal denaturation scans, the circular dichroism signal was followed at 222 nm wavelength while increasing the temperature from 5 °C to 95 °C at a ramp rate of 1 °C/min. BCL-x_L, MCL-1 and KLHL20 were measured in 10 mM K_2HPO_4 adjusted with NaOH to pH 7.4 at 50 μM protein concentration. The spectra were normalized to mean residue ellipticities (deg cm^2 $dmol^{-1}res^{-1}$) by normalizing for concentration of peptide bonds and the cell path length using equation MRE = $\frac{\theta \times 10^6}{c \times l \times n}$ with θ the measured difference in absorbed circularly polarized light in mdeg, c the concentration in μM, l the path length in mm and n the number of amide bonds in the proteins.

Analytical size exclusion chromatography

Analytical size exclusion chromatography was performed on a JASCO HPLC system with a superdex 75 increase 10/300 column. 20 μL of protein solution at a concentration of 100 μM in 20

mM Tris, 150 mM NaCl, pH 7.4 was injected at 0.5 mL/min. The UV absorbance at 230 nm was recorded, the background was subtracted and the normalized signal was plotted in function of elution volume.

Analytical ultracentrifugation

Sedimentation equilibrium experiments were performed on a Beckman Optima X-LI analytical ultracentrifuge with an An-60-Ti rotor (Beckman-Coulter) and data was fitted using SEDPHAT software[7] to single-species model. Monte Carlo analysis was performed to yield 95% confidence limits. A peptide concentration of 100 µM in PBS (8.2 mM sodium phosphate dibasic, 1.8 mM potassium phosphate monobasic, 137 mM NaCl, 2.4 mM KCl, pH 7.4) was used. The sample was spun at 52000, 56000 and 60000 RPM after 8-hour equilibration at each speed, the measurement at each speed was duplicated.

Competitive fluorescence anisotropy assay

The competitive fluorescence anisotropy experiment were performed following previously described procedures[6]. Each experiment was run in triplicate and the fluorescence anisotropy measured using a CLARIOstar plus plate reader (BMG Labtech), with excitation at 482nm

(bandwidth = 16nM), emission at 530nm (bandwidth = 40nm) and polarized dichroic mirror at 504nm. For the direct titration assay, stock concentrations of 1mg/ml peptide were made in DMSO and then further diluted to a working stock of 100 nM in FA buffer (20 mM Tris, 150 mM NaCl pH 7.4 1 mM DTT 0.01% triton x100) for use in the assay. For MCL-1 and BCL-x_L experiments, the target protein concentration was fixed at 150 nM and tracer concentration fixed at 25 nM. For KLHL20 experiments, KLHL20 concentration was fixed at 8 μM and 83.3 nM tracer concentration. The competitor protein was titrated in.

Perpendicular (P) and parallel (S) intensities of the control wells were averaged and deducted from each corresponding samples to give the corrected values, P_{corr} ($P_{corr} = P_{sample} - P_{av.\ control}$) and S_{corr} ($S_{corr} = S_{sample} - S_{av.\ control}$). The total sample intensities (I) and anisotropies (r) were calculated with the follow equations: $I = (2 \times P_{corr}) + S_{corr}$, $r = (S_{corr} - P_{corr}) / I$

The points for each competition curve were normalized between 0 and 1. The obtained values were plotted against competitor concentrations and fit to a logistic function of the form $Y = A2 + (A1 - A2) / (1 + (x/IC_{50})^p)$ to determine the half-maximal inhibitory concentration (IC_{50}) of the peptide competitor (where A2 is the upper asymptote and A1 the lower asymptote).

Isothermal Titration Calorimetry

Isothermal titration calorimetry (ITC) experiments were carried out using a MicroCal PEAQ-ITC system (Malvern Panalytical) at 37 ˚C. A stock solution of BCL-xL was dialyzed into ITC buffer overnight (20 mM Tris, 150 mM NaCl pH 7.4), and the same buffer was employed to dissolve a solid portion of the tested binder. For each experiment, initially, the cell was filled with 275 μl of binder (20 μM) and the syringe was loaded with the BCL-xL at 200 μM concentration. An initial injection of 0.4 μL was then followed by 18 injections of 2 μL, every 150s, with a constant syringe rotation speed of 750 rpm throughout. The signal for the enthalpy of dilution of the protein into buffer was independently measured in a control titration experiment and then subtracted from the experimental binding traces. NITPIC[8] was used to integrate the injections. The data was then fitted with SEDPHAT software[7] to extract K_D, ΔG°, ΔH° and $-T\Delta S^{\circ}$ using an adjusted single-site binding model.

Crystallization trials

Protein crystals were grown using a sitting-drop, vapor diffusion method with commercial sparse matrix screens. 0.3 µL of a concentrated protein solution or 1:1 mixture of proteins in case of the binders was mixed with 0.3 µL of the screening solution in MRC 96 well plates. The plates were incubated at 20 °C until protein crystals were observed. The crystallization condition for each structure can be found in Supplementary table 15. The crystals were mounted and transferred to a cryoprotecting solution made of the screen supplemented to 25% glycerol before flash freezing in liquid nitrogen.

The proteins were diffracted at Diamond light source or the European Synchrotron Radiation Facility. Data processing was done on the beamline with either XDS[9] or Dials[10]. Aimless[11] was used for scaling. Molecular replacement was done with PHASER[12] using AlphaFold2 models. Phenix Refine[13] was used for refinement of the structures using translation/liberation/screw (TLS) parameters for each chain. Data and refinement quality statistics can be found in supplementary table 16.

Cell culture

All cell culture procedures were carried out according to standard procedures. In brief, all cell lines were maintained at 37%˚C in an atmosphere of 5% CO_2 and ≥95% humidity. HEK293T cells were cultured in Dulbecco's Modified Eagle Medium (DMEM; Gibco™) supplemented with 10% fetal bovine serum (FBS), while A549 cells were grown in Nutrient Mixture F-12 Ham medium (Merck) supplemented with 10% FBS. DNA transfections were performed using the cationic lipid reagent TransIT-2020 (Mirus Bio).

Stable GFP–Bcl-x_L cell lines (see Supplementary Table 8) were generated in a previous study (accompanying paper) through antibiotic selection followed by sorting of live GFP-positive cells for subsequent propagation.

Flow cytometry assay of degradation of GFP-BCL-x_L

BCL-x_L binders were synthetized in pcDNA3.1Hygro(+) plasmid (GenScript) in such a way that they are preceded by a single FLAG tag and followed by the SPOP E3 ligase and mCherry with a T2A self-cleaving peptide sequence. The KLHL20 binder constructs were in the same plasmid preceded by a FLAG tag and anti-GFP nanobody and followed by the same mCherry T2A combination. Full protein sequences are found in Supporting Table 8.

The flow cytometry assay was performed as previously described (see https://www.biorxiv.org/content/10.64898/2025.12.22.695764v2 and additional peer review document). Briefly, on day 1, cells were seeded at a density of 300,000 per well supplemented DMEM without antibiotics. On day 2, cells were transfected following the manufacturer's instructions, with a ratio of 0.5 µg DNA to 1.5 µL transfection TransIT-2020. On day 4 (48 hours after transfection), cells were washed with PBS, trypsinized, resuspended in FACS buffer and stained with DAPI (1 µg/mL) prior to flow cytometry on a Attune NxT instrument (ThermoFisher Scientific). The data was analyzed with FlowJo v10. Events were gated based on fluorescence of cells stably expressing GFP-BCL-x_L and transfected with an mCherry-only control (quadrant Q2). Degradation of BCL-x_L was indicated by reduced GFP fluorescence intensity and a shift to Q1. Results represent at least three independent experiments and are expressed as the percentage of degraded GFP-BCL-x_L (100 x Q1/(Q1 + Q2) ± standard deviation). Statistics were calculated using a two-sided Welsch's T-test. Significance levels are represented as N.S.: $P > 0.05$, ∗: $P \leq 0.05$, ∗∗: $P \leq 0.01$, ∗∗∗: $P \leq 0.001$, ∗∗∗∗: $P \leq 0.0001$.

Flow cytometry assay of endogenous BCL-x_L degradation

The protein constructs for the endogenous degradation assays were in the same pcDNA3.1Hygro(+) plasmid (GenScript). Constructs had N-terminal FLAG tag and were followed by mCherry preceded by a T2A self-cleaving peptide.

The flow cytometry assay was performed as previously described (see https://www.biorxiv.org/content/10.64898/2025.12.22.695764v2 and additional peer review document). A549 cells were cultured as above and fixed in 4% formaldehyde 48 hours after transfection or 48 hours after treatment with 1 μM concentration of DT2216 (#37311, Cayman Chemical). Then, following a PBS wash the cells were permeabilized with cold methanol for 15 minutes on ice and incubated overnight at 4 °C with BCL-x_L (54H6) Rabbit mAb (Alexa Fluor 488 Conjugate) antibody and a Rabbit IgG Isotype control, Alexa Fluor 488 Conjugated (Cell Signaling, BS-0295P-A488-BSS-100uL Startech Scientific Ltd, respectively). The cells were washed and resuspended in PBS for flow cytometry measurements. For the proteasome inhibitor experiments cells were treated with 20 μM carfilzomib for 24 hours prior to measurements (Carfilzomib (PR-171), #A1933, APExBIO). The data was analyzed with FlowJo v10. Results were plotted as events of BCL-x_L Alexa 488 channel. The percentage of cells that lost fluorescence was calculated by subtracting out the mCherry control using the Overton positive method. Data are from at least 3 independent experiments Statistics were calculated using a two-sided Welsch's T-test. Significance levels are represented as N.S.: $P > 0.05$, ∗: $P \leq 0.05$, ∗∗: $P \leq 0.01$, ∗∗∗: $P \leq 0.001$, ∗∗∗∗: $P \leq 0.0001$.

Flow cytometry apoptosis assay

The same DNA constructs as in the endogenous degradation assay were used. The flow cytometry apoptosis assay was performed as described above (see https://www.biorxiv.org/content/10.64898/2025.12.22.695764v2 and additional peer review document). Briefly, 48 hours before flow-cytometry measurements A549 cells were transfected with degraders or treated with DT2216 at 1 μM (#37311, Cayman Chemical). Then, cells were detached, washed with PBS and incubated for 30 minutes to allow recovery. Afterwards, the cells were stained with FITC-conjugated Annexin V (BioLegend) in accordance with the manufacturer's protocol except for a longer 45-minute incubation time. Next, the cells were washed in 1x binding buffer and resuspend in 300 μL of binding buffer containing DAPI (BD Pharmingen). Cells, undergoing early apoptosis have externalized phosphatidylserine binding to Annexin in a calcium-dependent manner. Cells in late-stage apoptosis lose membrane integrity and get stained by DAPI. Apoptosis was calculated as the sum of cells in early and late stages and expressed as percentage of total cell count.

ApoTox-Glo™ Triplex Assay

Viability, cytotoxicity and apoptosis assays were performed using the Promega kit according to the provided protocols, with the exception of additional filters to eliminate the mCherry fluorescence background; fluorescence filter wavelengths were 485 ± 10 nm for excitation and 520±20 nm for emission for the cytotoxicity measurements, and 400 ± 20 nm for excitation and

505±20 nm for emission for the viability measurements. Luminescence readings at 545±40 nm were used for the apoptosis measurements.

Supplementary Table 1. Sequences of peptides used in this study

Name	Sequences	Mass (g/mol)
Sc-apCC-2	Ac-RDRSRLARRRAALKQRIAALKQRRAALKWQIQ SDPRKK QLEQELAALDQEIAAAEQELAALDWQIAG-NH_2	7758.90
BID	Ac-EDIIRNIARHLAQVGDS[Nle]DRSIW-NH_2	2719.03
FAM-BID	FAM-[Ahx]EDIIRNIARHLAQVGDS[Nle]DRSIW-NH_2	3148.47
DAPK1	Ac–LGLPDLVAK-NH_2	966.17
FAM-DAPK1	FAM-[Ahx]LGLPDLVAK-NH_2	1395.60

Supplementary Table 2. Sequences of target protein

Name	Sequences
MCL-1 (172-327)	MGSSHHHHHHSSGLVPRGSHMSDSEVNQEAKPEVKPEVKPETHINLKVSDGSSEIFFKI KKTTPLRRLMEAFAKRQGKEMDSLRFLYDGIRIQADQTPEDLDMEDNDIIEAHREQIGG/<br>SELYRQSLEIISRYLREQATGAKDTKPMGRSGATSRKALETLRRVGDGVQRNHETAFQG MLRKLDIKNEDDVKSLSRVMIHVFSDGVTNWGRIVTLISFGAFVAKHLKTINQESCIEPLAE SITDVLVRTKRDWLVKQRGWDGFVEFFHVEDLEGG
BCL-x_L (1-26 + 81-198	MGSSHHHHHHSSGLVPRGSHMSDSEVNQEAKPEVKPEVKPETHINLKVSDGSSEIFFKI KKTTPLRRLMEAFAKRQGKEMDSLRFLYDGIRIQADQTPEDLDMEDNDIIEAHREQIGG/<br>MSQSNRELVVDFLSYKLSQKGYSWSQMAAVKQALREAGDEFELRYRRAFSDLTSQLHIT PGTAYQSFEQVVNELFRDGVNWGRIVAFFSFGGALCVESVDKEMQVLVSRIAAWMATYL NDHLEPWIQENGGWDTFVELYGNNAAAESRKGQER

KLHL20 (303-605)	MHHHHHHSSGVDLGTENLYFQ/ SMQGPRTRPRKPIRCGEVLFAVGGWCSGDAISSVERYDPQTNEWRMVASMSKRRCGV GVSVLDDLLYAVGGHDGSSYLNSVERYDPKTNQWSSDVAPTSTCRTSVGVAVLGGFLY AVGGQDGVSCLNIVERYDPKENKWTRVASMSTRRLGVAVAVLGGFLYAVGGSDGTSPL NTVERYNPQENRWHTIAPMGTRRKHLGCAVYQDMIYAVGGRDDTTELSSAERYNPRTN QWSPVVAMTSRRSGVGLAVVNGQLMAVGGFDGTTYLKTIEVFDPDANTWRLYGGMNY RRLGGGVGVIKMTHCE

Supplementary Table 3. Sequences of protein binders against MCL-1. MCL-1 binding sites are colored red

Name	Method	Sequences
Sc-apCC-2-MCL-1-1	Strategy 1	MGSSHHHHHHSSGENLYFQ/ SHMGARLAQLKQERAALEQRLAALDQEIAALEWQIQ SDPRKK QLEQKLLELQAERLRLIGDIVNLDQQILNLEAG
Sc-apCC-2-MCL-1-2	Strategy 1	MGSSHHHHHHSSGENLYFQ/ SHMGAKLLQLKDERLRLIGDLVNLDQEILNLEQQIQ SDPRKK QLEQRLAALKQERAALEQRIAALDWEIAALEAG
Sc-apCC-2-MCL-1-3	Strategy 2 (pAE)	MGSSHHHHHHSSGENLYFQ/ SHMGARLAQLKWERAALKQRLAALDQEIAALTQKIE SDPRKK ELIPRLLRLIGDRVDLERRIAALDQEIAALEAG
Sc-apCC-2-MCL-1-4	Strategy 2 (pLDDT)	MGSSHHHHHHSSGENLYFQ/ SHMGARLEELIAERLRLVGDLVDLDREIAALEQQIQ SDPRKK QLEQRLAALKQERAALEQRIAALDWEIADLDDG
Sc-apCC-2-MCL-1-5	Strategy 2 Selective (Rank pLDDT)	MGSSHHHHHHSSGENLYFQ/ SHMGARLEELITERLRLVGDLLTLDREIAALEQQIQ SDPRKK QLEQRLAALKQERAALEQRIAALDWEIEALEAG

Sc-apCC-2-MCL-1-6	Strategy 2<br>Selective<br>(Rank combined)	MGSSHHHHHHSSGENLYFQ/<br>SHMGARLAQLKQERAALKQRLAALDQEIEELEWQIQ SDPRKK<br>QLEQRLLDLIAERLRLVGDIVTLDREIAALEAG

Supplementary Table 4. Sequences of protein binders against BCL-x_L. BCL-x_L binding sites are colored blue

Name	Method	Sequences
Sc-apCC-2-BCL-x_L-1	Strategy 1<br>(pAE)	MGSSHHHHHHSSGENLYFQ/<br>SHMGARLAQLKQERAALKQRLAALDQEIAALEWQIQ SDPRKK<br>QLYQRLLALISDRFALEQRIAALDQEIAALEAG
Sc-apCC-2-BCL-x_L-2	Strategy 1<br>(pLDDT)	MGSSHHHHHHSSGENLYFQ/<br>SHMGARLAQLKQERAALKQRLAALDQEIAALEWQIQ SDPRKK<br>QLIQRLLALISDRFALEQRIAALDQEIAALEAG
Sc-apCC-2-BCL-x_L-3	Strategy 2<br>(pAE)	MGSSHHHHHHSSGENLYFQ/<br>SHMGARLAQLKQERAALKQRLEALDQEIAALEWQIQ SDPRKK<br>QLQQRLRELIGERLRLFGDIFQLDVEIAALEAG

Sc-apCC-2-BCL-x_L-4	Strategy 2 (pAE)	MGSSHHHHHHSSGENLYFQ/ SHMGARLAQLKQERAALKQRLDALDQEIAALEWQIQ SDPRKK QLYQRLVELIGERLRLISDIFELDVEIAALEAG
Sc-apCC-2-BCL-x_L-5	Strategy 2 (pLDDT)	MGSSHHHHHHSSGENLYFQ/ SHMGARLAQLKQERAALKQRLDALDQEIAALEWQIQ SDPRKK QLYQRLLELIGERLALMGDIFELDVEIAALEAG
Sc-apCC-2-BCL-x_L-6	Strategy 2 (pLDDT)	MGSSHHHHHHSSGENLYFQ/ SHMGARLAQLKQERAALKQRLEALDQEIAALEWQIQ SDPRKK QLIQRLRELFGERLSLMGDIFQLDVEIAALEAG
Sc-apCC-2-BCL-x_L-7	Strategy 2 (ΔG)	MGSSHHHHHHSSGENLYFQ/ SHMGARLAQLKQERAALKQRLAALDQEIEALEWQIQ SDPRKK QLFGRLLSLFGDRFDLERRIAALDQEIAALEAG
Sc-apCC-2-BCL-x_L-8	Strategy 2 (ΔG)	MGSSHHHHHHSSGENLYFQ/ SHMGARLAQLKQERAALKQRLEALDQEIAALEWQIQ SDPRKK QLLQRLYELFGERLALFGDIFDLDVEIAALEAG
Sc-apCC-2-BCL-x_L-9	Strategy 2 (Sc)	MGSSHHHHHHSSGENLYFQ/ SHMGARLAQLKQERAALKQRLAALDQEIAALEWQIQ SDPRKK QLFGRLLGLMGDRFELEQRIAALDQEIAALEAG
Sc-apCC-2-BCL-x_L-10	Strategy 2 (Sc)	MGSSHHHHHHSSGENLYFQ/ SHMGARLAQLKQERAALKQRLAALDQEIEALEWQIQ SDPRKK QLFGRLLSLFSDRFDLERRIAALDQEIAALEAG
Sc-apCC-2-BCL-x_L-11	Strategy 3 (pAE)	MGSSHHHHHHSSGENLYFQ/ SHMGPELAALQAERDALEARLAELDREIAALEWRIP SDPRKK QLIGELLRLFGDRFDLERRIAELDAKIEALKNG
Sc-apCC-2-BCL-x_L-12	Strategy 3 (pLDDT)	MGSSHHHHHHSSGENLYFQ/ SHMGPELERLEERRRELERELEELDREIAELEWTIK SDPRKK

		ELIVKLRQLFGRRLRLMGDIFDLDVQIRRLREG
Sc-apCC-2-BCL-x_L-13	Strategy 4 (pAE)	MGSSHHHHHHSSGENLYFQ/ SHMGARLAQLKQERAALKQRLAALDQEIAALEWQIQ SDPRKV QLWGRLLGLLADRFELEQRIAALDQEIAALEAG
Sc-apCC-2-BCL-x_L-14	Strategy 4 (pLDDT)	MGSSHHHHHHSSGENLYFQ/ SHMGARLAQLKQERAALKQRLDALEQEIAALEWQIQ SDPRKK QLQQRLRQLYGERLRLESDIFDLDVEIAALEAG
Sc-apCC-2-BCL-x_L-15	Strategy 2 Selective (rank pLDDT)	MGSSHHHHHHSSGENLYFQ/ SHMGARLAQLKQERAALKQRLEALDQEIAALEWQIQ SDPRKK QLQQRLRELIGERLRLMGDIFQLDVEIAALEAG
Sc-apCC-2-BCL-x_L-16	Strategy 2 Selective (rank pAE)	MGSSHHHHHHSSGENLYFQ/ SHMGARLAQLKQERAALKQRLDALDQEIAALEWQIQ SDPRKK QLLQRLRELIGERLRLISDIFDLDVEIAALEAG
Sc-apCC-2-BCL-x_L-17	Strategy 2 Selective (rank Sc)	MGSSHHHHHHSSGENLYFQ/ SHMGARLAQLKQERAALKQRLAALDQEIAALEWQIQ SDPRKK QLLERLRRLFGERLRLFSDIFQLDQEIAALEAG
Sc-apCC-2-BCL-x_L-18	Strategy 2 Selective (rank combined)	MGSSHHHHHHSSGENLYFQ/ SHMGARLAQLKQERAALKQRLEALDQEIAALEWQIQ SDPRKK QLLQRLRRLYGERLALFGDIFDLDVEIAALEAG

Supplementary Table 6. Sequences of protein binders against KLHL20. KLHL20 binding sites are colored yellow

Name	Method	Sequences
Sc-apCC-2-KLHL20-1	Short loop natural	MGSSHHHHHHSSGENLYFQ/ SHMGARLAQLKQERAALKQRLAALDQEIAALEWQIQA GLPDLVG AQLEQRLAALKQERAALEQRIAALDQEIAALEAG
Sc-apCC-2-KLHL20-2	Long loop natural	MGSSHHHHHHSSGENLYFQ/ SHMGARLAQLKQERAALKQRLAALDQEIAALEWQIQA GLGLPDLVAKYNG AQLEQRLAALKQERAALEQRIAALDQEIAALEAG
Sc-apCC-2-KLHL20-3	Short loop MPNN	MGSSHHHHHHSSGENLYFQ/ SHMGARLAQLKQERAALKQRLAALDQEIAALQARIDA GAPDLVR PVLEARLAALKQERAALEQRIAALDWEIAALEAG
Sc-apCC-2-KLHL20-4	Long loop MPNN	MGSSHHHHHHSSGENLYFQ/ SHMGARLAQLKQERAALKQRLAALDQEIAALEWQIQ QAKTLPDLVREVV VSQLEQRLAALKQERAALEQRIAALDQEIAALEAG

Supplementary Table 7. Sequences of bifunctional protein binders. BCL-x_L binding sites are colored blue, KLHL20 binding sites are colored yellow

Name	Sequences
Sc-apCC-2-BCL-x_L-5 - KLHL20-3	MGSSHHHHHHSSGENLYFQ/ SHMGARLAQLKWERAALKQRLAALDQEIAALQARIDA GAPDLVR PVLYQRLLELIGERLALMGDIFELDVEIAALEAG

Supplementary Table 8. Sequences of constructs used in GFP-assay see Figure 12. Linkers between domains in lower case, MCL-1 binding sites are colored red. BCL-x_L binding sites are colored blue, KLHL20 binding sites are colored yellow

**Construct**	**Sequence**
**Construct for stable cell line generation**	
eGFP-BCL-x_L<br>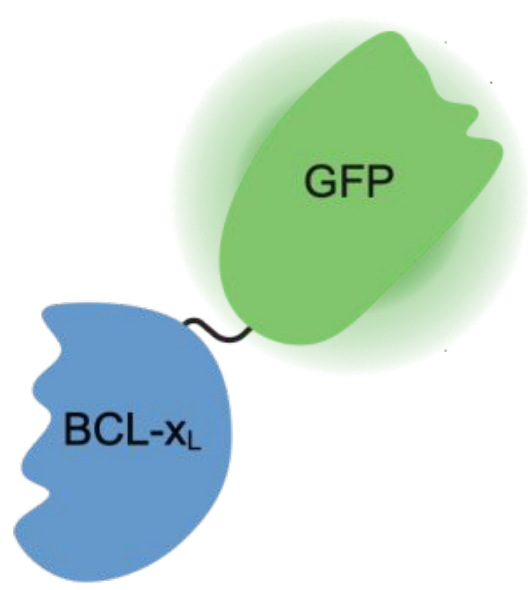 	MVSKGEELFTGVVPILVELDGDVNGHKFSVSGEGEGDATYGKLT<br>LKFICTTGKLPVPWPTLVTTLTYGVQCFSRYPDHMKQHDFFKSAM<br>PEGYVQERTIFFKDDGNYKTRAEVKFEGDTLVNRIELKGIDFKED<br>GNILGHKLEYNYNSHNVYIMADKQKNGIKVNFKIRHNIEDGSVQLA<br>DHYQQNTPIGDGPVLLPDNHYLSTQSALSKDPNEKRDHMVLLEF<br>VTAAGITLGMDELYKVDPVAGA<br><br>ggsggggs<br><br>MSQSNRELVVDFLSYKLSQKGYSWSQFSDVEENRTEAPEGTES<br>EMETPSAINGNPSWHLADSPAVNGATAHSSSLDAREVIPMAAVK<br>QALREAGDEFELRYRRAFSDLTSQLHITPGTAYQSFEQVVNELFR<br>DGVNWGRIVAFFSFGGALCVESVDKEMQVLVSRIAAWMATYLND<br>HLEPWIQENGGWDTFVELYGNNAAAESRKGQERFNRWFLTGMT<br>VAGVVLLGSLFSRK
**Full construct for BCL-x_L assay**	
3xHA-[Targeting-domain]-SPOP(167-374)-T2A-mCherry<br>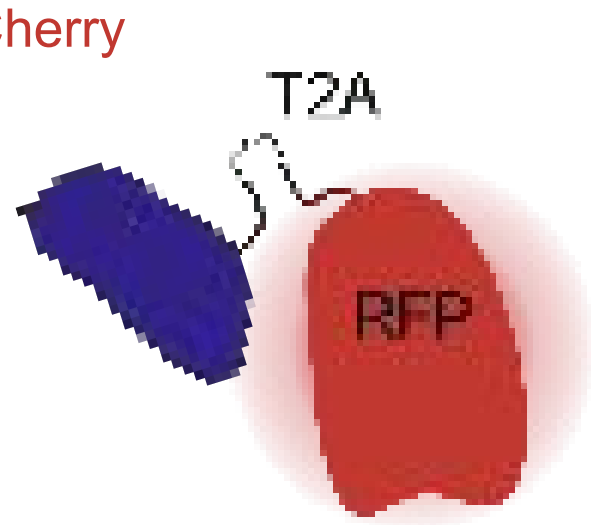 	YPYDVPDYAYPYDVPDYAYPYDVPDYAgsdq<br><br>[Targeting-domain]<br><br>gssgsg<br><br>SVNISGQNTMNMVKVPECRLADELGGLWENSRFTDCCLCVAGQ<br>EFQAHKAILAARSPVFSAMFEHEMEESKKNRVEINDVEPEVFKEM<br>MCFIYTGKAPNLDKMADDLLAAADKYALERLKVMCEDALCSNLSV

	ENAAEILILADLHSADQLKTQAVDFINYHASDVLETSGWKSMVVSH PHLVAEAYRSLASAQCPFLGPPRKRLKQS<br><br>gssggsgEGRGSLLTCGDVEENPG/Pggsg<br><br>VSKGEEDNMAIIKEFMRFKVHMEGSVNGHEFEIEGEGEGRPYEG TQTAKLKVTKGGPLPFAWDILSPQFMYGSKAYVKHPADIPDYLKL SFPEGFKWERVMNFEDGGVVTVTQDSSLQDGEFIYKVKLRGTNF PSDGPVMQKKTMGWEASSERMYPEDGALKGEIKQRLKLKDGGH YDAEVKTTYKAKKPVQLPGAYNVNIKLDITSHNEDYTIVEQYERAE GRHSTGGMDELYK
**Targeting domain**	
Sc-apCC-2-MCL-1-1	…<br><br>GARLAQLKQERAALEQRLAALDQEIAALEWQIQ SDPRKK QLEQKLLELQAERLRLIGDIVNLDQQILNLEAG<br><br>…
αGFP	…<br><br>VQLVESGGALVQPGGSLRLSCAASGFPVNRYSMRWYRQAPGKE REWVAGMSSAGDRSSYEDSVKGRFTISRDDARNTVYLQMNSLK PEDTAVYYCNVNVGFEYWGQGTQVTVSS<br><br>…
Sc-apCC-2-BCL-x_L-3	…<br><br>GARLAQLKQERAALKQRLEALDQEIAALEWQIQ SDPRKK QLQQRLRELIGERLRLFGDIFQLDVEIAALEAG<br><br>…
Sc-apCC-2-BCL-x_L-4	…

	GARLAQLKQERAALKQRLDALDQEIAALEWQIQ SDPRKK QLYQRLVELIGERLRLISDIFELDVEIAALEAG<br>…
Sc-apCC-2-BCL-x_L-5	…<br>GARLAQLKQERAALKQRLDALDQEIAALEWQIQ SDPRKK QLYQRLLELIGERLALMGDIFELDVEIAALEAG<br>…
Sc-apCC-2-BCL-x_L-6	…<br>GARLAQLKQERAALKQRLEALDQEIAALEWQIQ SDPRKK QLIQRLRELFGERLSLMGDIFQLDVEIAALEAG<br>…
Sc-apCC-2-BCL-x_L-7	…<br>GARLAQLKQERAALKQRLAALDQEIEALEWQIQ SDPRKK QLFGRLLSLFGDRFDLERRIAALDQEIAALEAG<br>…
Sc-apCC-2-BCL-x_L-8	…<br>GARLAQLKQERAALKQRLEALDQEIAALEWQIQ SDPRKK QLLQRLYELFGERLALFGDIFDLDVEIAALEAG<br>…
Sc-apCC-2-BCL-x_L-9	…<br>GARLAQLKQERAALKQRLAALDQEIAALEWQIQ SDPRKK QLFGRLLGLMGDRFELEQRIAALDQEIAALEAG<br>…
Sc-apCC-2-BCL-x_L-10	…<br>GARLAQLKQERAALKQRLAALDQEIEALEWQIQ SDPRKK ›QLFGRLLSLFSDRFDLERRIAALDQEIAALEAG

	…
**Full construct for KLHL20 assay**	
3xHA-αGFP-[Degron domain]-T2A-mCherry<br>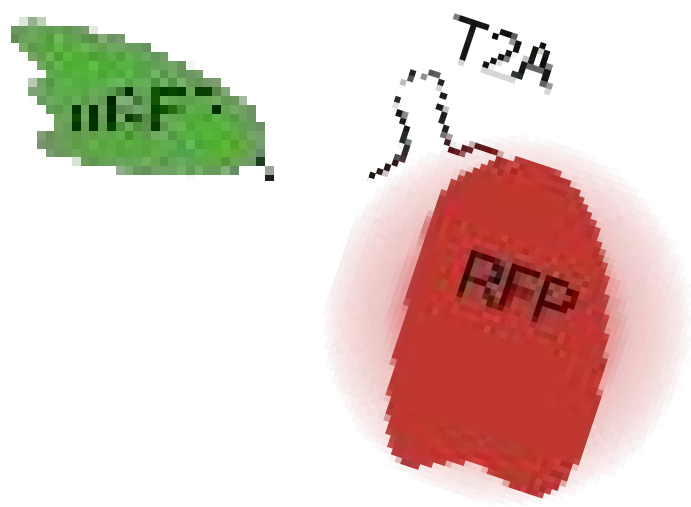 	YPYDVPDYAYPYDVPDYAYPYDVPDYAgsdq<br><br>VQLVESGGALVQPGGSLRLSCAASGFPVNRYSMRWYRQAPGKE<br>REWVAGMSSAGDRSSYEDSVKGRFTISRDDARNTVYLQMNSLK<br>PEDTAVYYCNVNVGFEYWGQGTQVTVSS<br><br>[Degron-domain]<br><br>gssggsgEGRGSLLTCGDVEENPG/Pggsg<br><br>VSKGEEDNMAIIKEFMRFKVHMEGSVNGHEFEIEGEGEGRPYEG<br>TQTAKLKVTKGGPLPFAWDILSPQFMYGSKAYVKHPADIPDYLKL<br>SFPEGFKWERVMNFEDGGVVTVTQDSSLQDGEFIYKVKLRGTNF<br>PSDGPVMQKKTMGWEASSERMYPEDGALKGEIKQRLKLKDGGH<br>YDAEVKTTYKAKKPVQLPGAYNVNIKLDITSHNEDYTIVEQYERAE<br>GRHSTGGMDELYK
**Degron domain**	
KLHL20d	…<br><br>LLAMNLGLPDLVAKYNTSNGAgsgsLLAMNLGLPDLVAKYNTSNG<br>AgsgsLLAMNLGLPDLVAKYNTSNGA<br><br>…
Sc-apCC-2-KLHL20-1	…<br><br>GARLAQLKQERAALKQRLAALDQEIAALEWQIQA GLPDLVG<br>AQLEQRLAALKQERAALEQRIAALDQEIAALEAG

	…
Sc-apCC-2-KLHL20-2	…<br>GARLAQLKQERAALKQRLAALDQEIAALEWQIQA<br>GLGLPDLVAKYNG<br>AQLEQRLAALKQERAALEQRIAALDQEIAALEAG<br>…
Sc-apCC-2-KLHL20-3	…<br>GARLAQLKQERAALKQRLAALDQEIAALQARIDA GAPDLVR<br>PVLEARLAALKQERAALEQRIAALDWEIAALEAG<br>…
Sc-apCC-2-KLHL20-4	…<br>GARLAQLKQERAALKQRLAALDQEIAALEWQIQ<br>QAKTLPDLVREVV<br>VSQLEQRLAALKQERAALEQRIAALDQEIAALEAG<br>…

Supplementary Table 9. Sequences of constructs used in endogenous degradation and apoptosis assay Linkers between domains in lower case, BCL-x_L binding sites are colored blue, KLHL20 binding sites are colored yellow

Construct	Sequence
**Full construct for endogenous degradation**	
3xHA-[Degradation domain]-T2A-mCherry<br>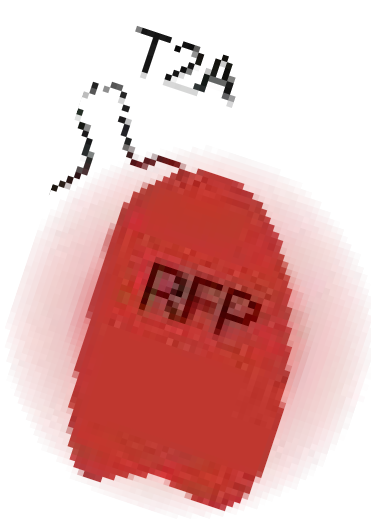 	YPYDVPDYAYPYDVPDYAYPYDVPDYAgsdq<br><br>[Degradation domain]<br><br>gssggsgEGRGSLLTCGDVEENPG/Pggsg<br><br>VSKGEEDNMAIIKEFMRFKVHMEGSVNGHEFEIEGEGEGRPYEG<br>TQTAKLKVTKGGPLPFAWDILSPQFMYGSKAYVKHPADIPDYLKL<br>SFPEGFKWERVMNFEDGGVVTVTQDSSLQDGEFIYKVKLRGTN<br>FPSDGPVMQKKTMGWEASSERMYPEDGALKGEIKQRLKLKDGG<br>HYDAEVKTTYKAKKPVQLPGAYNVNIKLDITSHNEDYTIVEQYER<br>AEGRHSTGGMDELYK
**Degradation domain**	
Sc-apCC-2-BCLx_L-5	…<br><br>GARLAQLKQERAALKQRLDALDQEIAALEWQIQSDPRKKQLYQR<br>LLELIGERLALMGDIFELDVEIAALEAG<br><br>…
Sc-apCC-2-KLHL20-3	…

	GARLAQLKQERAALKQRLAALDQEIAALQARIDAGAPDLVRPVLE ARLAALKQERAALEQRIAALDWEIAALEAG<br>…
Sc-apCC-2-BCLx$_L$-5-KLHL20-3	…<br>GARLAQLKWERAALKQRLAALDQEIAALQARIDAGAPDLVRPVLY QRLLELIGERLALMGDIFELDVEIAALEAG<br>…
Sc-apCC-2-BCLx$_L$-5_Sc-apCC-2-KLHL20-3	…<br>GARLAQLKQERAALKQRLDALDQEIAALEQQIQSDPRKKQLYQR LLELIGERLALMGDIFELDVEIAALEAGGGSGGSGARLAQLKQER AALKQRLAALDQEIAALQARIDAGAPDLVRPVLEARLAALKQERA ALEQRIAALDWEIAALEAG<br>…
Sc-apCC-2-KLHL20-4	…<br>GARLAQLKQERAALKQRLAALDQEIAALEWQIQQAKTLPDLVREV VVSQLEQRLAALKQERAALEQRIAALDQEIAALEAG<br>…
Sc-apCC-2-BCLx$_L$-9-KLHL20-4	…<br>GARLAQLKWERAALKQRLAALDQEIAALEQQIQQAKTLPDLVREV VVSQLFGRLLGLMGDRFELEQRIAALDQEIAALEAG<br>…
Sc-apCC-2-BCLx$_L$-9_Sc-apCC-2-KLHL20-3	…<br>GARLAQLKQERAALKQRLAALDQEIAALEQQIQSDPRKKQLFGRL LGLMGDRFELEQRIAALDQEIAALEAGGGSGGSGARLAQLKQER AALKQRLAALDQEIAALQARIDAGAPDLVRPVLEARLAALKQERA ALEQRIAALDWEIAALEAG…

Sc-apCC-2-BCLx_L-10-KLHL20-4	…<br><br>GARLAQLKQERAALKQRLAALDQEIAALEQQIQQAKTLPDLVREVVVSQLFGRLLSLFSDRFDLERRIAALDQEIAALEAG<br><br>…
Sc-apCC-2-BCLx_L-10_Sc-apCC-2-KLHL20-3	…<br><br>GARLAQLKQERAALKQRLAALDQEIEALEQQIQSDPRKKQLFGRLLSLFSDRFDLERRIAALDQEIAALEAGGGSGGSGARLAQLKQERAALKQRLAALDQEIAALQARIDAGAPDLVRPVLEARLAALKQERAALEQRIAALDWEIAALEAG<br><br>…

Supplementary Table 10. Sequence motifs used in BCL-2 family designs

**Name**	**Motif Sequences**		
BCL-x_L	Y...L..IGD.F	I...L..IGD.F	F...L..IGD.F
	Y...L..FGD.F	I...L..FGD.F	F...L..FGD.F
	Y...L..ISD.F	I...L..ISD.F<br>(BIM)	F...L..ISD.F
	Y...L..FSD.F	I...L..FSD.F	F...L..FSD.F
	Y...L..MSD.F<br>(BAD)	I...L..MSD.F	F...L..MSD.F

	Y...L..MGD.F	I...L..MGD.F	F...L..MGD.F	
MCL-1	I...L..IGD.F	I...L..IGD.L	I...L..IGD.V (NOXA-B)	I...L..IGD.M
	I...L..VGD.F	I...L..VGD.L	I...L..VGD.V	I...L..VGD.M
	I...L..IAD.F	I...L..IAD.L	I...L..IAD.V	I...L..IAD.M
	I...L..VAD.F	I...L..VAD.L	I...L..VAD.V	I...L..VAD.M

Supplementary Table 11. Measured K_D's of used tracers in competition assays

**Name**	**Target**	**K_D (nM)**
BID-FAM	MCL-1	21.5 ± 1.1
BID-FAM	BCL-x_L	71 ± 8.4
DAPK1-FAM	KLHL20	12 200 ± 800

Supplementary Table 12. Measured IC_{50} values against the BCL-2 family targets

**Name design**	**IC_{50} BCL-x_L (nM)**	**IC_{50} MCL-1 (nM)**
Sc-apCC-2-MCL-1-1	Non-binding	230 ± 20
Sc-apCC-2-MCL-1-2	Non-binding	13 000 ± 2 000
Sc-apCC-2-MCL-1-3	40 000 ± 20 000	64 ± 2
Sc-apCC-2-MCL-1-4	20 000 ± 5 000	230 ± 30

Sc-apCC-2-MCL-1-5	100 000 ± 16 000	810 ± 70
Sc-apCC-2-MCL-1-6	100 000 ± 20 000	1 020 ± 90
Sc-apCC-2-BCL-x_L-1	Non-binding.	Non-binding
Sc-apCC-2-BCL-x_L-2	Non-binding	Non-binding
Sc-apCC-2-BCL-x_L-3	196 ± 14	660 ± 50
Sc-apCC-2-BCL-x_L-4	4 600 ± 600	18 000 ± 3 000
Sc-apCC-2-BCL-x_L-5	230 ± 20	68 ± 4
Sc-apCC-2-BCL-x_L-6	198 ± 130	640 ± 60
Sc-apCC-2-BCL-x_L-7	260 ± 20	1 150 ± 90
Sc-apCC-2-BCL-x_L-8	250 ± 7	193 ± 12
Sc-apCC-2-BCL-x_L-9	300 ± 30	3 080 ± 180
Sc-apCC-2-BCL-x_L-10	430 ± 30	26 000 ± 8 000
Sc-apCC-2-BCL-x_L-11	197 ± 10	500 ± 150
Sc-apCC-2-BCL-x_L-12	212 ± 18	270 ± 20
Sc-apCC-2-BCL-x_L-13	1 390 ± 40	9 200± 70
Sc-apCC-2-BCL-x_L-15	109 ± 14	218 ± 11
Sc-apCC-2-BCL-x_L-16	3 000 ± 400	90 000± 70 000
Sc-apCC-2-BCL-x_L-17	52 ± 3	68 000± 80 000
Sc-apCC-2-BCL-x_L-5-KLHL20-3	45 ± 2	Not applicable

Supplementary Table 13. Measured IC_{50} values against KLHL20

**Name design**	**IC_{50} KLHL20 (µM)**
Sc-apCC-2-KLHL20-1	3.9 ± 0.2
Sc-apCC-2-KLHL20-2	2.18 ± 0.16

Sc-apCC-2-KLHL20-3	3.16 ± 0.18
Sc-apCC-2-KLHL20-4	1.35 ± 0.17
Sc-apCC-2-BCL-x_L-5-KLHL20-3	2.5 ± 0.2

Supplementary Table 14. ITC fitting and thermodynamic analysis

	**Sc-apCC-2-BCL-x_L-3**
Number of binding sites	0.86
K_D	3 nM
ΔG	-12 kcal/mol
ΔH	-2.3 kcal/mol
$T\Delta S$	9.7 kcal/mol

Supplementary Table 15. Crystallization conditions

**Crystal structure**	**Crystal screen**	**Composition**

Sc-apCC-2	JCSG+ A6	0.2 M Lithium sulphate, 0.1 M Phosphate/ citrate pH 4.2 20% w/v PEG1000
Sc-apCC-2-MCL-1-1	JCSG+ D4	0.2 M Lithium sulphate, 0.1 M Sodium acetate pH 4.5, 30% w/v PEG8000
Sc-apCC-2-BCL-x_L-2	Morpheus G9	0.02 M Sodium formate, 0.02 M Ammonium, acetate; 0.2M Sodium citrate tribasic,<br><br>Dihydrate, 0.02 M Potassium sodium tartrate tetrahydrate, 0.02 M Sodium oxamate,<br><br>0.01 M Tris, 0.01M BICINE pH 8.5, 20% v/v PEG 500 MME; 10% w/v PEG 20000
Sc-apCC-2-BCL-x_L-5	Proplex G12	1.0 M Sodium citrate tribasic dihydrate, 0.1 M Sodium HEPES pH 7.0
Sc-apCC-2-BCL-x_L-6	Morpheus C5	0.03 M Sodium nitrate, 0.03 M Sodium phosphate dibasic, 0.03 M Ammonium sulphate, 0.01 M HEPES, 0.01 MOPS pH 7.5, 20% v/v PEG 500 MME; 10% w/v PEG 20000
Sc-apCC-2-BCL-x_L-8	Proplex B6	0.1 M Sodium HEPES pH 7.0, 10 % w/v PEG4000, 10 % v/v 2-Propanol

Sc-apCC-2-BCL-x_L-11	JCSG+ F10	1.1 M Sodium malonate dibasic monohydrate, 0.1 M HEPES pH 7.0, 0.5 % v/v Jeffamine® ED-2003
Sc-apCC-2-BCL-x_L-3: BCL-x_L	MPD E1	0.1 M Citric acid pH 4.0, 10% v/v MPD
Sc-apCC-2-MCL-1-3:MCL-1	Morpheus A12	0.03 M Magnesium chloride hexahydrate, 0.03 M Calcium chloride dihydrate, 0.01 M Tris, 0.01M BICINE pH 8.5, 2.5% v/v MPD; 12.5% PEG1000; 12.5% w/v PEG3350
Sc-apCC-2-MCL-1-4:MCL-1	JCSG+ B4	0.1 M HEPES pH7.5, 10% w/v PEG8000, 8% v/v ethylene glycol
Sc-apCC-2-KLHL20-3	Proplex D2	0.1 M MES pH 6.5, 10% w/v PEG5000MME, 12% v/v 1-Propanol
Sc-apCC-2-KLHL20-4	Proplex B12	0.1 M Magnesium chloride hexahydrate, 0.1 M Sodium HEPES pH 7.0, 15 % w/v PEG4000

Supplementary Table 16. Merging and refinement statistics for all X-ray structures

	Sc-apCC-2	Sc-apCC-2-MCL-1-1	Sc-apCC-2-BCL-xL-2	Sc-apCC-2-BCL-xL-5	Sc-apCC-2-BCL-xL-6
**PDB code**	9TLL	9TLM	9TLN	9TLO	9TLP
**Resolution range**	34.11 - 1.5 (1.55 - 1.5)	30.99 - 1.8 (1.86 - 1.8)	32.36 - 1.15 (1.19 - 1.15)	45.09 - 2.2 (2.28 - 2.2)	33.48 - 1.75 (1.81 - 1.75)
**Space group**	I 41	P 65 2 2	P 41 2 2	P 43 21 2	P 1
**Unit cell**	48.237 48.237 70.272 90 90 90	71.578 71.578 90.456 90 90 120	37.573 37.573 127.365 90 90 90	69.57 69.57 112.756 90 90 90	29.211 46.11 54.103 90.51 96.519 106.022
**Total reflections**	180234 (10441)	516546 (51414)	921551 (55532)	386222 (38535)	95560 (8302)
**Unique reflections**	15585 (1430)	13253 (1274)	38224 (3760)	14682 (1417)	26841 (2646)
**Multiplicity**	11.6 (7.3)	39.0 (40.4)	24.1 (14.8)	26.3 (27.2)	3.6 (3.1)
**Completeness (%)**	99.44 (97.25)	99.91 (99.69)	99.85 (99.76)	99.95 (100.00)	98.78 (96.99)
**Mean I/sigma(I)**	11.04 (1.81)	25.97 (1.62)	16.70 (0.78)	13.06 (2.22)	20.24 (10.87)
**Wilson B-factor**	13.69	35.73	16.23	37.54	14.62

**R-pim**	0.06743 (0.845)	0.0142 (0.5129)	0.01382 (0.8239)	0.03813 (0.3654)	0.02126 (0.04552)
**CC1/2**	0.955 (0.109)	1 (0.789)	1 (0.653)	0.999 (0.764)	0.999 (0.991)
**Reflections used in refinement**	12808 (1240)	13235 (1269)	33510 (3261)	14674 (1417)	26834 (2646)
**Reflections used for R-free**	617 (63)	643 (63)	1645 (162)	765 (71)	1337 (123)
**R-work**	0.1808 (0.1978)	0.2460 (0.3640)	0.1880 (0.2835)	0.2112 (0.2480)	0.1542 (0.1865)
**R-free**	0.1955 (0.2246)	0.2599 (0.3316)	0.2054 (0.3381)	0.2468 (0.2737)	0.2012 (0.2501)
**Ramachandran favored (%)**	100	100	100	98.57	99.3
**Ramachandran allowed (%)**	0	0	0	0.95	0.7
**Ramachandran outliers (%)**	0	0	0	0.48	0
**Rotamer outliers (%)**	0	1.67	1.72	0	0.43
**Clashscore**	3.45	0.83	2.45	1.76	3.15
**Average B-factor**	19.44	39.74	24.47	49.74	17.92

Supplementary Table 16 cont. Merging and refinement statistics for all X-ray structures

	Sc-apCC-2-BCL-xL-8	Sc-apCC-2-BCL-xL-11	Sc-apCC-2-KLHL20-3	Sc-apCC-2-KLHL20-4
**PDB code**	9TLQ	9TLR	9TLS	9TLT
**Resolution range**	49.15 - 2.6<br>(2.69 - 2.6)	29.4 - 1.95<br>(2.02 - 1.95)	29.37 - 1.45<br>(1.5 - 1.45)	36.74 - 1.95<br>(2.02 - 1.95)
**Space group**	P 32 2 1	I 41 2 2	P 1 21 1	P 1
**Unit cell**	113.501<br>113.501<br>86.29<br>90<br>90<br>120	58.696<br>58.696<br>83.329<br>90<br>90<br>90	49.903<br>31.91<br>75.183<br>90<br>93.523<br>90	47.834<br>53.711<br>73.656<br>74.218<br>72.058<br>65.315
**Total reflections**	410191 (41958)	128744 (7468)	286321 (23648)	90871 (9034)
**Unique reflections**	20112 (2010)	5598 (556)	42389 (4145)	45552 (4529)
**Multiplicity**	20.4 (20.9)	23.0 (13.4)	6.8 (5.7)	2.0 (2.0)

Completeness (%)	99.87 (99.80)	99.96 (100.00)	99.88 (98.97)	99.90 (100.00)
Mean I/sigma(I)	15.49 (2.05)	15.67 (2.25)	12.22 (1.95)	18.28 (7.22)
Wilson B-factor	62.89	31.36	18.34	16.16
R-pim	0.02497 (0.2766)	0.02474 (0.3094)	0.02967 (0.3854)	0.02586 (0.06315)
CC1/2	0.999 (0.903)	0.999 (0.809)	0.999 (0.835)	0.998 (0.986)
Reflections used in refinement	20086 (2006)	5596 (556)	42379 (4143)	45499 (4528)
Reflections used for R-free	939 (81)	281 (25)	2124 (214)	2197 (221)
R-work	0.2439 (0.3053)	0.2207 (0.2623)	0.1804 (0.2437)	0.1755 (0.1909)
R-free	0.2758 (0.3861)	0.2455 (0.2930)	0.2104 (0.2946)	0.2226 (0.2584)
Ramachandran favored (%)	96.68	95.71	99.55	98.59
Ramachandran allowed (%)	3.32	4.29	0.45	1.41
Ramachandran outliers (%)	0	0	0	0
Rotamer outliers (%)	0.64	0	0	0.8
Clashscore	2.97	2.76	3.77	2.27
Average B-factor	81.3	38.31	25.85	18.45

Supplementary Table 16 cont. Merging and refinement statistics for all X-ray structures

	**BCL-xL:Sc-apCC-2-BCL-xL-3**	**MCL-1:Sc-apCC-2-MCL-1-3**	**MCL-1:Sc-apCC-2-MCL-1-4**
PDB code	9TLU	9TLV	9TLW
Resolution range	73.99 - 2.99<br>(3.42 - 2.99)	57.52 - 2.0<br>(2.07 - 2.0)	58.34 - 2.8<br>(2.95 - 2.8)
Space group	P 61	P 1 21 1	P 1 21 1
Unit cell	85.441<br>85.441<br>145.517<br>90<br>90<br>120	62.194<br>57.952<br>63.079<br>90<br>114.239<br>90	50.353<br>83.881<br>63.379<br>90<br>113.005<br>90
Total reflections	410169 (54651)	379065 (35888)	85880 (12885)
Unique reflections	20106 (2546)	27768 (2749)	12058 (1743)

Multiplicity	20.4 (21.5)	13.7 (13.1)	7.1 (7.4)
Completeness (%)	99.91 (99.95)	99.31 (98.06)	100.0 (100.0)
Mean I/sigma(I)	15.49 (5.23)	18.90 (9.42)	15.9 (8.4)
Wilson B-factor	77.44	23.14	44.68
R-pim	0.02495 (0.1131)	0.02171 (0.08759)	0.027 (0.058)
CC1/2	0.999 (0.975)	0.999 (0.961)	0.998 (0.996)
Reflections used in refinement	12190 (4059)	27658 (2729)	12024 (1196)
Reflections used for R-free	544 (184)	1323 (138)	572 (53)
R-work	0.2747 (0.3626)	0.2081 (0.2362)	0.2483 (0.2988)
R-free	0.3216 (0.4027)	0.2559 (0.2718)	0.2948 (0.3665)
Ramachandran favored (%)	94.12	98.13	98.68
Ramachandran allowed (%)	5.39	1.87	1.32
Ramachandran outliers (%)	0.49	0	0
Rotamer outliers (%)	0.93	0.31	2.23
Clash score	13.38	4.44	9.47
Average B-factor	79.19	30.62	60.82

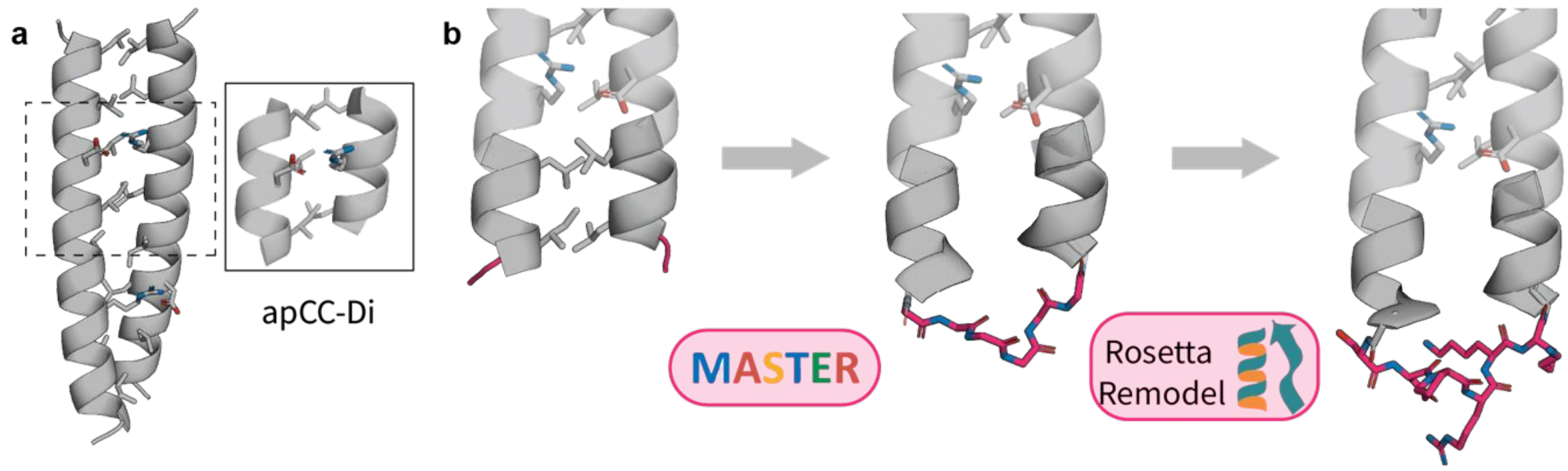

Supplementary Figure 1. Schematic of loop design a, Structure of the apCC-Di (PDB: 7Q1R) peptide. The zoom-in shows the key aspartate-arginine pair. **b,** Schematic of the loop design. First, the final 7 residues of each helix are used as MASTER search motif. MASTER was used to identify different gap length in between the two helices, ranging from 3 to 6. As 4 residue loops were the most common, the top hit was used. The sequence of this loop was subsequently redesigned with Rosetta Remodel.

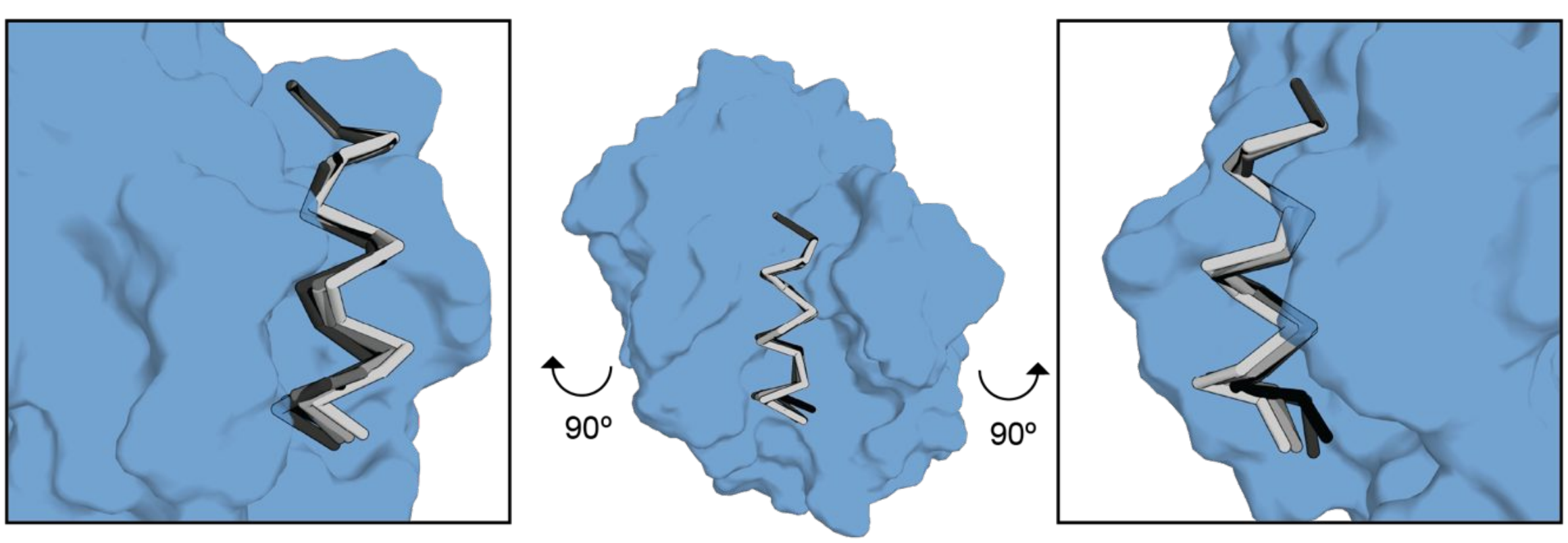

Supplementary Figure 2. Different models for binder alignment. Models of BCL-x_L (blue) in complex with a peptide effector. The position of the effector (black to light grey) was varied slightly with a Rosetta script to generate different starting poses

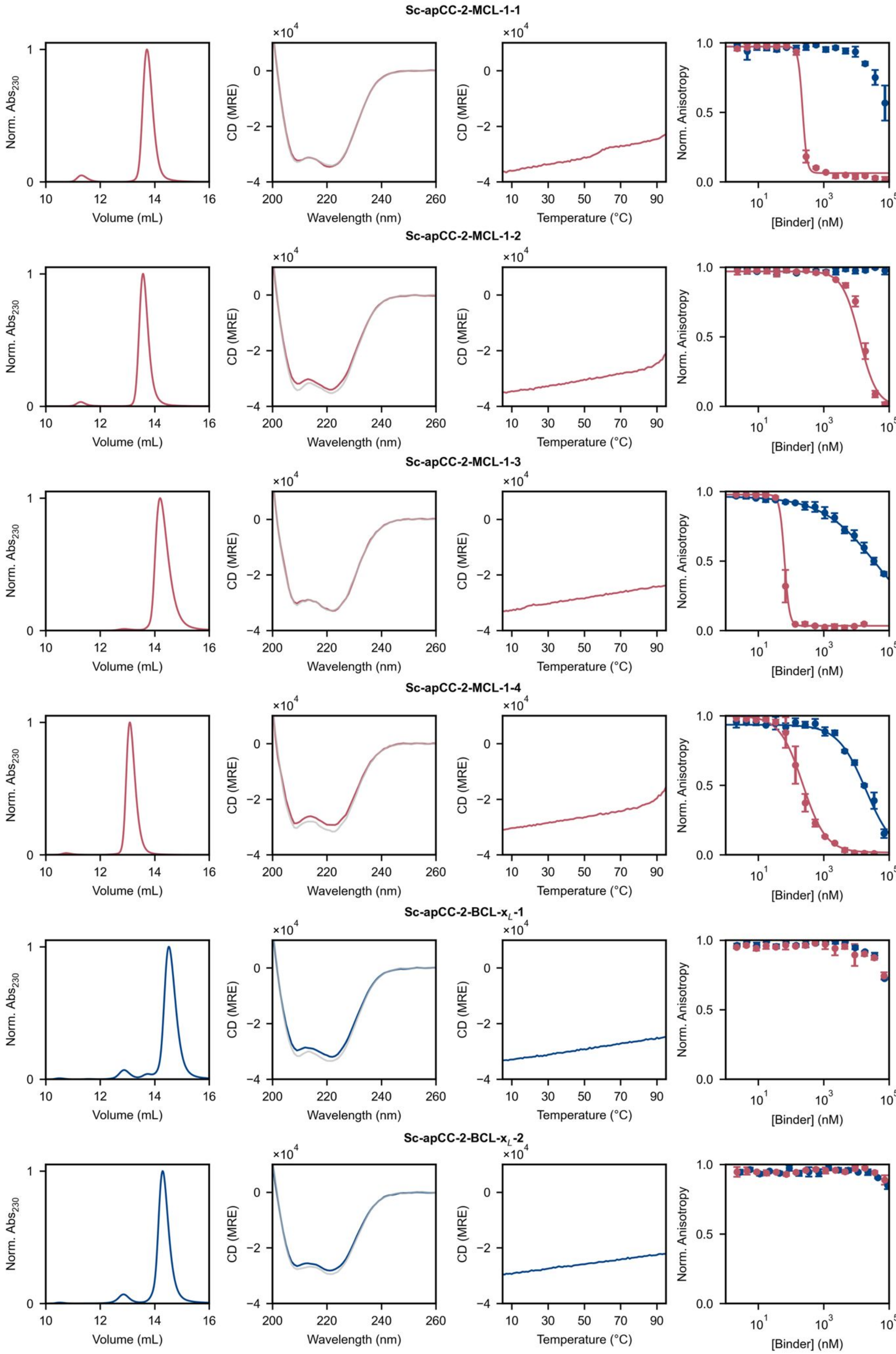
Sc-apCC-2-MCL-1-1
Sc-apCC-2-MCL-1-2
Sc-apCC-2-MCL-1-3
Sc-apCC-2-MCL-1-4
Sc-apCC-2-BCL-x$_L$-1
Sc-apCC-2-BCL-x$_L$-2
Norm. Abs$_{230}$
Volume (mL)
CD (MRE)
×10^4
Wavelength (nm)
Temperature (°C)
Norm. Anisotropy
[Binder] (nM)

Sc-apCC-2-BCL-x_L-3

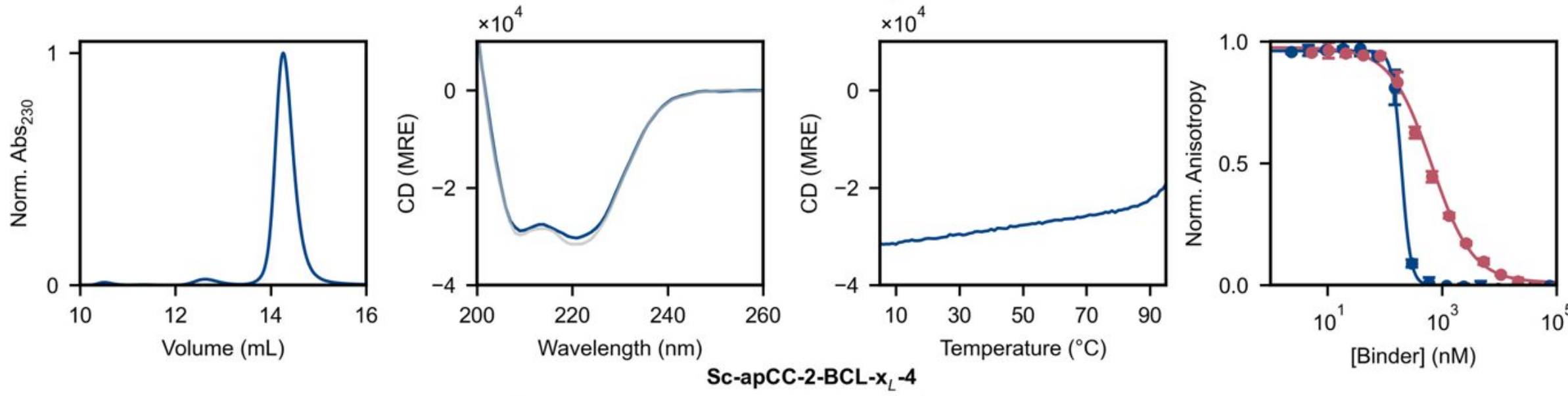

Sc-apCC-2-BCL-x_L-4

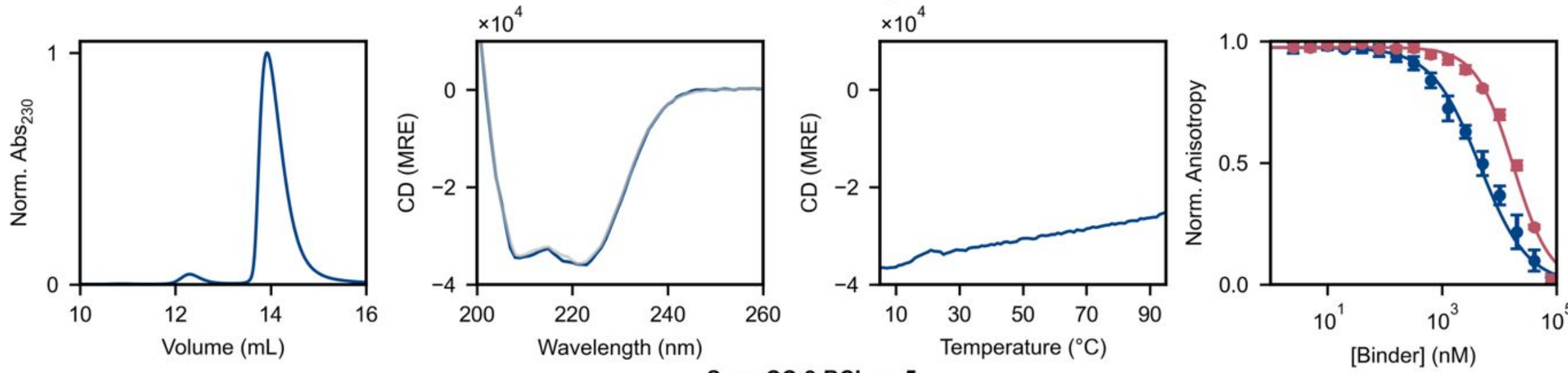

Sc-apCC-2-BCL-x_L-5

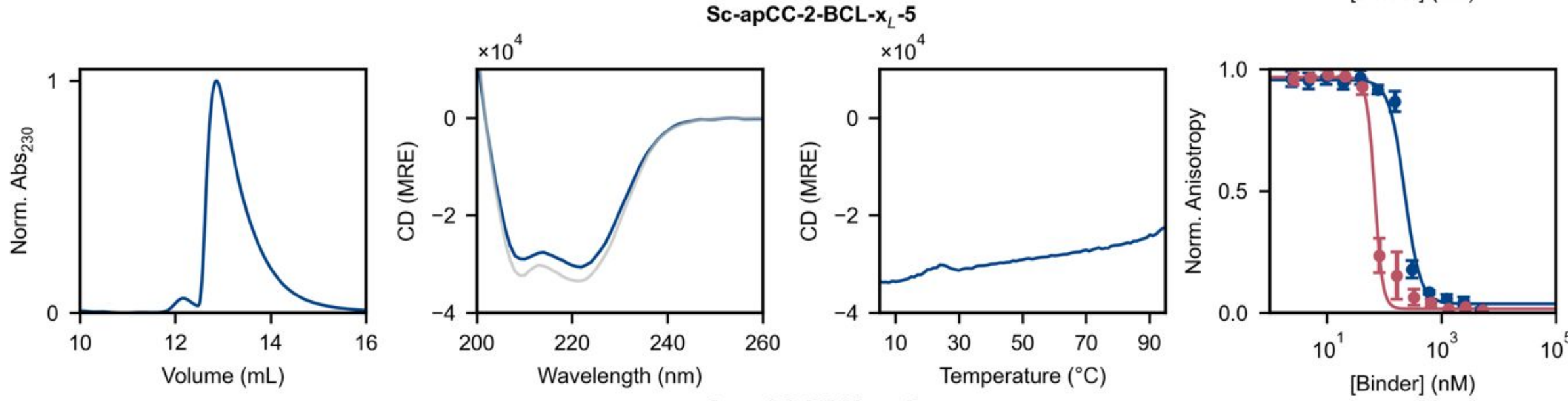

Sc-apCC-2-BCL-x_L-6

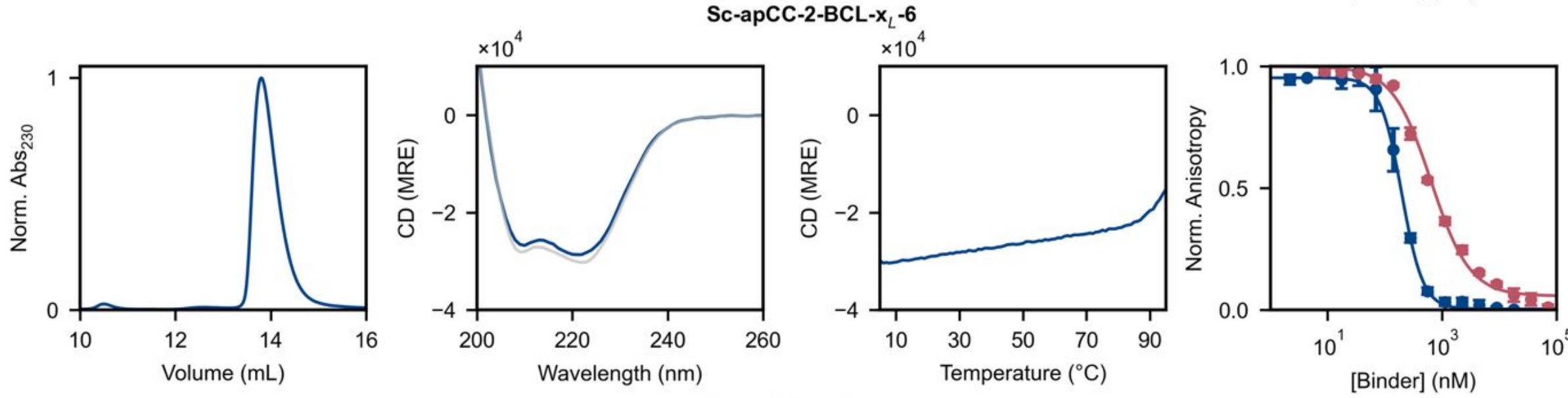

Sc-apCC-2-BCL-x_L-7

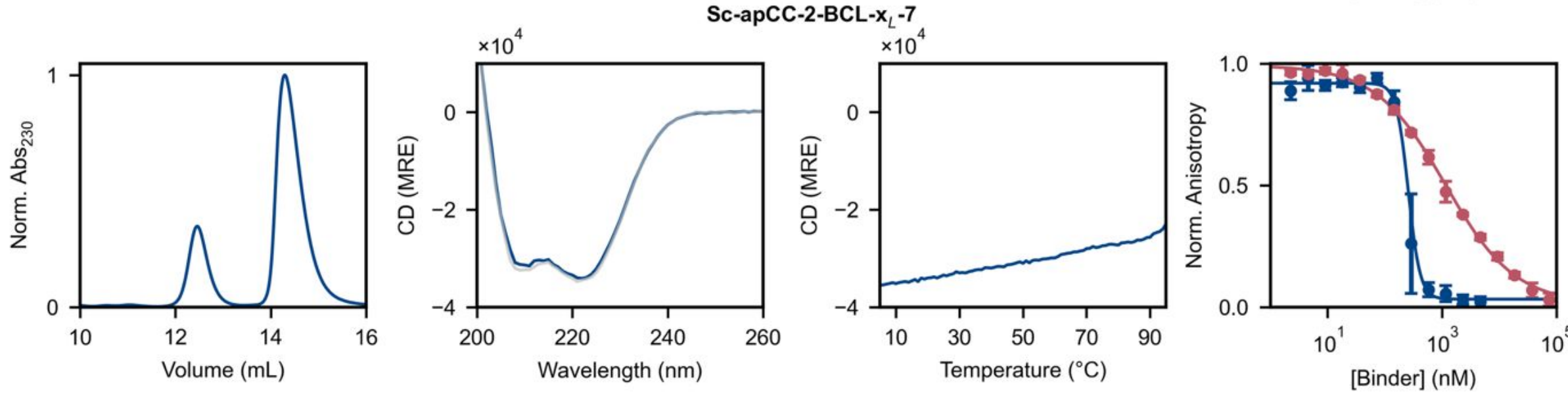

Sc-apCC-2-BCL-x_L-8

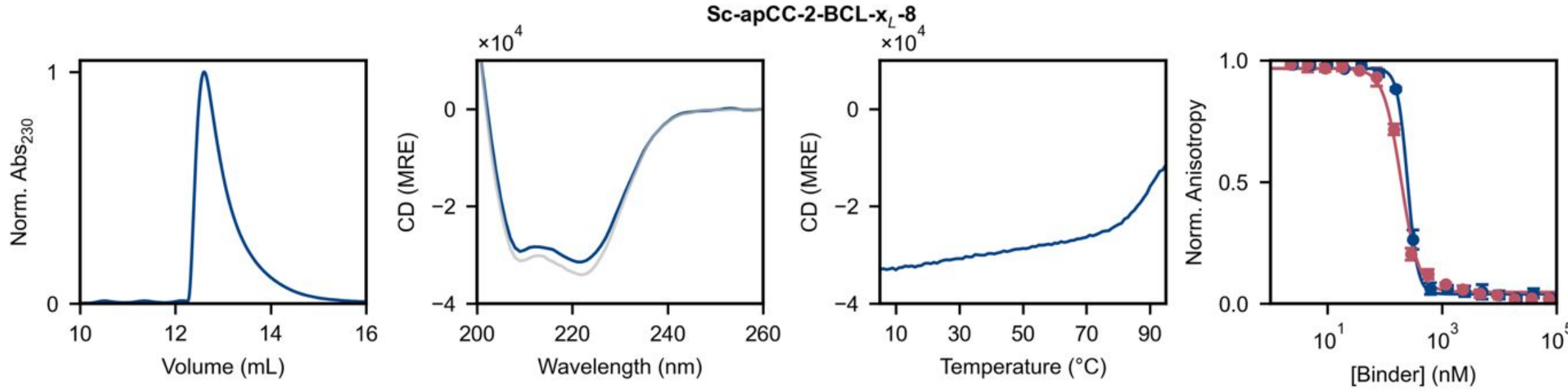

Sc-apCC-2-BCL-x$_L$-9

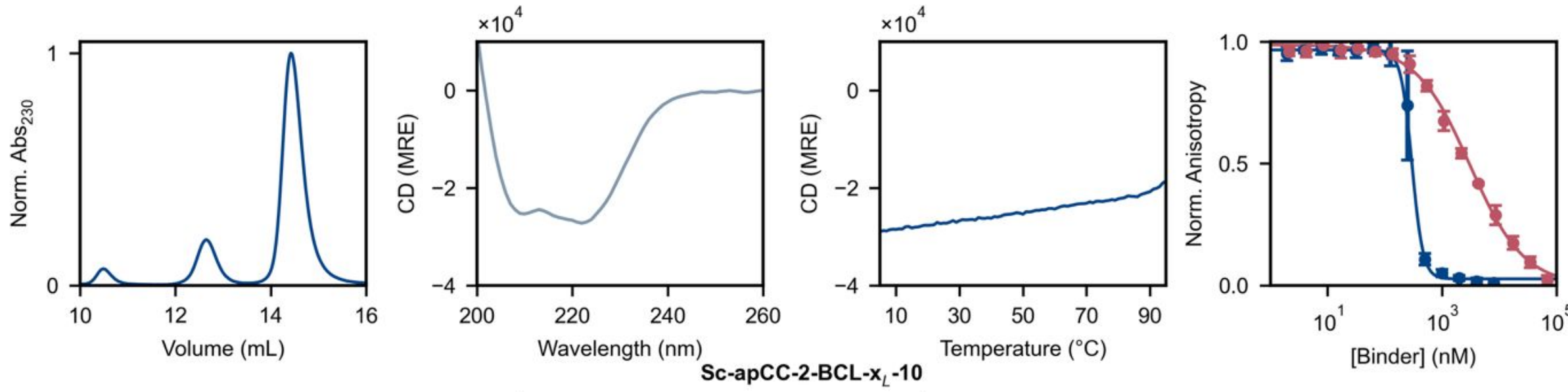

Sc-apCC-2-BCL-x$_L$-10

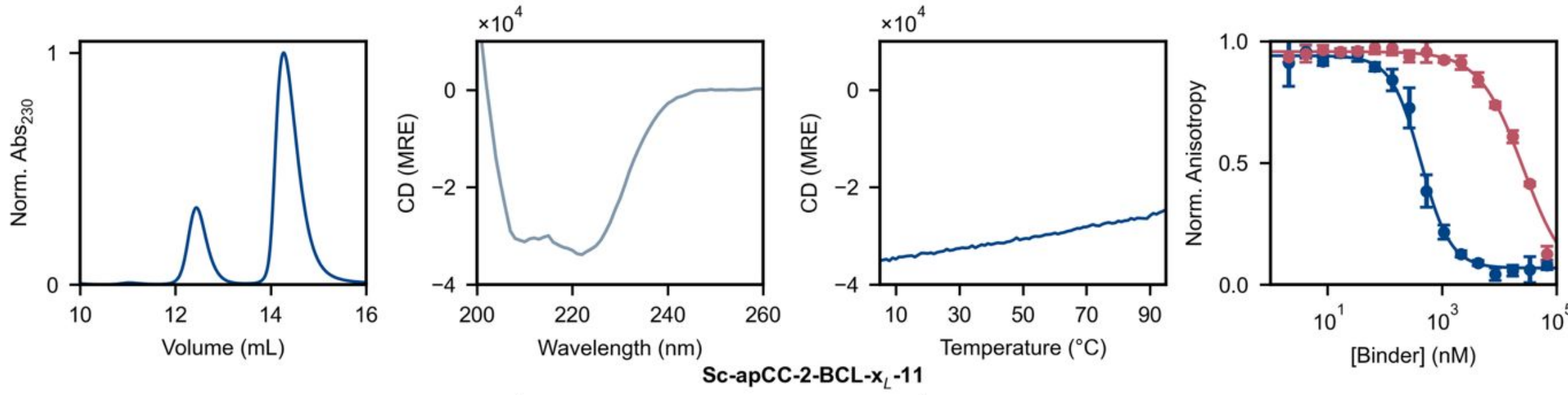

Sc-apCC-2-BCL-x$_L$-11

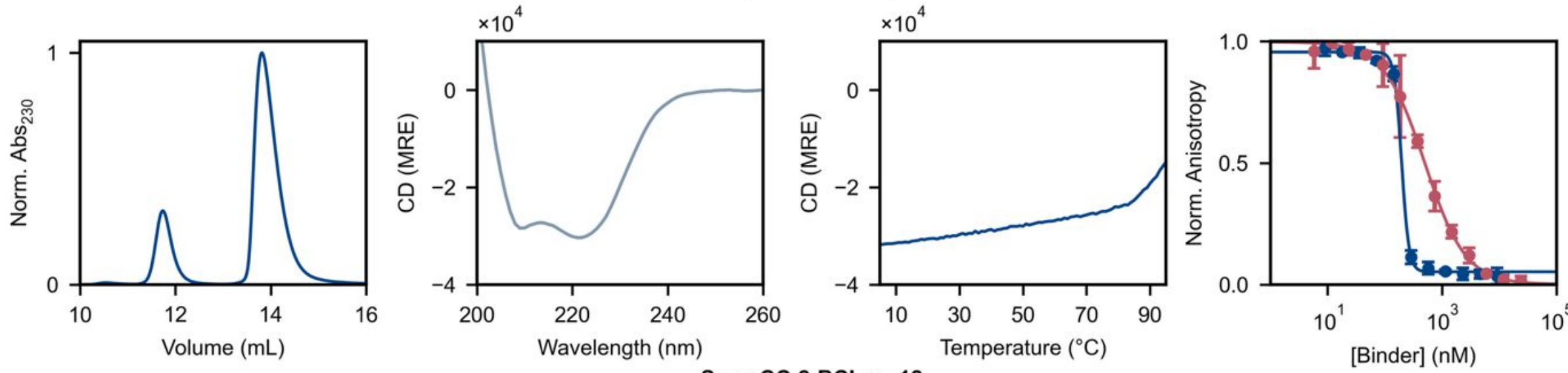

Sc-apCC-2-BCL-x$_L$-12

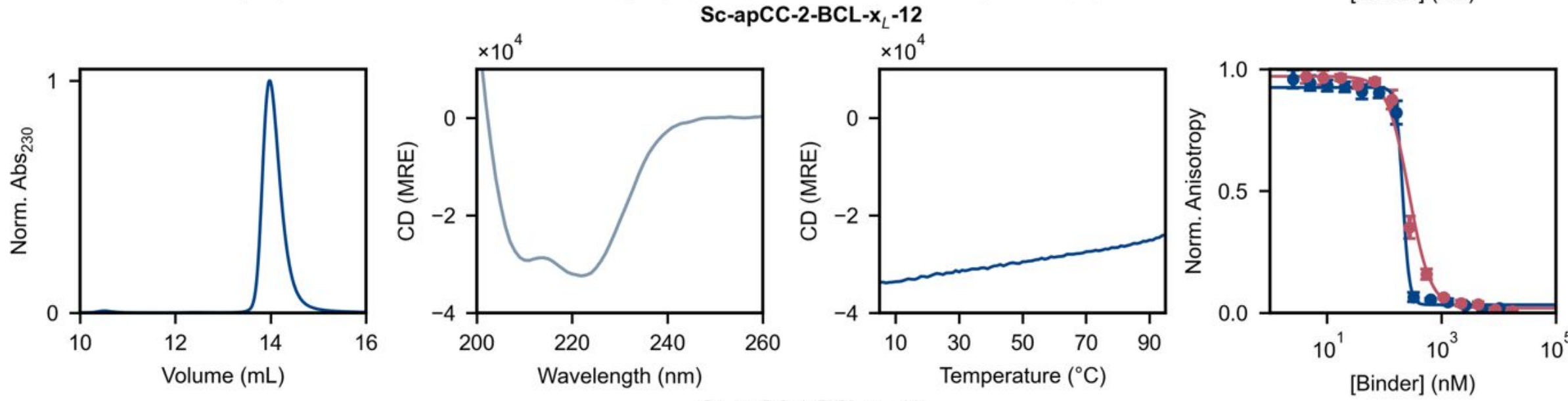

Sc-apCC-2-BCL-x$_L$-13

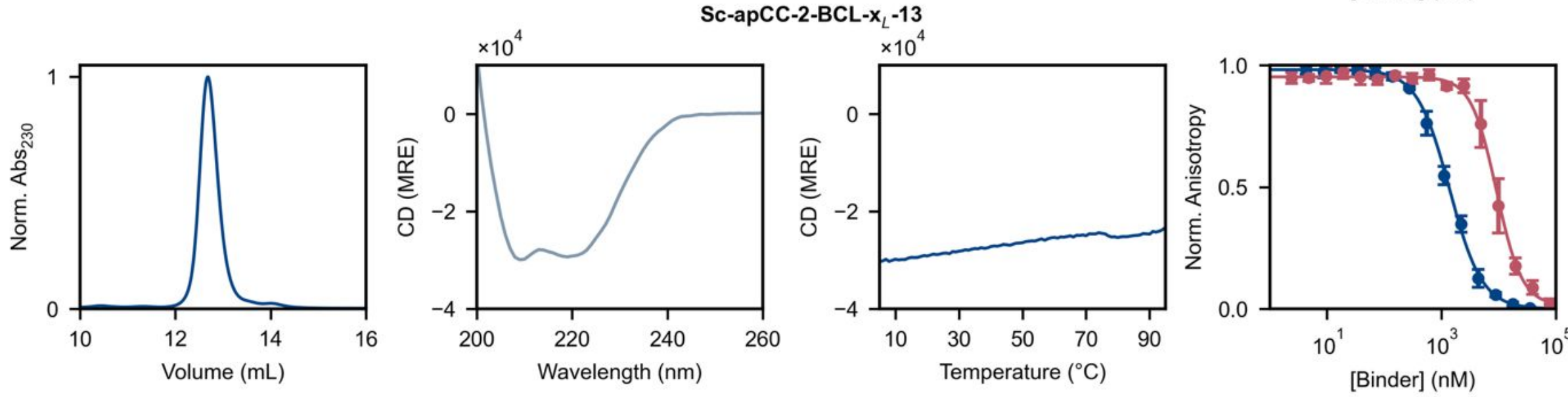

Supplementary Figure 3. Biophysics and fluorescent anisotropy data of the first round of designs. From left to right analytical size exclusion chromatography Superdex 75 increase, signal is shown as normalized UV absorbance at 230 nm; protein concentration 100 µM in 20 mM Tris-HCl, 150 mM NaCl, pH 7.5. CD spectrum; CD thermal melt; protein concentration 20 µM in 20 mM Tris-HCl, 150 mM NaCl, pH 7.5 FA assay against BCL-x_L (dark blue) and MCL-1 (red) in 20 mM Tris-HCl, 150 mM NaCl, pH 7.5.

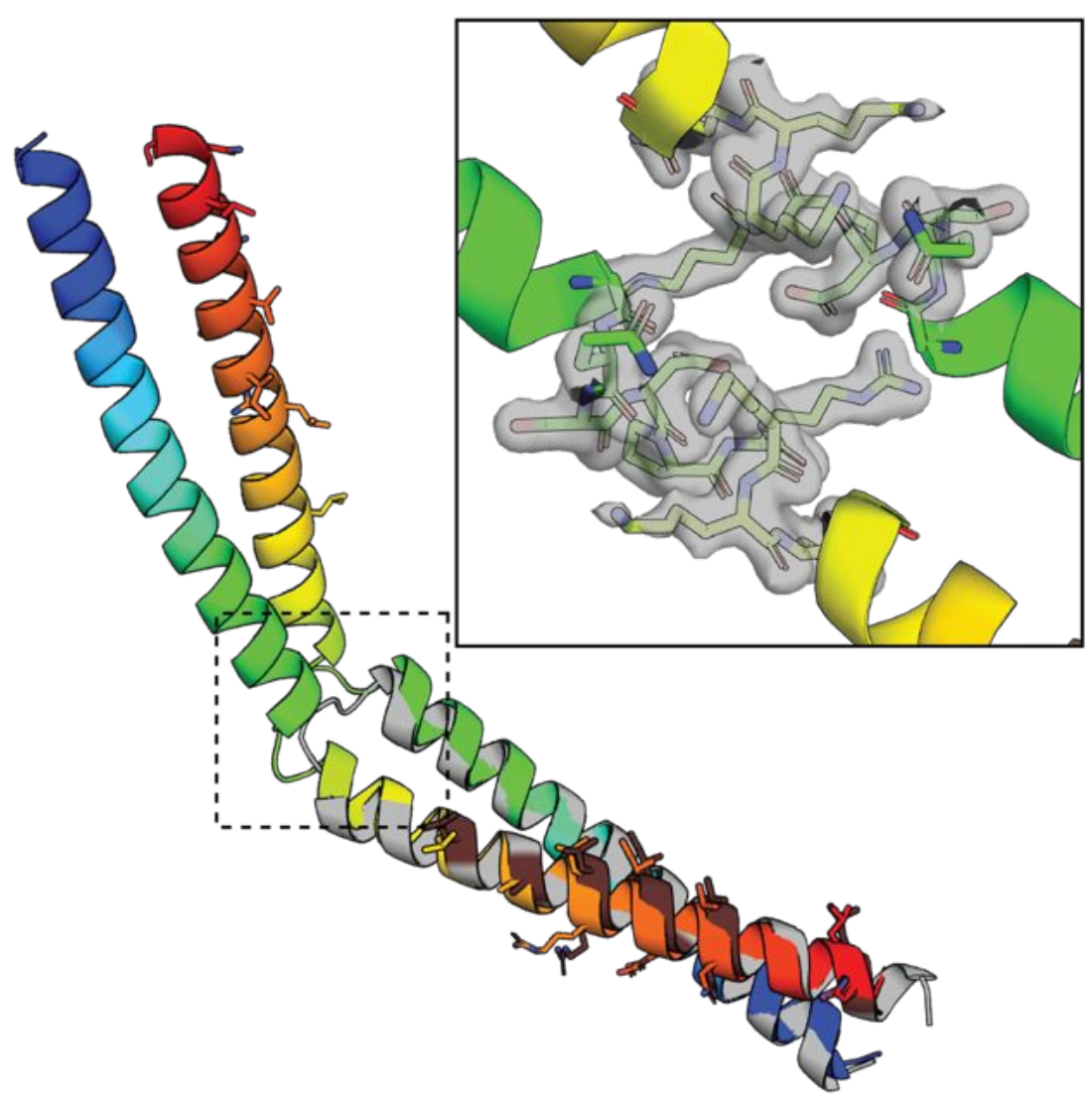

Supplementary Figure 4. Crystal structure of Sc-apCC-2-MCL-1-1. This crystal structure showed a domain-swapped dimer structure as opposed to the designed hairpin. Zoom-in shows electron density at sigma level 1 (PDB id 9TLM).

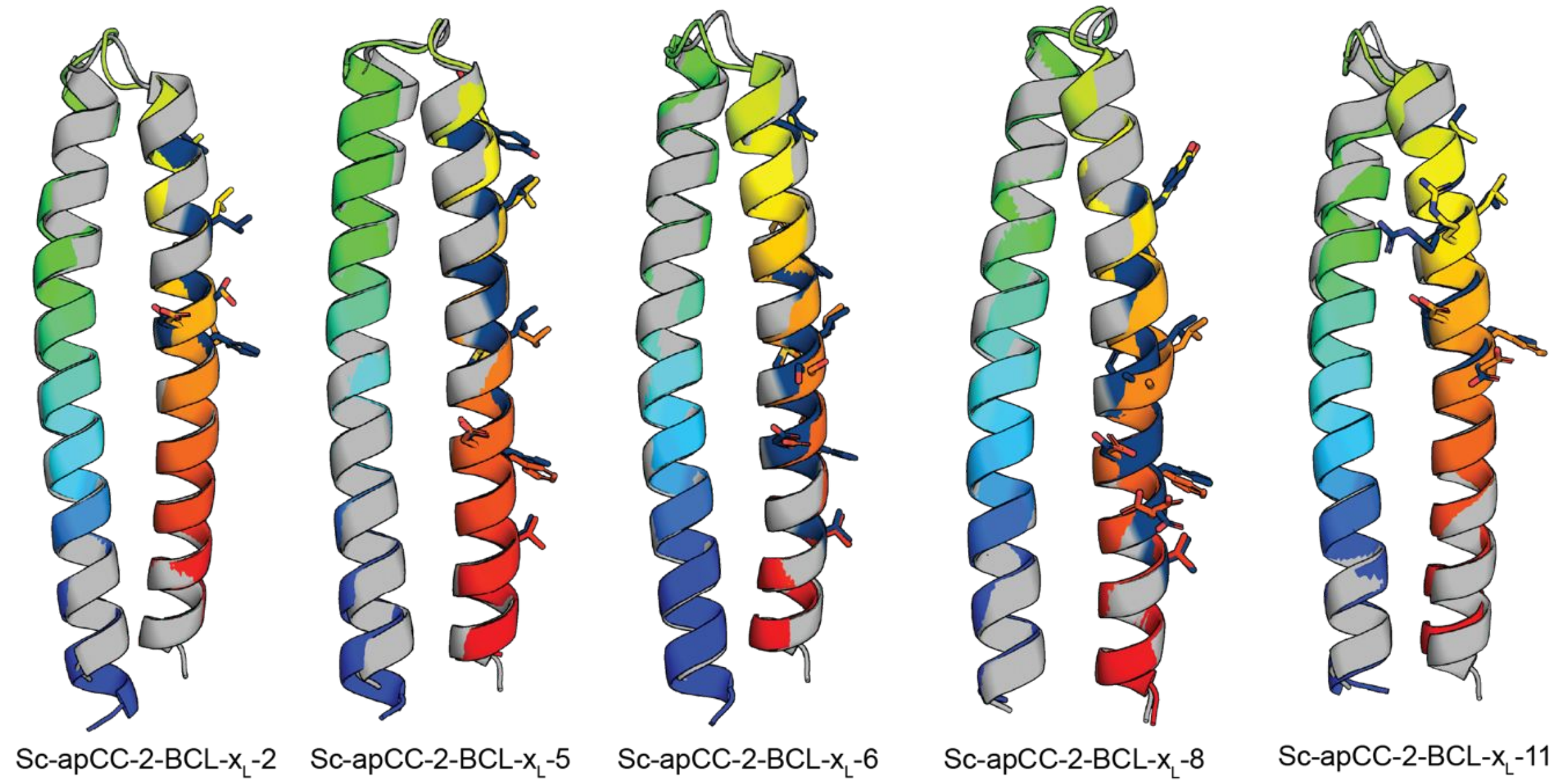

Supplementary Figure 5. Crystal structures of BCL-x_L designs. (PDB ids 9TLN, 9TLO, 9TLQ, 9TLR)

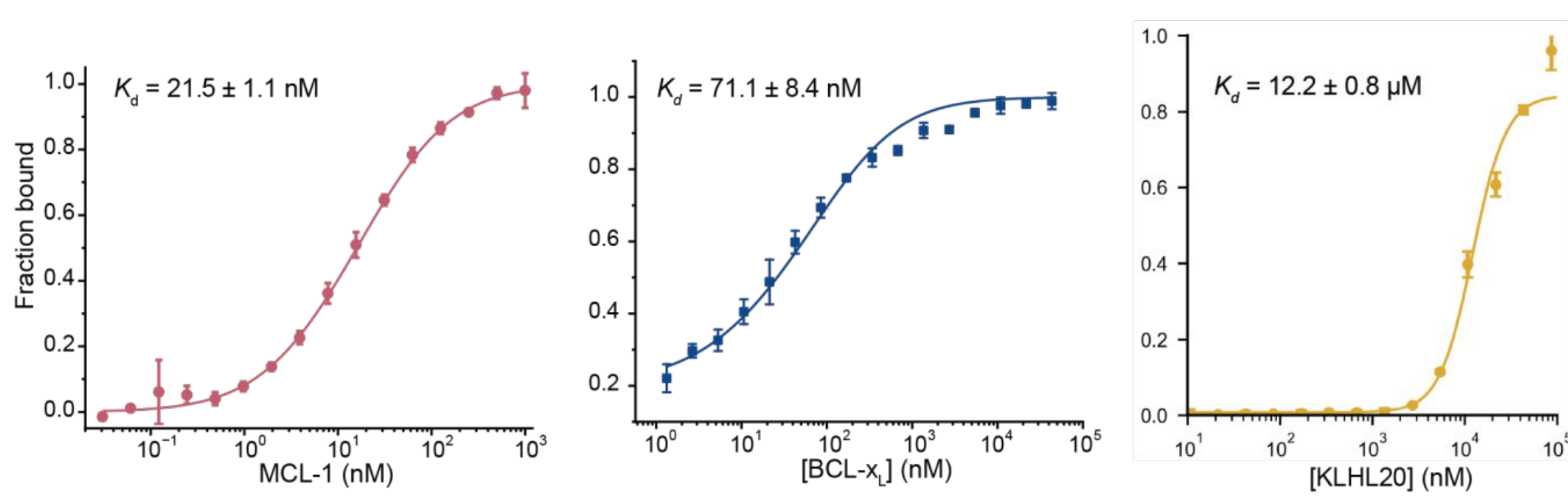

Supplementary Figure 6 Fluorescence anisotropy assay of direct titration of tracers. From left to right BID against MCL-1 (red), BID against BCL-x_L (dark blue) and DAPK1 against KLHL20 (yellow)

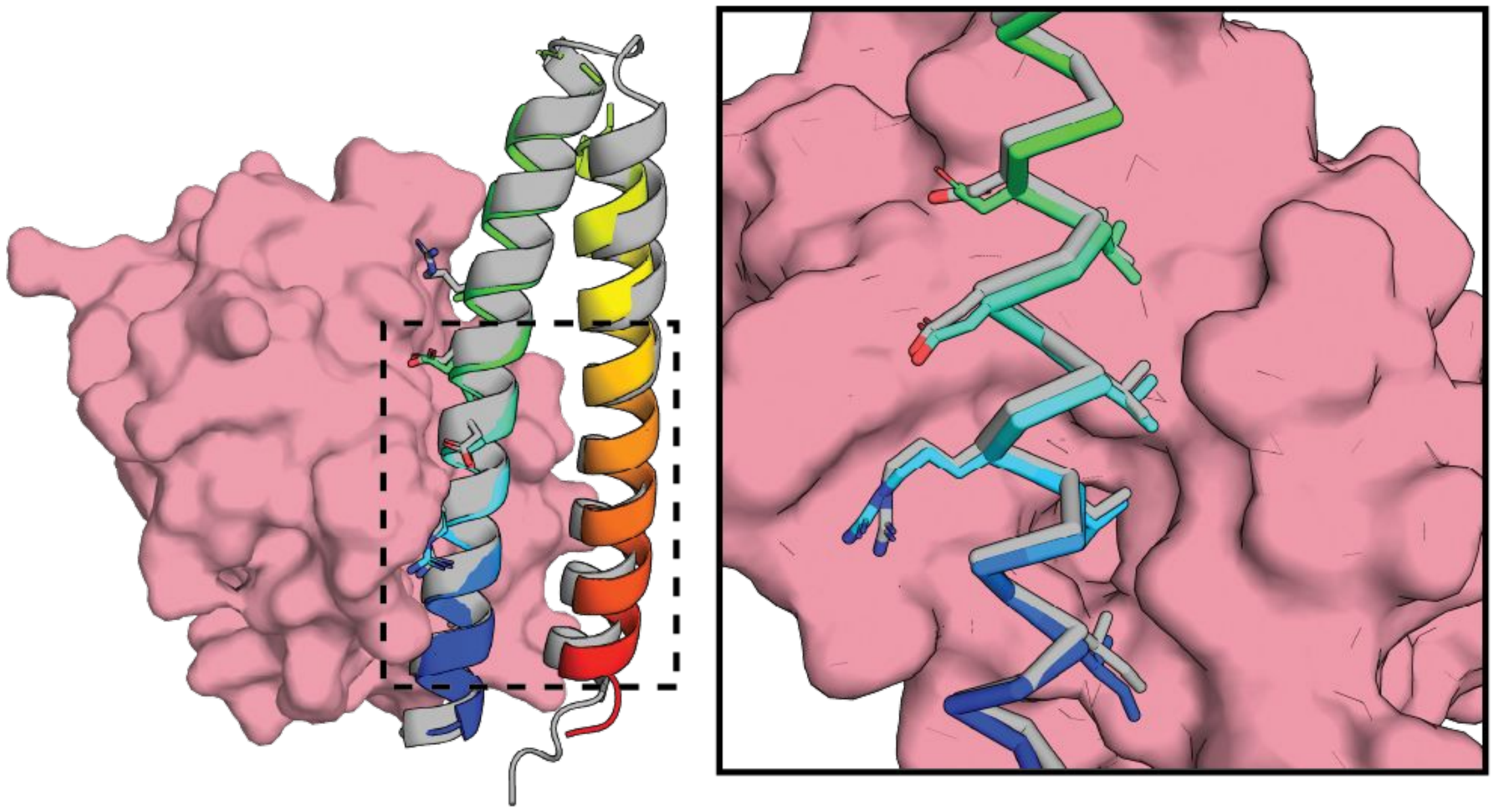

Supplementary Figure 7. Crystal structures of Sc-apCC-2-MCL-1-4 in complex with MCL-1. (PDB id 9TLW)

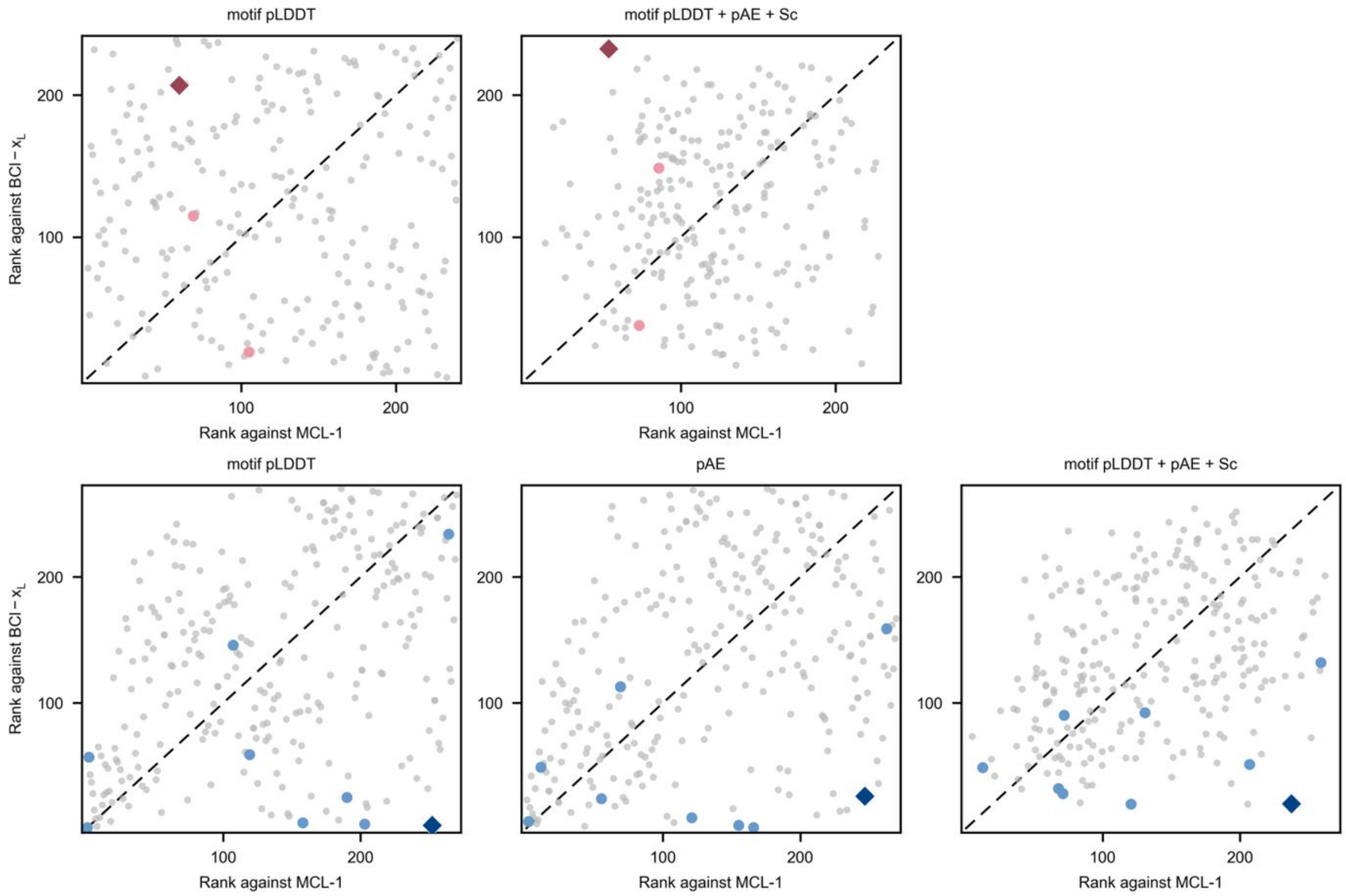

Supplementary Figure 8. Selectivity scatterplots for MCL-1 and BCL-x_L selective designs. Each grey dot represents a single computational designed sequence. Its y-axis position is determined by its rank in a selected metric against BCL-x_L, and its x-axis position by the rank against MCL-1. The selected metric is shown as the title. The colored dots represent sequences selected in the first design round. The diamond represents the sequence selected based on this metric.

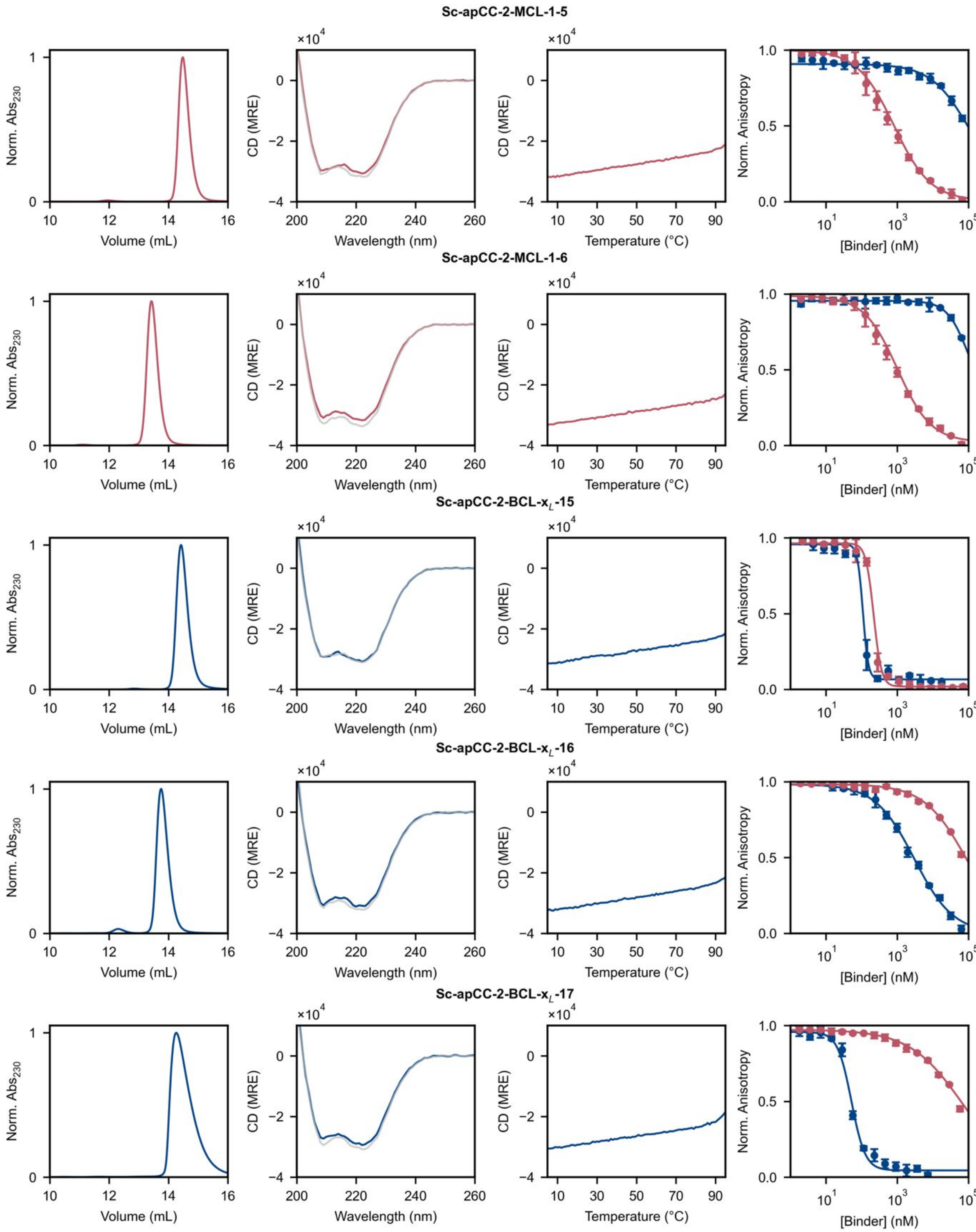
Sc-apCC-2-MCL-1-5
Sc-apCC-2-MCL-1-6
Sc-apCC-2-BCL-x$_L$-15
Sc-apCC-2-BCL-x$_L$-16
Sc-apCC-2-BCL-x$_L$-17
Norm. Abs$_{230}$
Volume (mL)
CD (MRE)
×10^4
Wavelength (nm)
Temperature (°C)
Norm. Anisotropy
[Binder] (nM)

Supplementary Figure 9. Biophysics and fluorescent anisotropy data of designs selected for specificity. From left to right analytical size exclusion chromatography Superdex 75 increase, signal is shown as normalized UV absorbance at 230 nm; protein concentration 100 µM in 20 mM Tris-HCl, 150 mM NaCl, pH 7.5. CD spectrum; CD thermal melt; protein concentration 20 µM in 20 mM Tris-HCl, 150 mM NaCl, pH 7.5 FA assay against BCL-x_L (dark blue) and MCL-1 (red) in 20 mM Tris-HCl, 150 mM NaCl, pH 7.5.

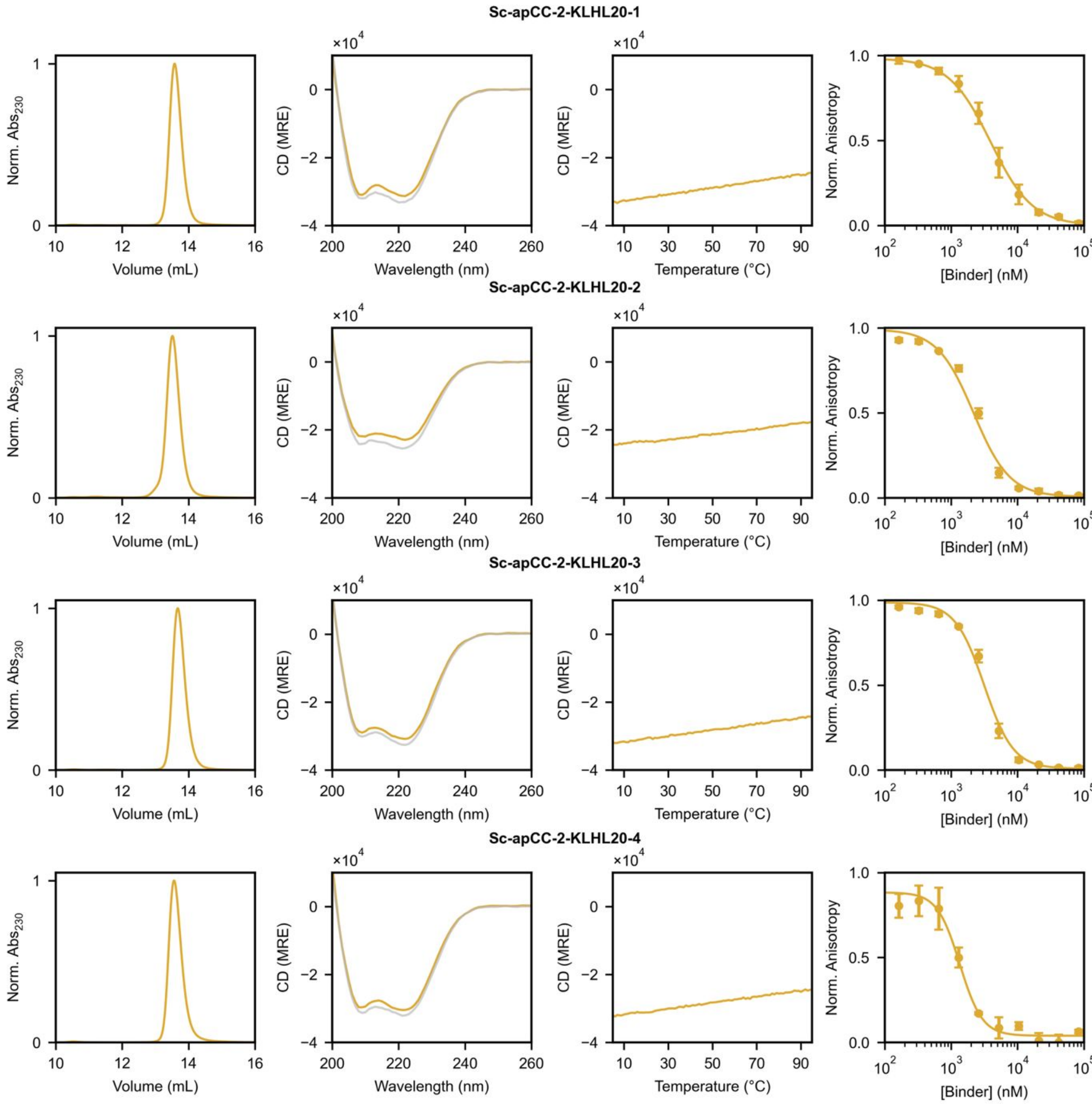

Supplementary Figure 10. Biophysics and fluorescent anisotropy data of designs against KLHL20. From left to right analytical size exclusion chromatography Superdex 75 increase, signal is shown as normalized UV absorbance at 230 nm; protein concentration 100 µM in 20 mM Tris-HCl, 150 mM NaCl, pH 7.5. CD spectrum; CD thermal melt; protein concentration 20 µM in 20 mM Tris-HCl, 150 mM NaCl, pH 7.5. FA assay against KLHL20 in 20 mM Tris-HCl, 150 mM NaCl, pH 7.5

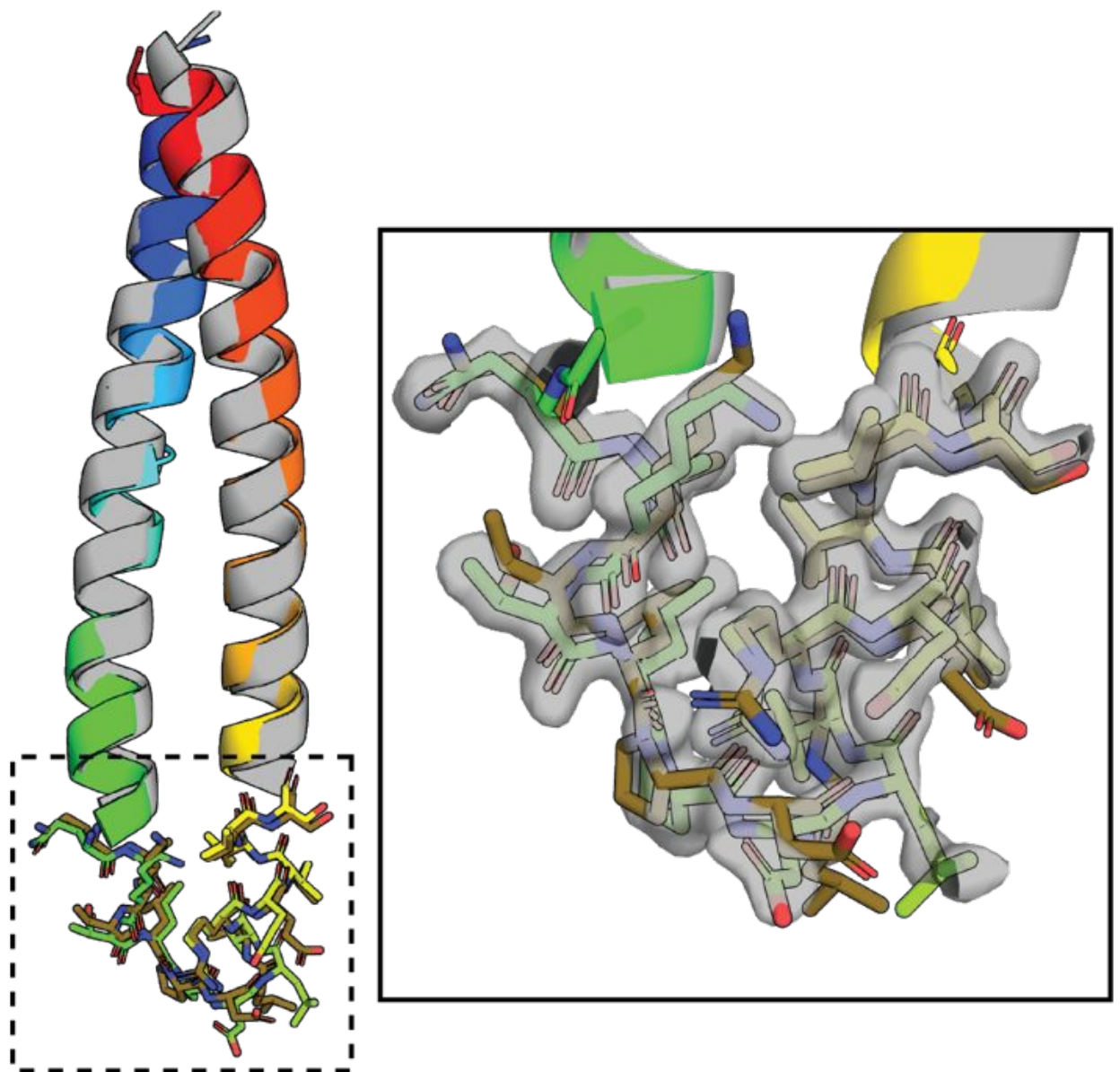

Supplementary Figure 11. Crystal structure of Sc-apCC-2-KLHL20-4. Zoom in shows electron density around the designed loop at sigma level 1 (PDB id 9TLT).

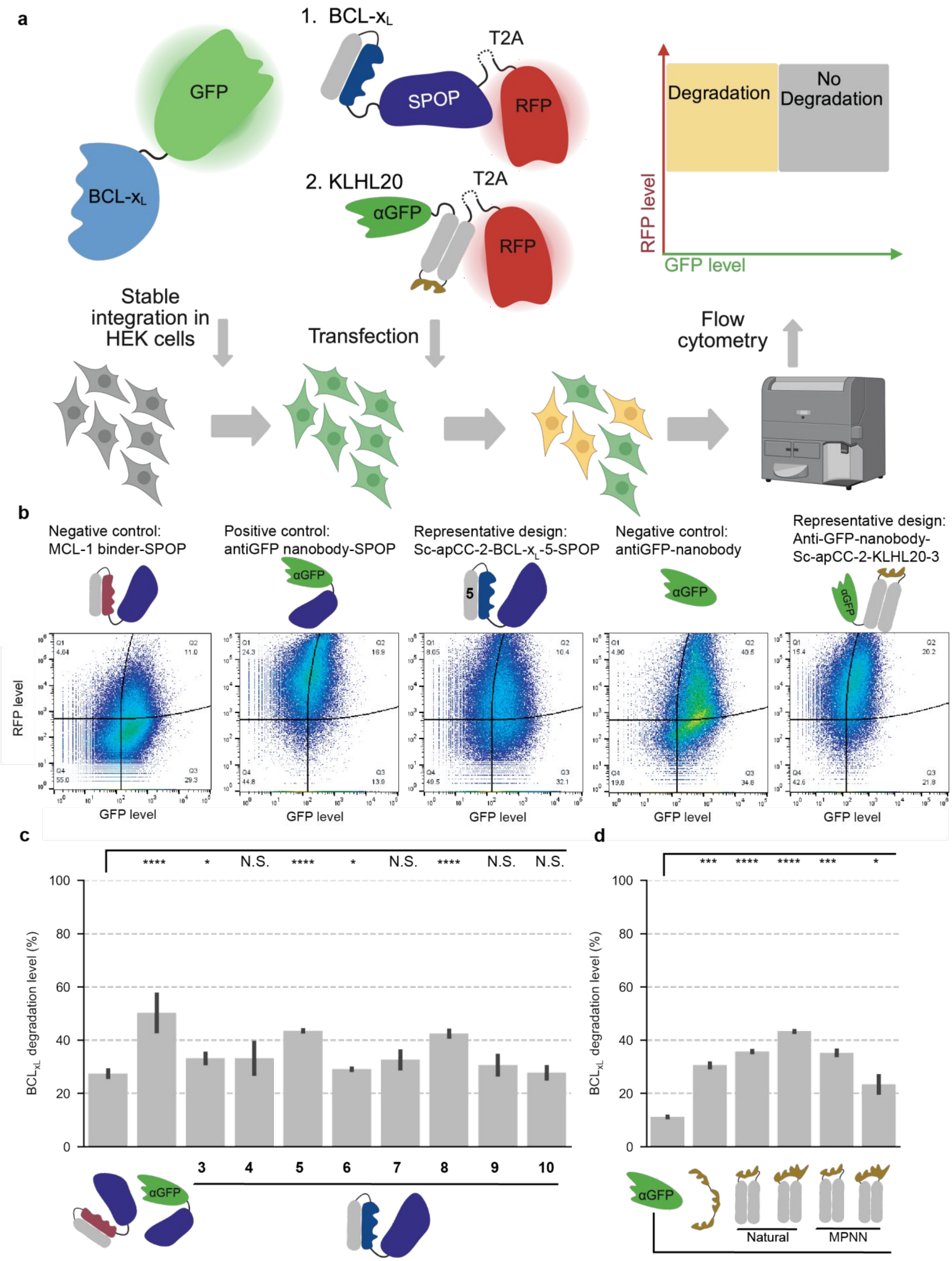
a
1. BCL-xL
T2A
SPOP
RFP
GFP
BCL-xL
2. KLHL20
T2A
αGFP
RFP
Degradation
No Degradation
RFP level
GFP level
Stable integration in HEK cells
Transfection
Flow cytometry
b
Negative control: MCL-1 binder-SPOP
Positive control: antiGFP nanobody-SPOP
Representative design: Sc-apCC-2-BCL-xL-5-SPOP
Negative control: antiGFP-nanobody
Representative design: Anti-GFP-nanobody-Sc-apCC-2-KLHL20-3
RFP level
GFP level
c
**** * N.S. **** * N.S. **** N.S. N.S.
BCLxL degradation level (%)
3 4 5 6 7 8 9 10
d
*** **** **** *** *
BCLxL degradation level (%)
αGFP
Natural
MPNN

Supplementary Figure 12. Degradation assay of BCL-x$_L$-GFP with BCL-x$_L$ binders and KLHL20 binders. a, Scheme of the GFP based assay, HEK293 cells were engineered to express BCL-x$_L$-GFP fusion proteins under an inducible promotor. Afterwards the cells were transfected either with fusion proteins consisting of the E3 ligase SPOP and a BCL-x$_L$ binder design with an RFP present after a self-cleaving T2A sequence, or fusions of an anti-GFP nanobody and a KLHL20 binding design with the same RFP sequence. These cells are subsequently measured in a flow cytometer. An increase in RFP signals expression of the degrader while decrease of GFP corresponds to success. **b,** 2D dot plots of representative measurements. From left to right negative control (Sc-apCC-2-MCL-1-1-SPOP), positive control (αGFP-SPOP), Sc-apCC-2-BCL-x$_L$-5-SPOP, negative control KLHL20 experiment (αGFP), αGFP-Sc-apCC-2-KLHL20-3. **c,** Histograms of BCL-x$_L$ designs, y-axis shows the level of BCL-x$_L$ calculated as 100 x Q2/(Q1 +Q2). Significance against the negative control was calculated by a two-sided Welch T-test. **d,** Histograms KLHL20 designs, y-axis shows the same calculated BCL-x$_L$ level. Significance levels were calculated with a two-sided Welch T-test against the negative control. Significance levels are represented as N.S.: $P > 0.05$, ∗: $P \leq 0.05$, ∗∗: $P \leq 0.01$, ∗∗∗: $P \leq 0.001$, ∗∗∗∗: $P \leq 0.0001$. The list of all used constructs is shown in table 8. Parts of this figure were made with BioRender.

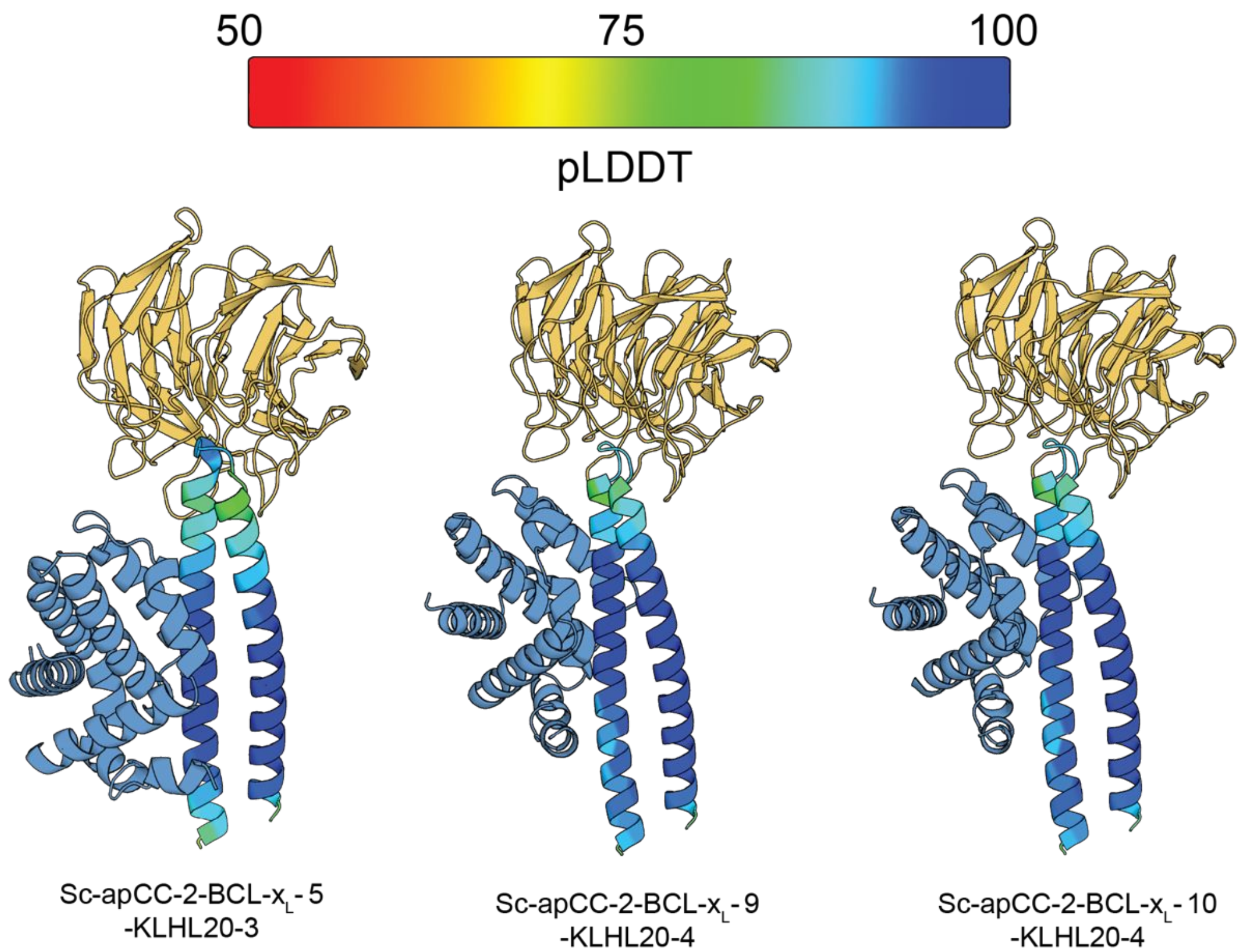

Supplementary Figure 13. Predicted ternary complexes of bifunctional binders. AlphaFold2 models of the ternary complexes between BCL-x$_L$ (blue), KLHL20 (yellow) and the bifunctional binders used. The binders are colored according to AlphaFold2 pLDDT scores following the colorbar shown on top.

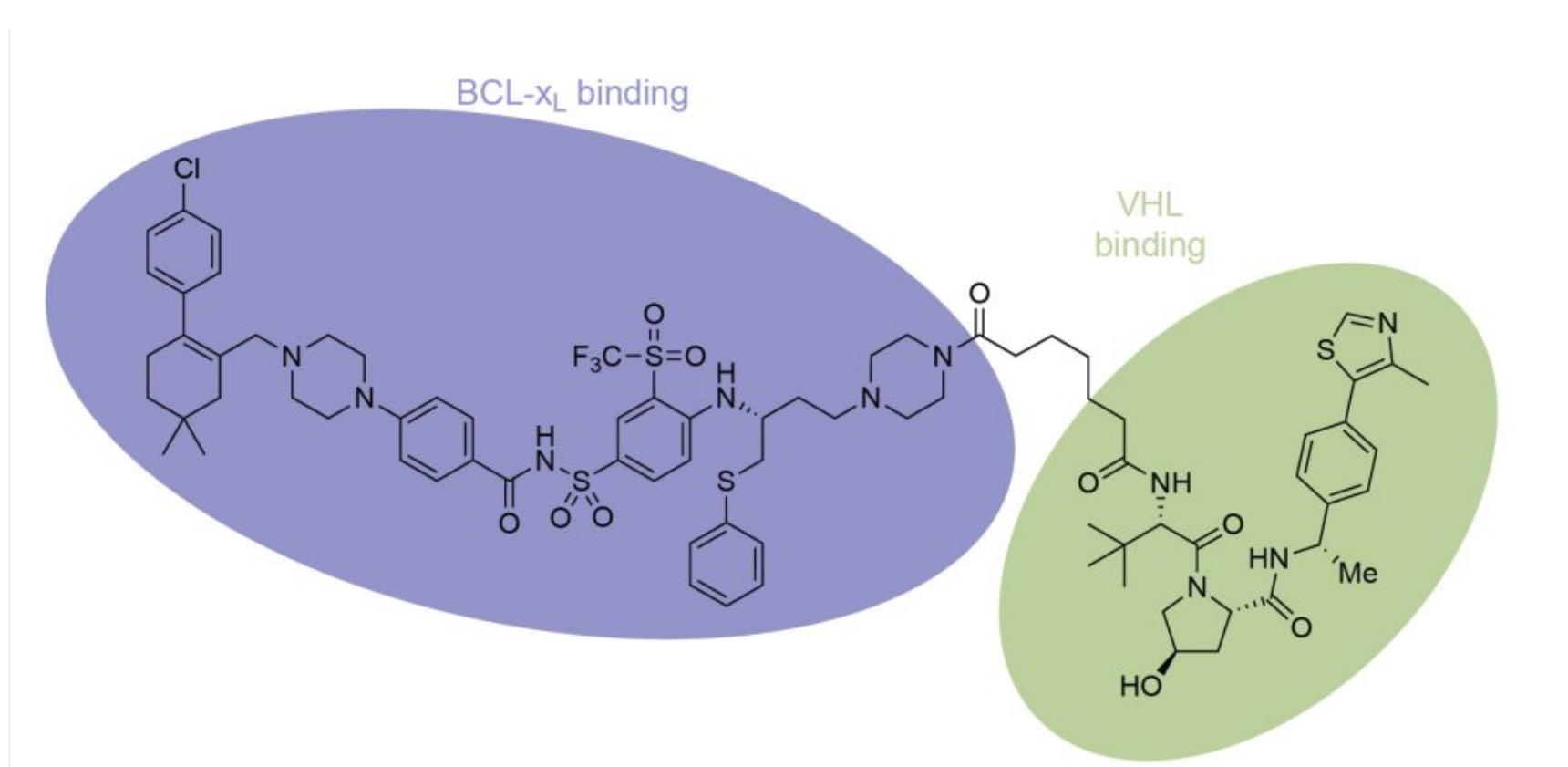

Supplementary Figure 14. Chemical structure of DT2216

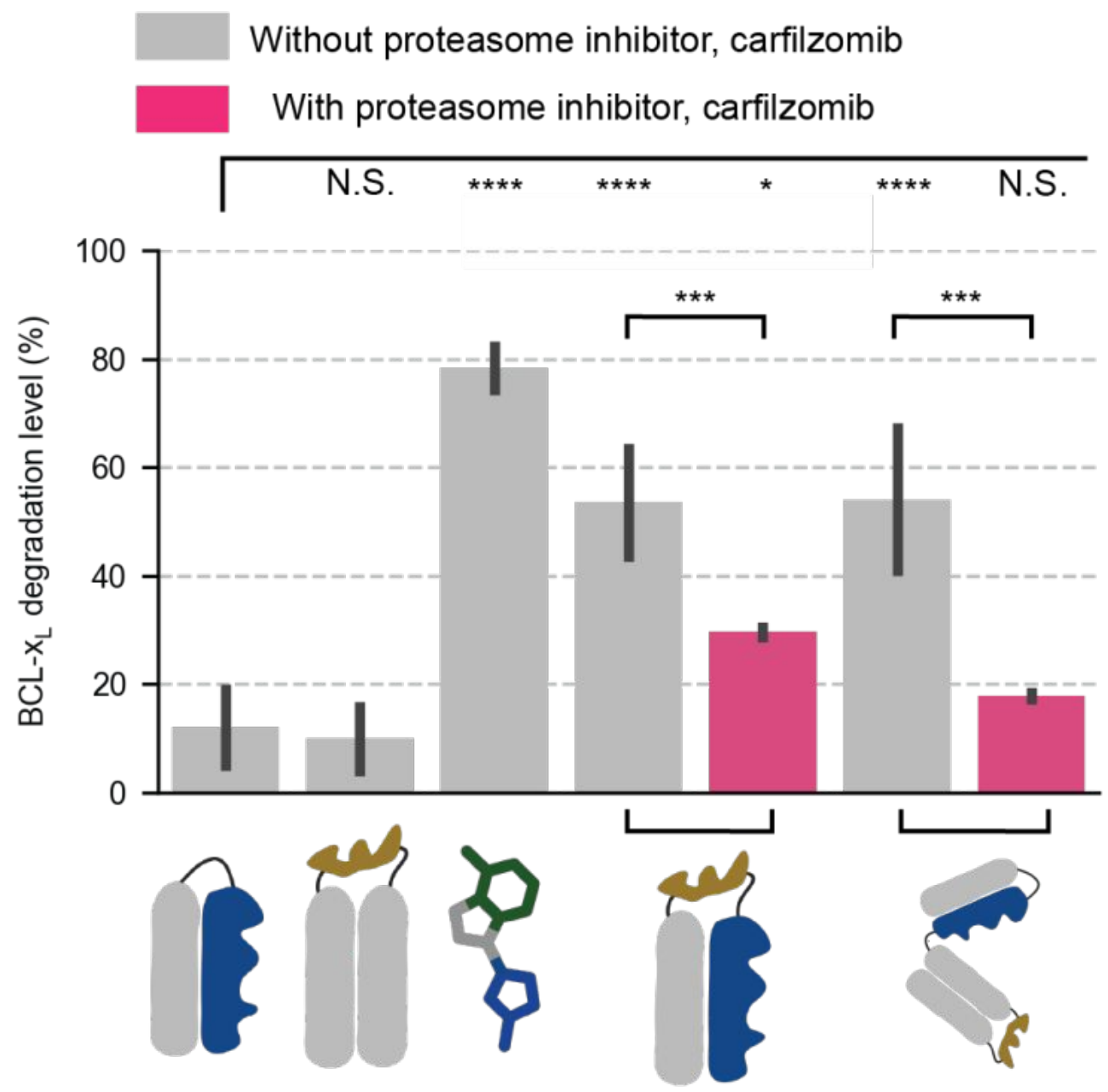

Supplementary Figure 15. Endogenous degradation with carfilzomib as proteasome inhibitor. Histograms showing BCL-x$_L$ degradation in the A549 cell line. From left to right: the monofunctional controls, Sc-apCC-2-BCL-x$_L$-5 (12%) , Sc-apCC-2-KLHL20-3 (10%), DT2216 as positive control (78%), bifunctional hairpin Sc-apCC-2-BCL-x$_L$-5-KLHL20-3 (54%) and fusion construct of both monofunctional hairpins Sc-apCC-2-BCL-x$_L$-5_sc-apCC-2-KLHL20-3 (54%). The pink bars correspond to the tests with addition of the proteasome inhibitor, resulting in 30% degradation level for Sc-apCC-2-BCL-x$_L$-5-KLHL20-3 and 18% degradation level for Sc-apCC-2-BCL-x$_L$-5_sc-apCC-2-KLHL20-3. Significance levels calculated with a Welch t-test against the monofunctional BCL-x$_L$ binder shown on top. The significance level between the samples with and without the inhibitor was also calculated with a Welch T-test. Significance levels are

represented as N.S.: P > 0.05, ∗: P ≤ 0.05, ∗∗: P ≤ 0.01, ∗∗∗: P ≤ 0.001, ∗∗∗∗: P ≤ 0.0001. Parts of this figure were made with BioRender.

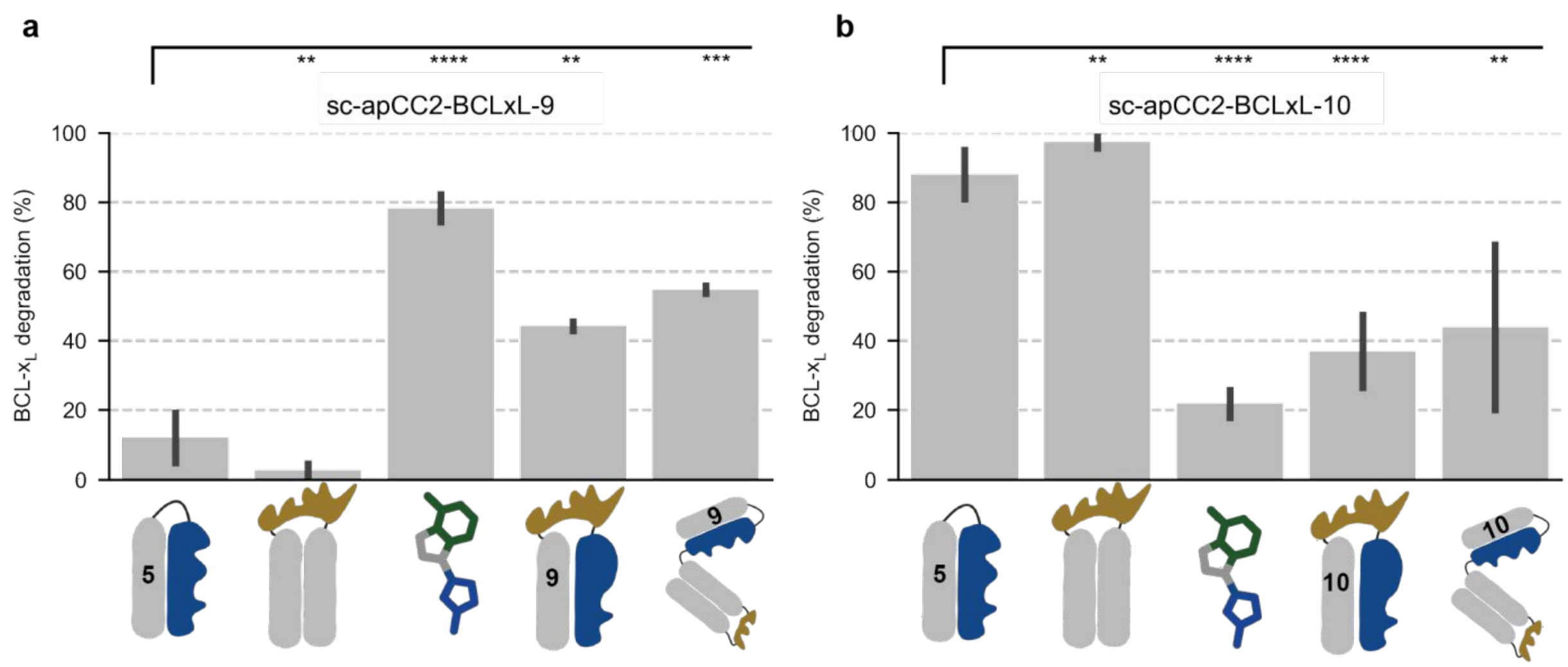

Supplementary Figure 16. Additional endogenous degradation constructs. Histograms showing BCL-x_L degradation in the A549 cell line. **a,** constructs based on Sc-apCC-2-BCL-x_L-9 and Sc-apCC-2-KLHL20-3. Average degradation level values from left to right: 12%, 3%, 78%, 44%, 55%. **b,** constructs based on Sc-apCC-2-BCL-x_L-10 and Sc-apCC-2-KLHL20-4. Average degradation level values from left to right: 12%, 3%, 78%, 63%, 56%. Significance levels are represented as N.S.: P > 0.05, ∗: P ≤ 0.05, ∗∗: P ≤ 0.01, ∗∗∗: P ≤ 0.001, ∗∗∗∗: P ≤ 0.0001. Parts of this figure were made with BioRender.

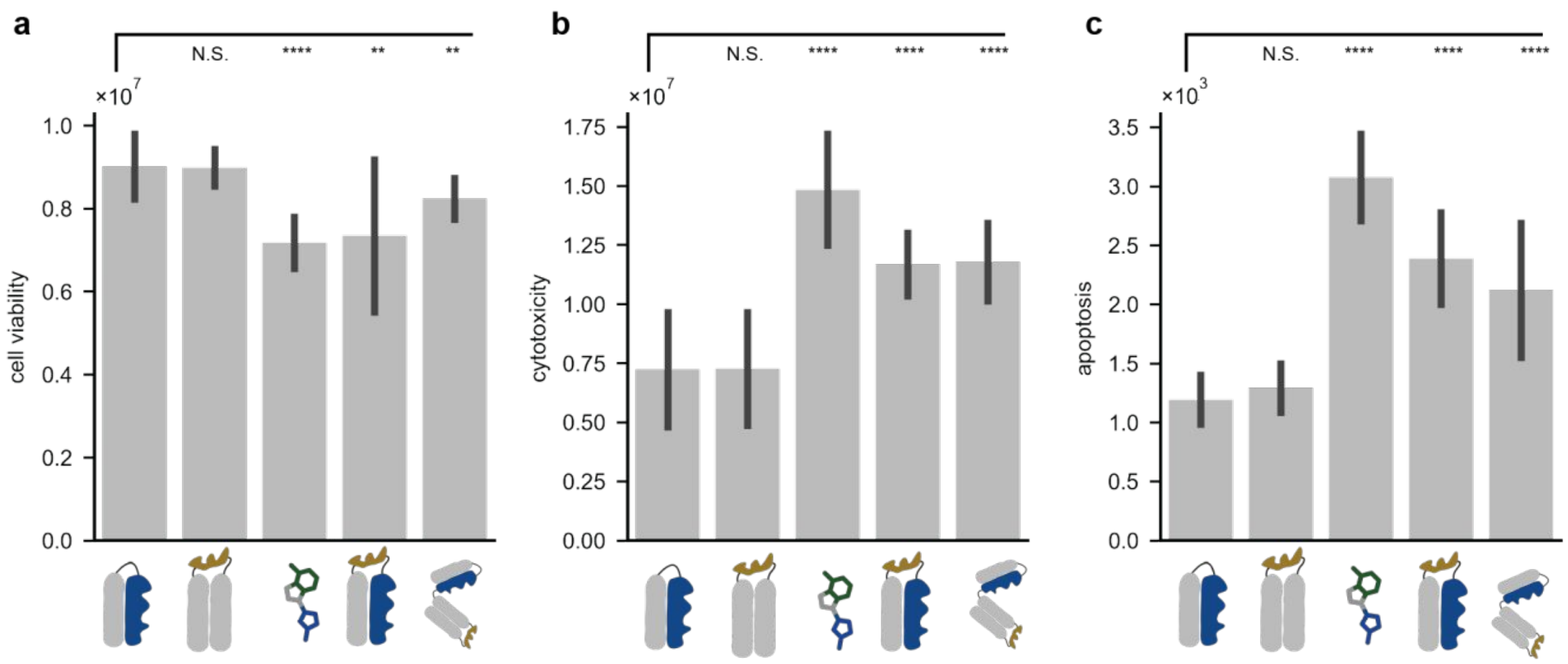

Supplementary Figure 17. ApoTox-Glo™ Triplex Assay with Sc-apCC-2-BCL-x$_L$-5-KLHL20-3 constructs. a, cell viability **b,** Cytotoxicity **c,** apoptosis. Significance levels are represented as N.S.: P > 0.05, ∗: P ≤ 0.05, ∗∗: P ≤ 0.01, ∗∗∗: P ≤ 0.001, ∗∗∗∗: P ≤ 0.0001. Parts of this figure were made with BioRender.

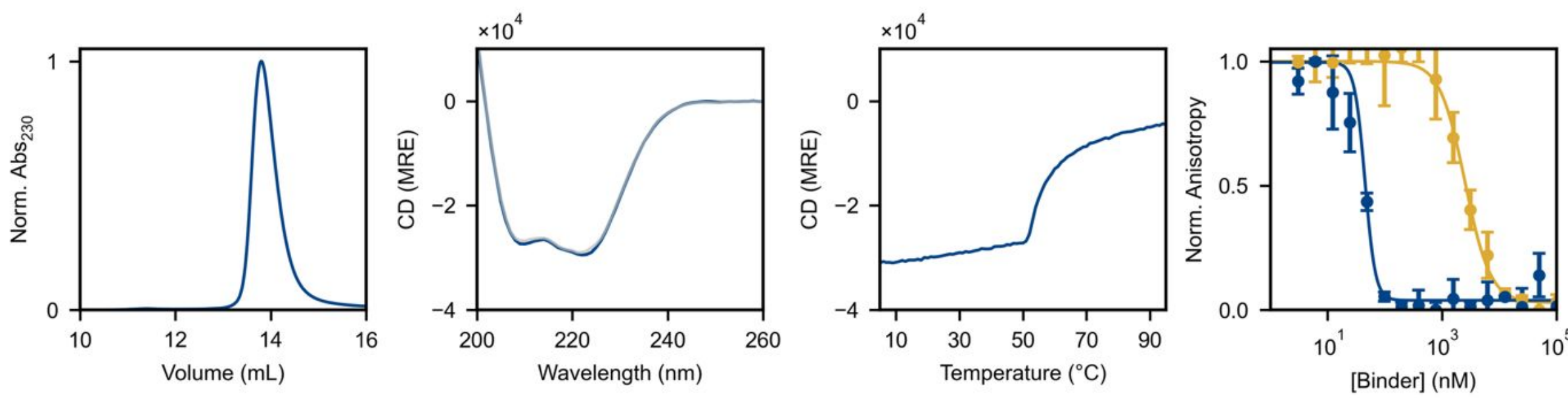

Supplementary Figure 18. Biophysics and fluorescent anisotropy data of bifunctional Sc-apCC-2-BCL-x$_L$-5-KLHL20-3. From left to right analytical size exclusion chromatography Superdex 75 increase, signal is shown as normalized UV absorbance at 230 nm; protein concentration 100 µM in 20 mM Tris-HCl, 150 mM NaCl, pH 7.5. CD spectrum; CD thermal melt; protein concentration

20 μM in 20 mM Tris-HCl, 150 mM NaCl, pH 7.5. FA assay against BCL-x_L (dark blue) and KLHL20 (yellow) in 20 mM Tris-HCl, 150 mM NaCl, pH 7.5.

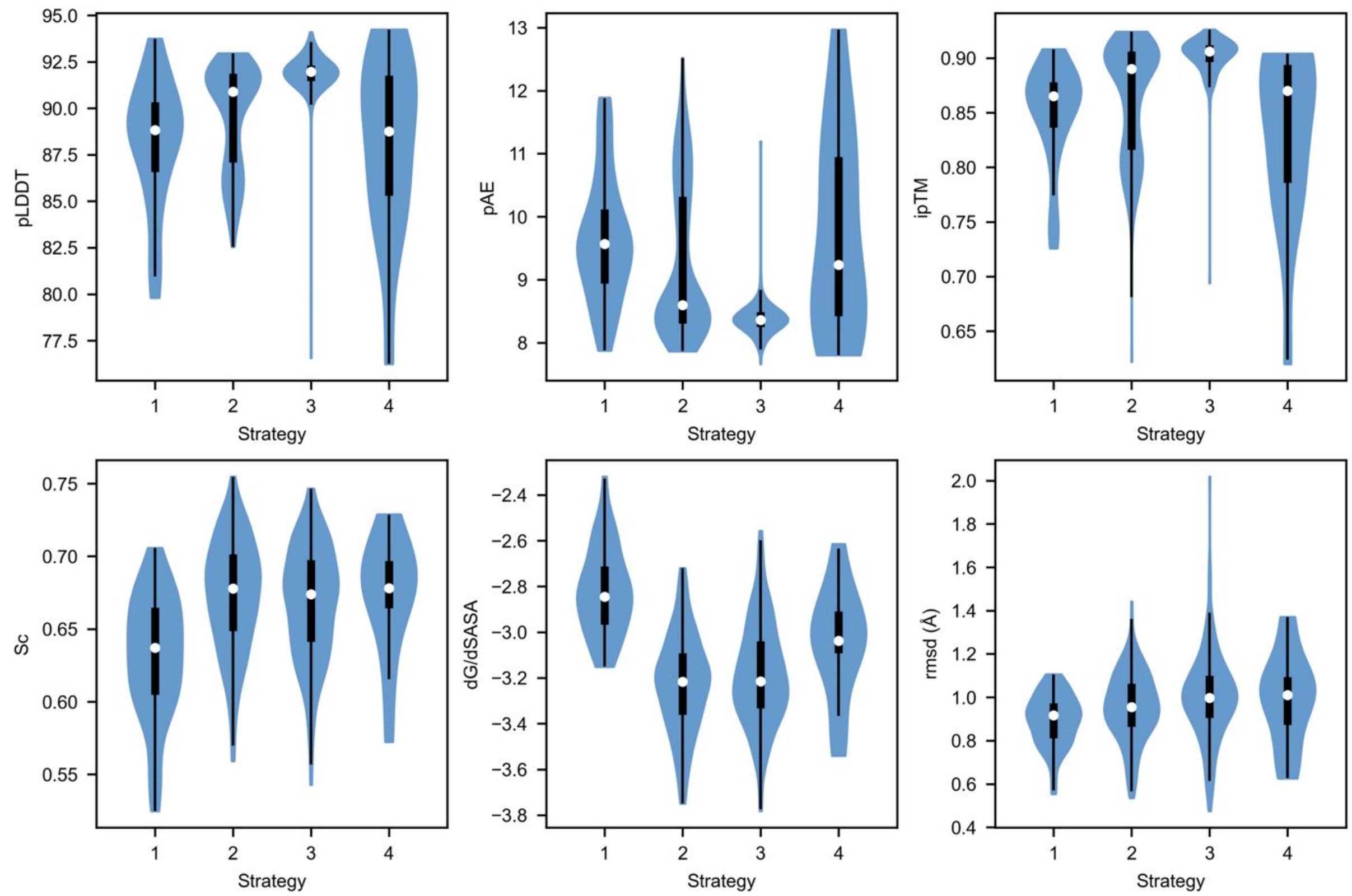

Supplementary Figure 19. Violin plots of *in silico* designed sequences against BCL-x_L following the different implementation strategy of ProteinMPNN Strategy 1 no-additional MPNN; strategy 2, additional MPNN within 6 Å of target; strategy 3, MPNN at all non-critical positions; strategy 4, MPNN at all non-critical positions and altering motif

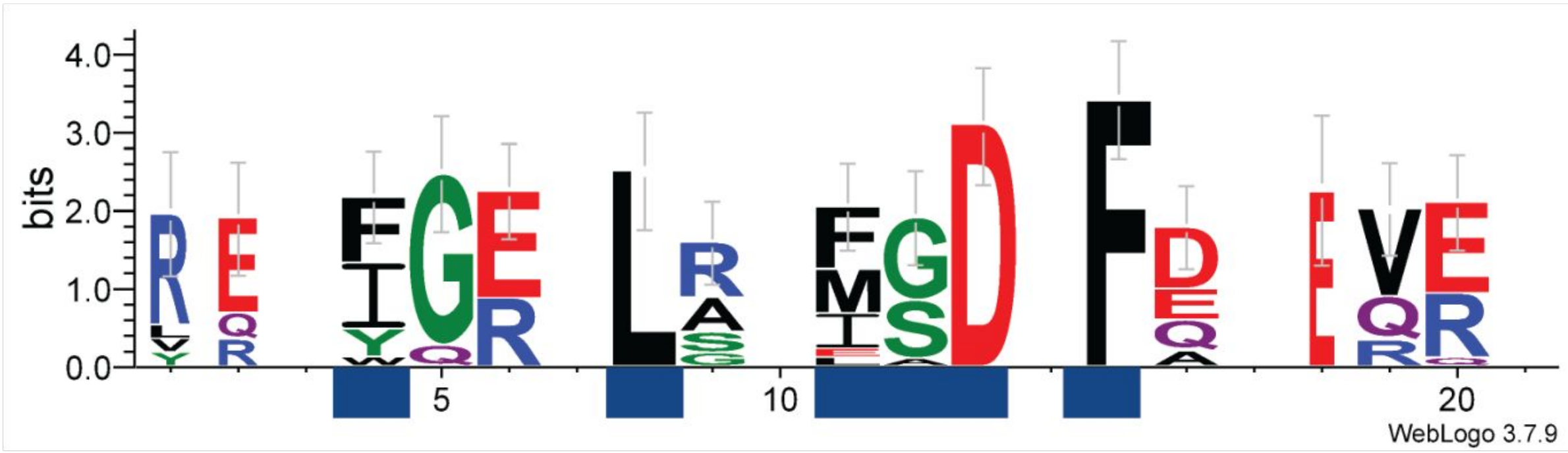

Supplementary Figure 20. Sequence logo of all experimentally tested designs against BCL-x$_L$. Blue boxes indicate motif positions. This weblogo was made with (https://weblogo.threeplusone.com)

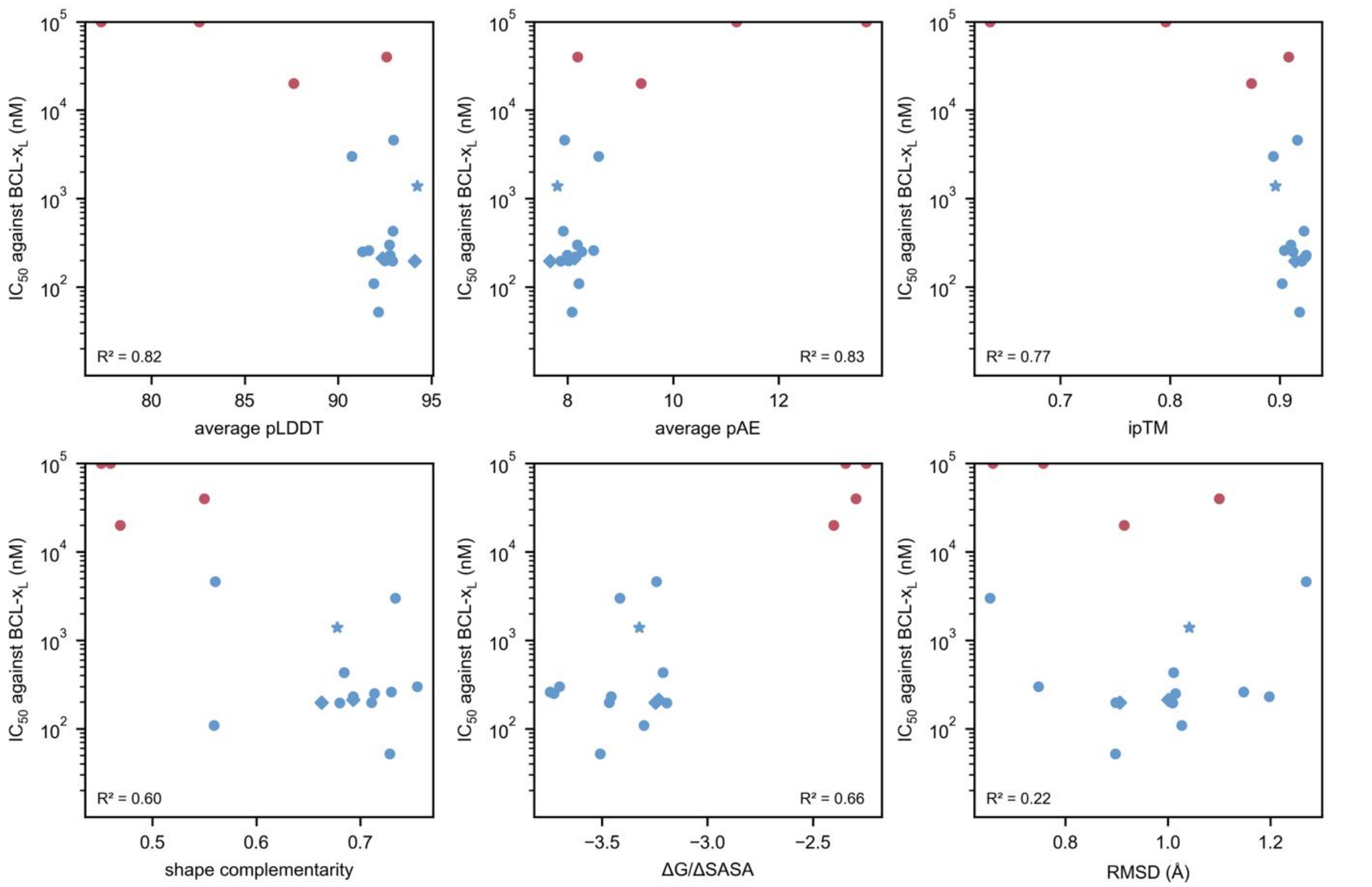

Supplementary Figure 21. Plots of measured IC_{50} values against BCL-x_L as a function of *in silico* metrics. IC_{50} values are plotted on a logarithmic scale. R^2 value of a linear correlation is shown at the bottom. Red dots correspond to sequences designed against MCL-1; Blue dots correspond to sequences designed by strategy 2; blue diamonds correspond to sequences designed by strategy 3; blue star corresponds to sequences designed by strategy 4.

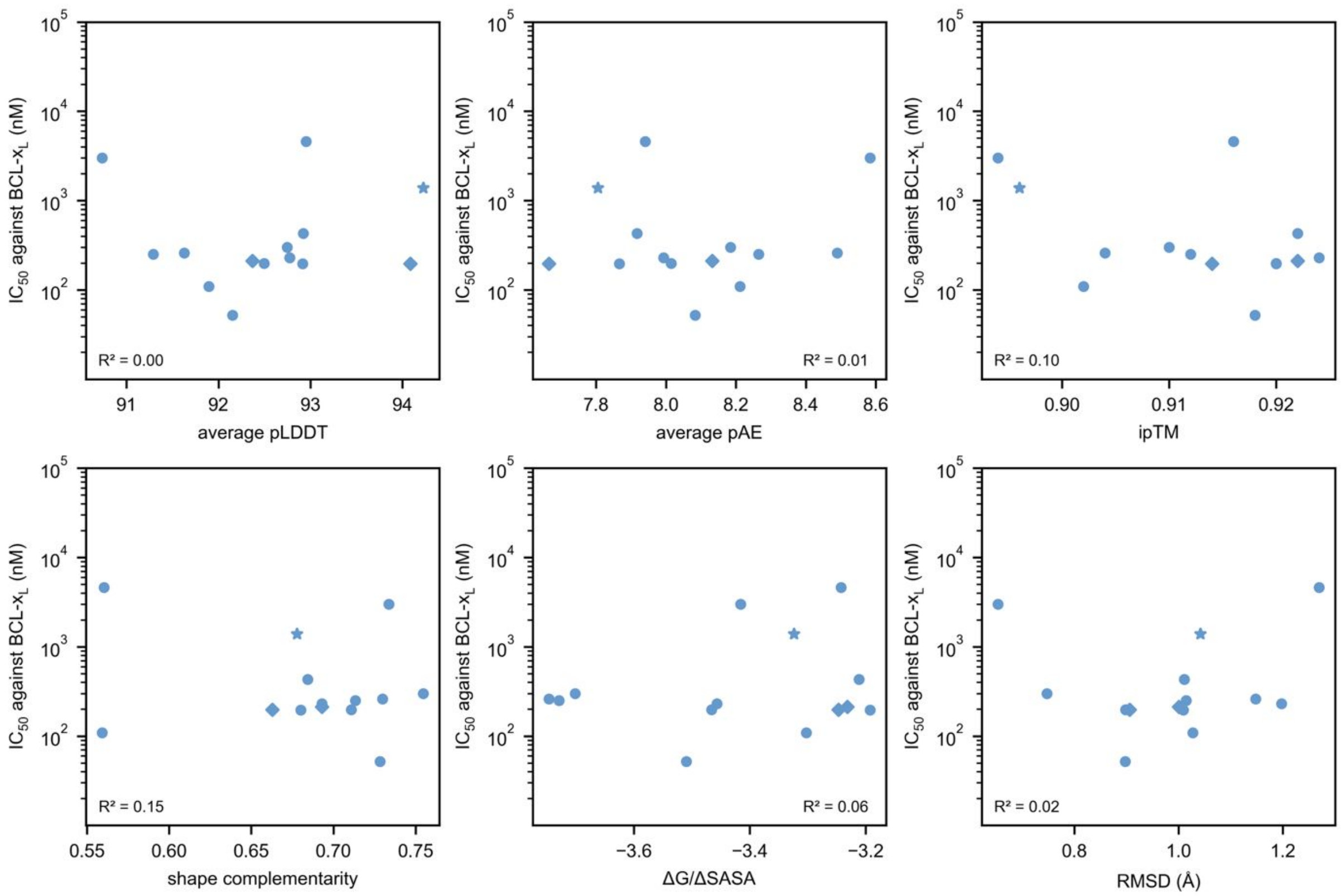

Supplementary Figure 22. Plots of measured IC_{50} values against BCL-x_L as a function of *in silico* metrics. Same as in Figure 19 but without including the MCL-1 designs. IC_{50} values are plotted on a logarithmic scale. R^2 value of a linear correlation is shown at the bottom. Blue dots correspond to sequences designed by strategy 2; blue diamonds correspond to sequences designed by strategy 3; blue star corresponds to sequences designed by strategy 4.

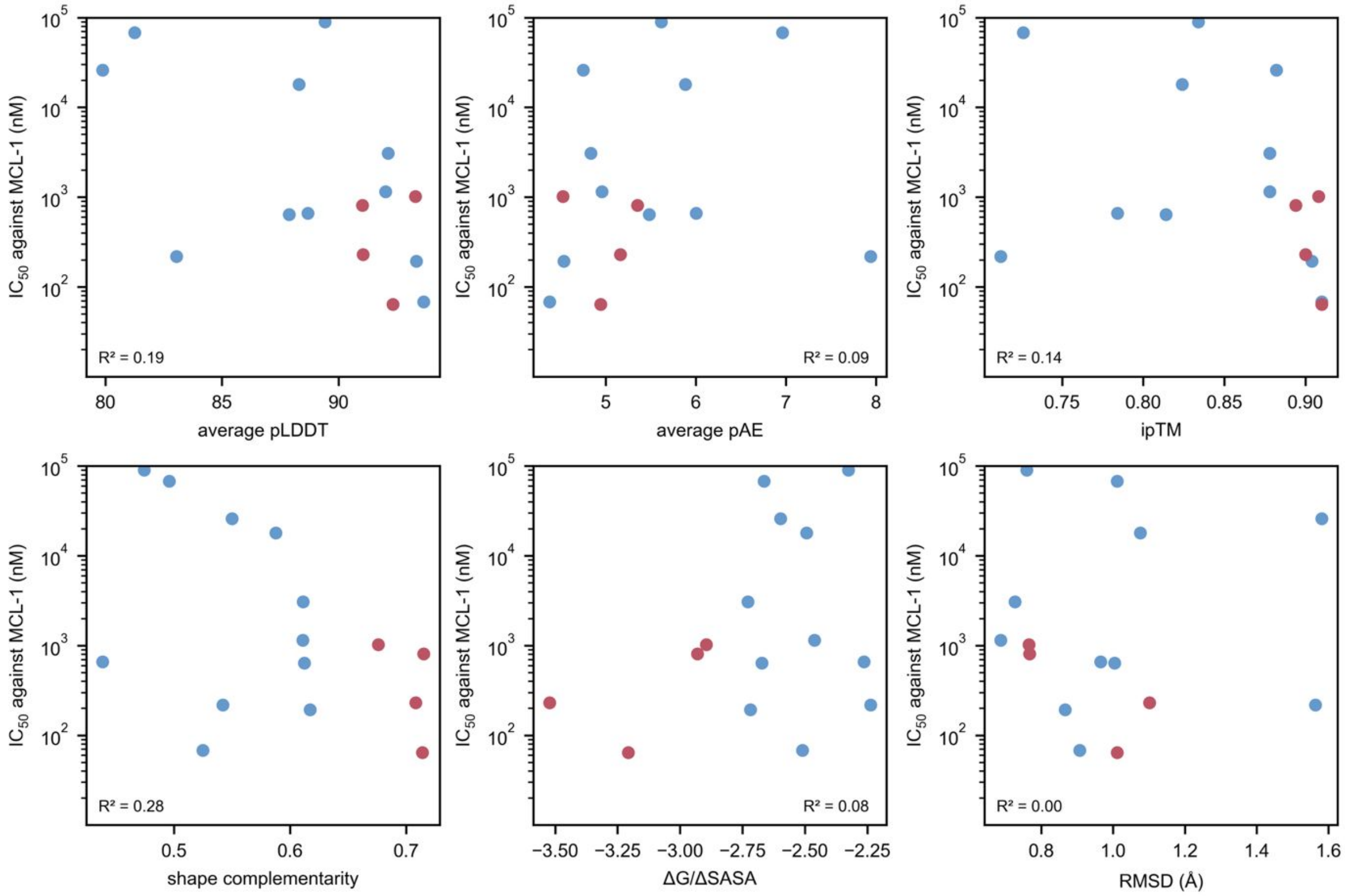

Supplementary Figure 23. Plots of measured IC_{50} values against MCL-1 as a function of *in silico* metrics. IC_{50} values are plotted on a logarithmic scale. R^2 value of a linear correlation is shown at the bottom. Red dots correspond to sequences designed against MCL-1; Blue dots correspond to sequences designed by strategy 2.

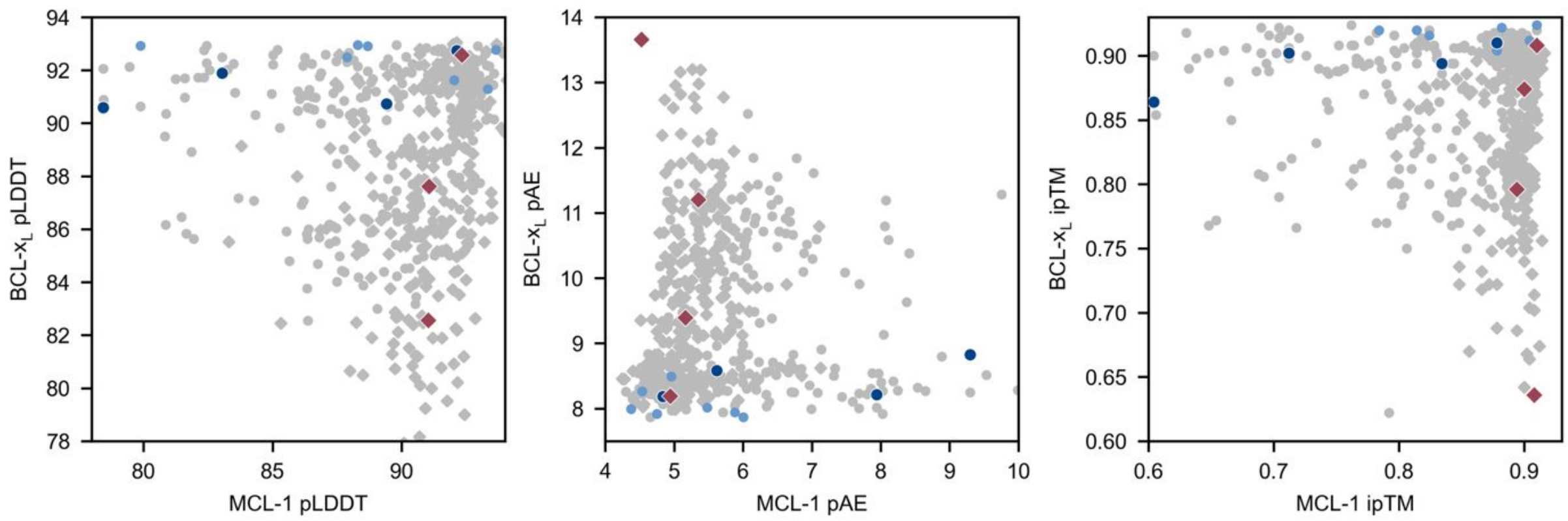

Supplementary Figure 24. Scatter plots of AlphaFold2 confidence metrics against MCL-1 and BCL-x$_L$. From left to right, pLDDT, pAE and ipTM. The sequences designed against BCL-x$_L$ are shown as grey dots (not tested), light blue dots (non-specific designs) and dark blue dots (specific designs IC$_{50}$ against MCL-1 < 10x IC$_{50}$ against BCL-x$_L$). The sequences designed against MCL-1 are shown as grey diamond (not tested), and red diamond (specific designs)

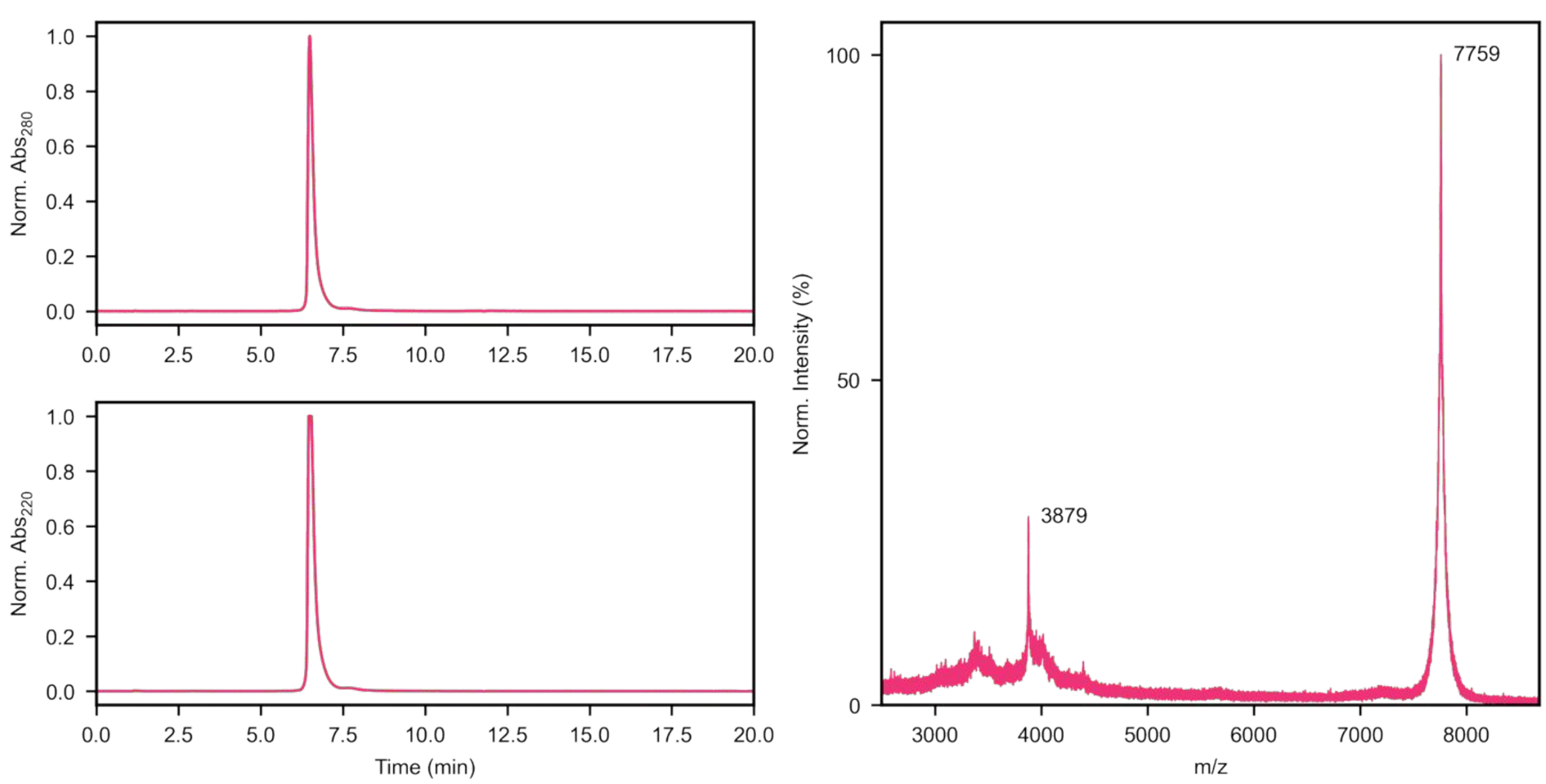

Supplementary Figure 25. Validation of Sc-apCC-2

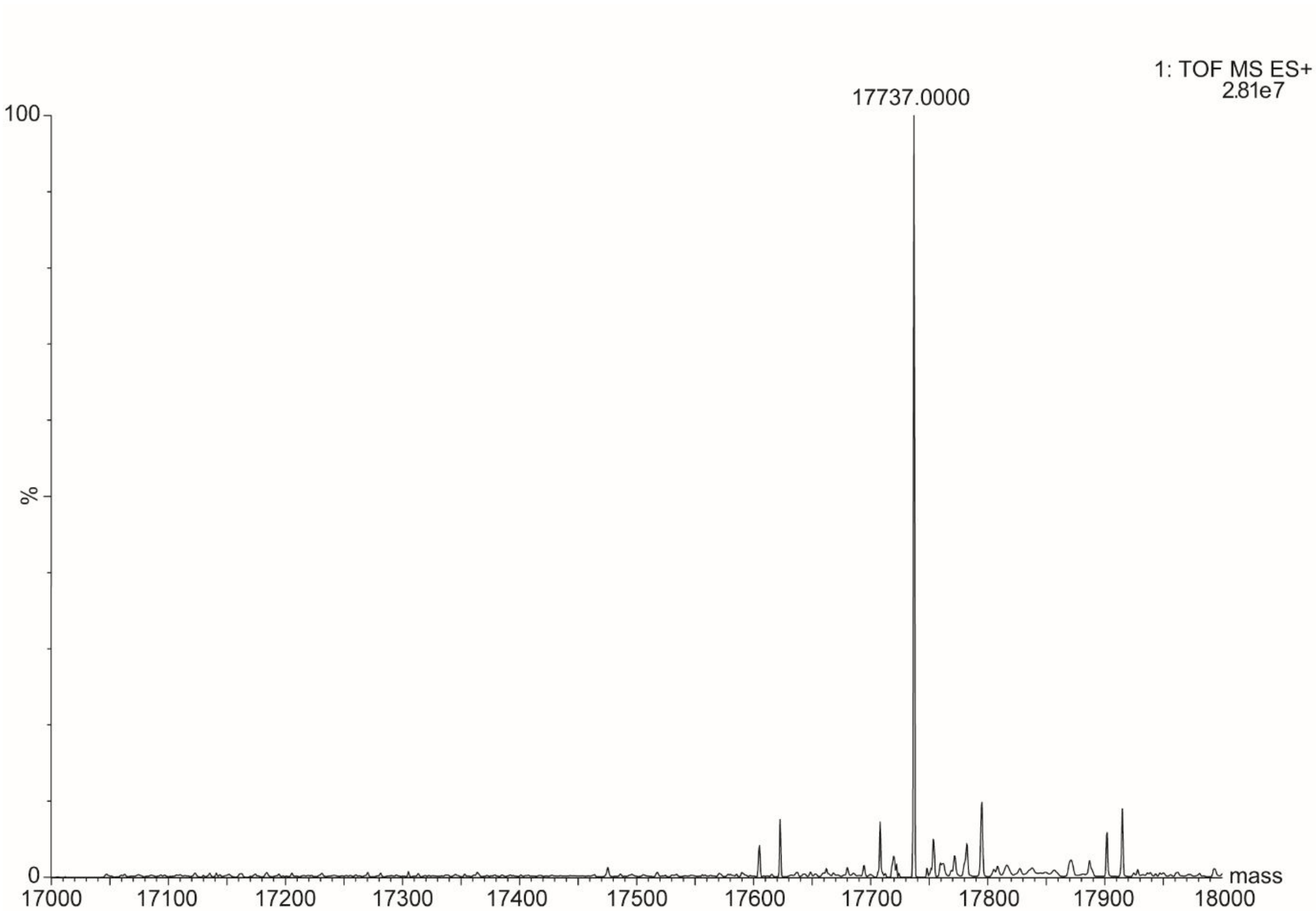

Supplementary Figure 26. Mass spectrometry of MCL-1. Predicted MW 17,737.2. Observed 17,737.0.

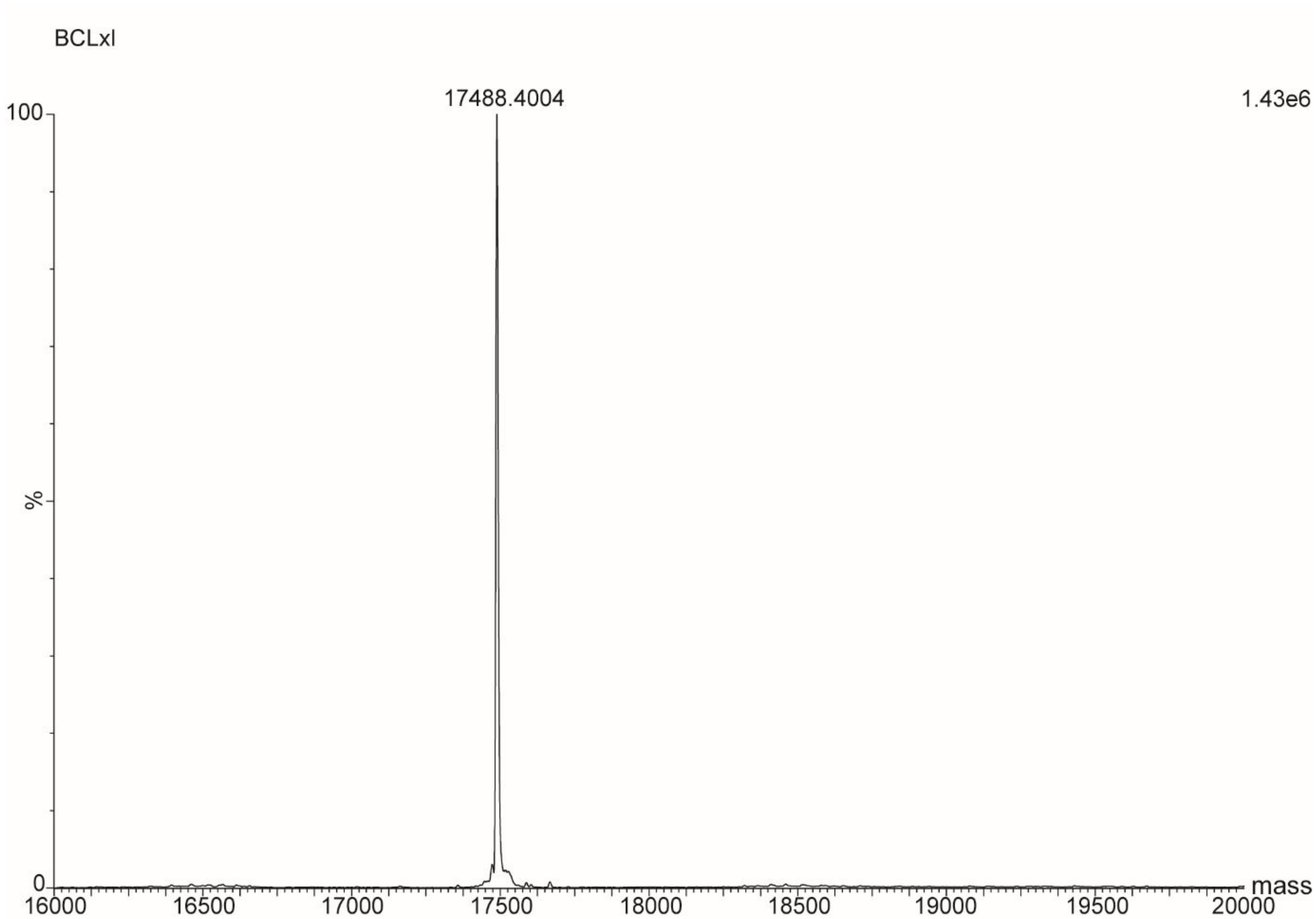

Supplementary Figure 27. Mass spectrometry result of BCL-x$_L$. Predicted 17,489.5. Observed 17488.4.

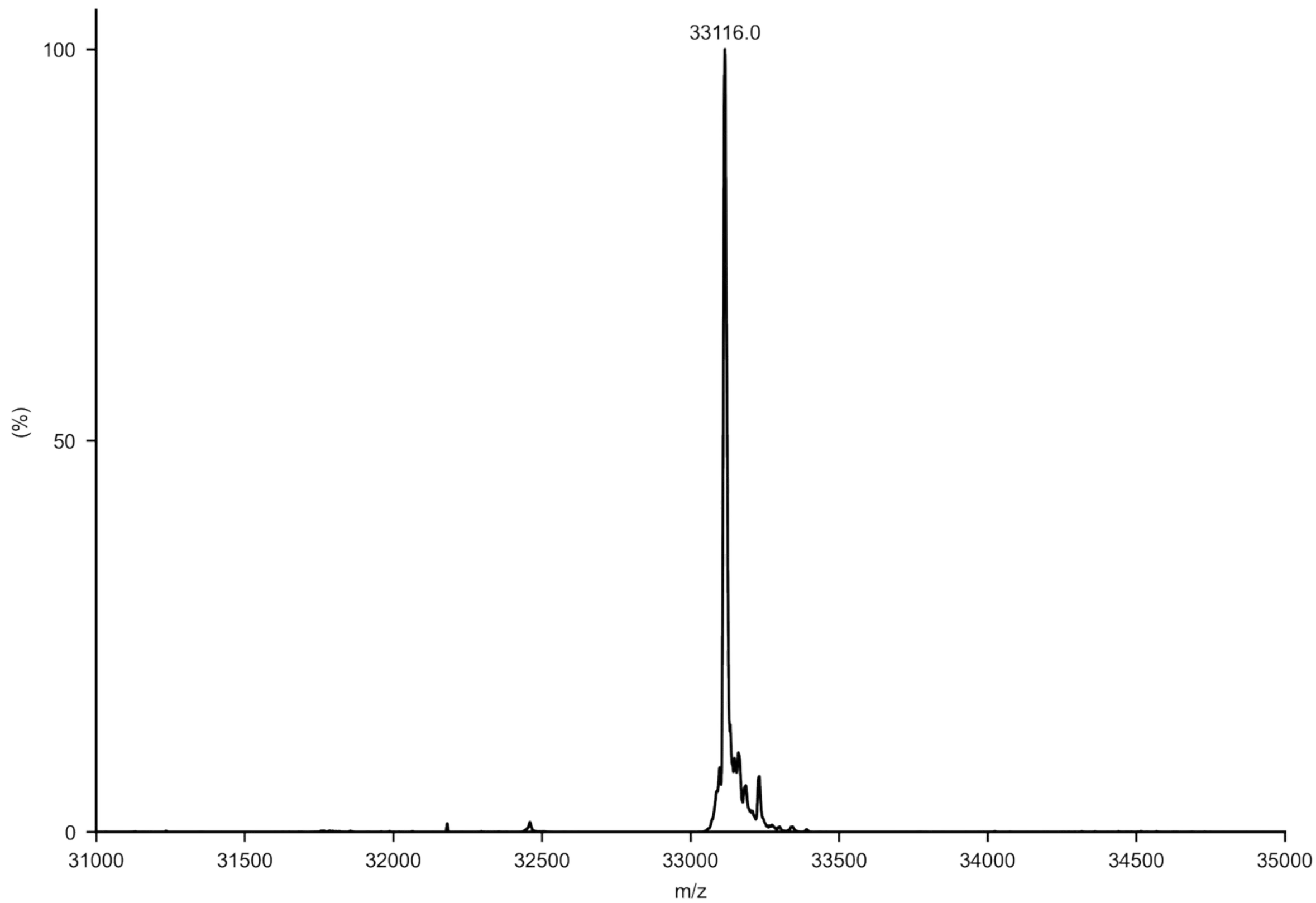

Supplementary Figure 28. Mass spectrometry result of KLHL20. Predicted 33117.0 Observed 33116.0

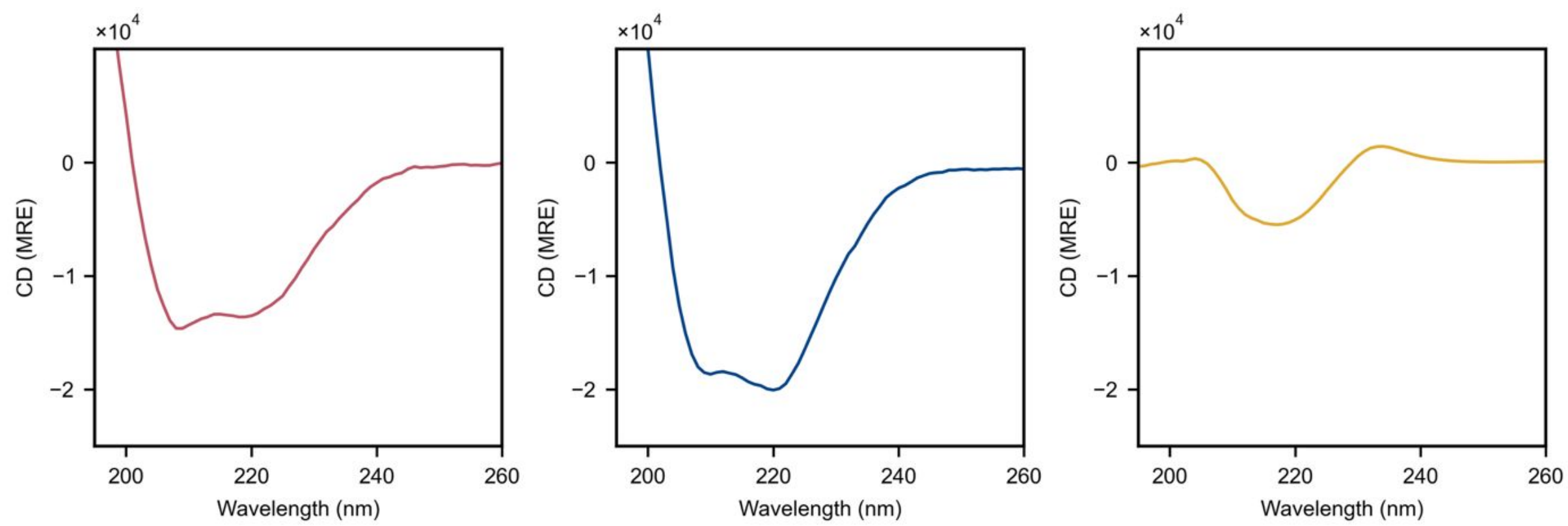

Supplementary Figure 29. CD spectrometry of binding targets. From left to right MCL-1 (red), BCL-x_L (blue) and KLHL20 (yellow)

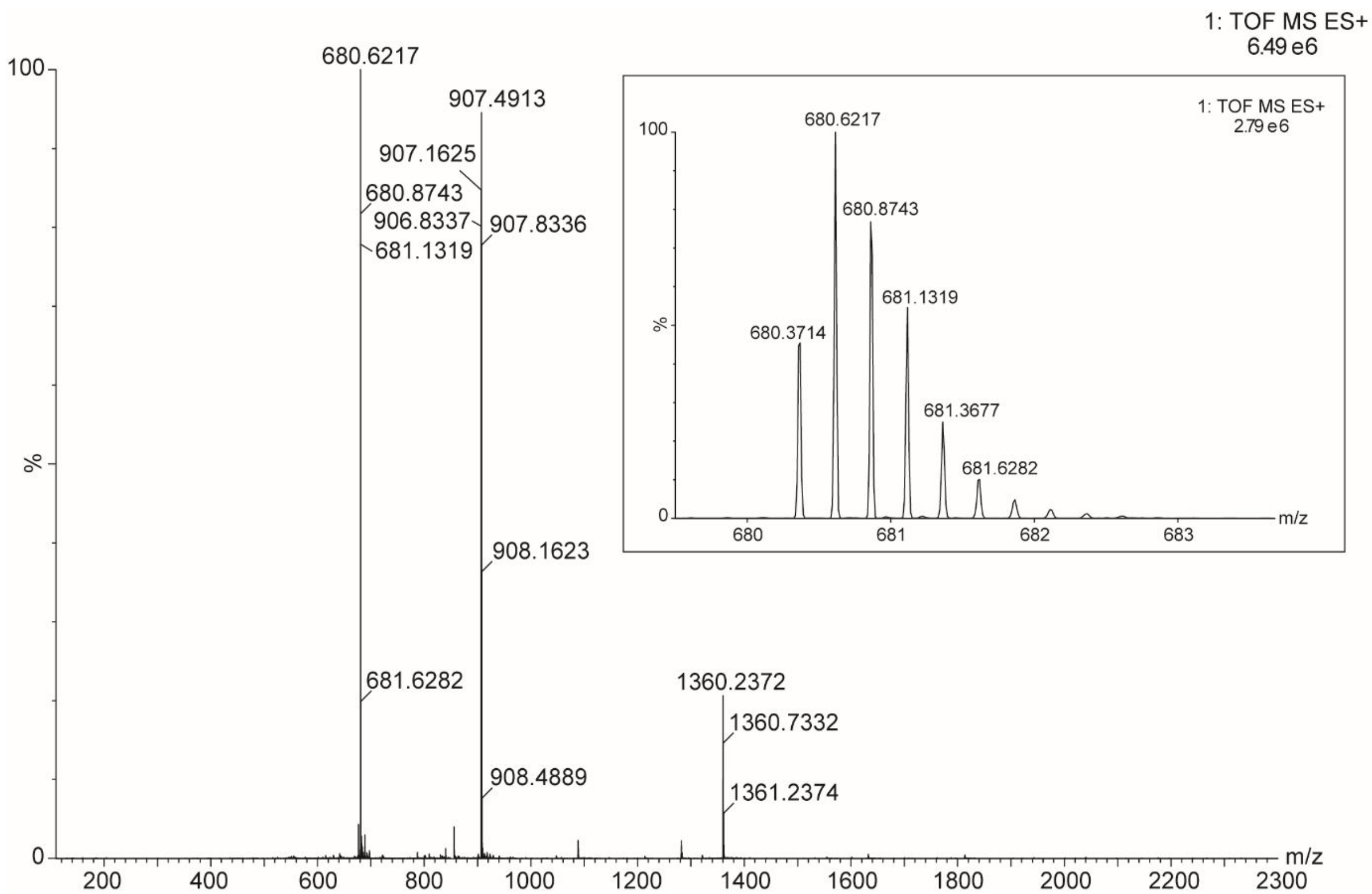

Supplementary Figure 30 Mass spectrometry result for BID. Predicted 2,717.441. Observed 2,717.446.

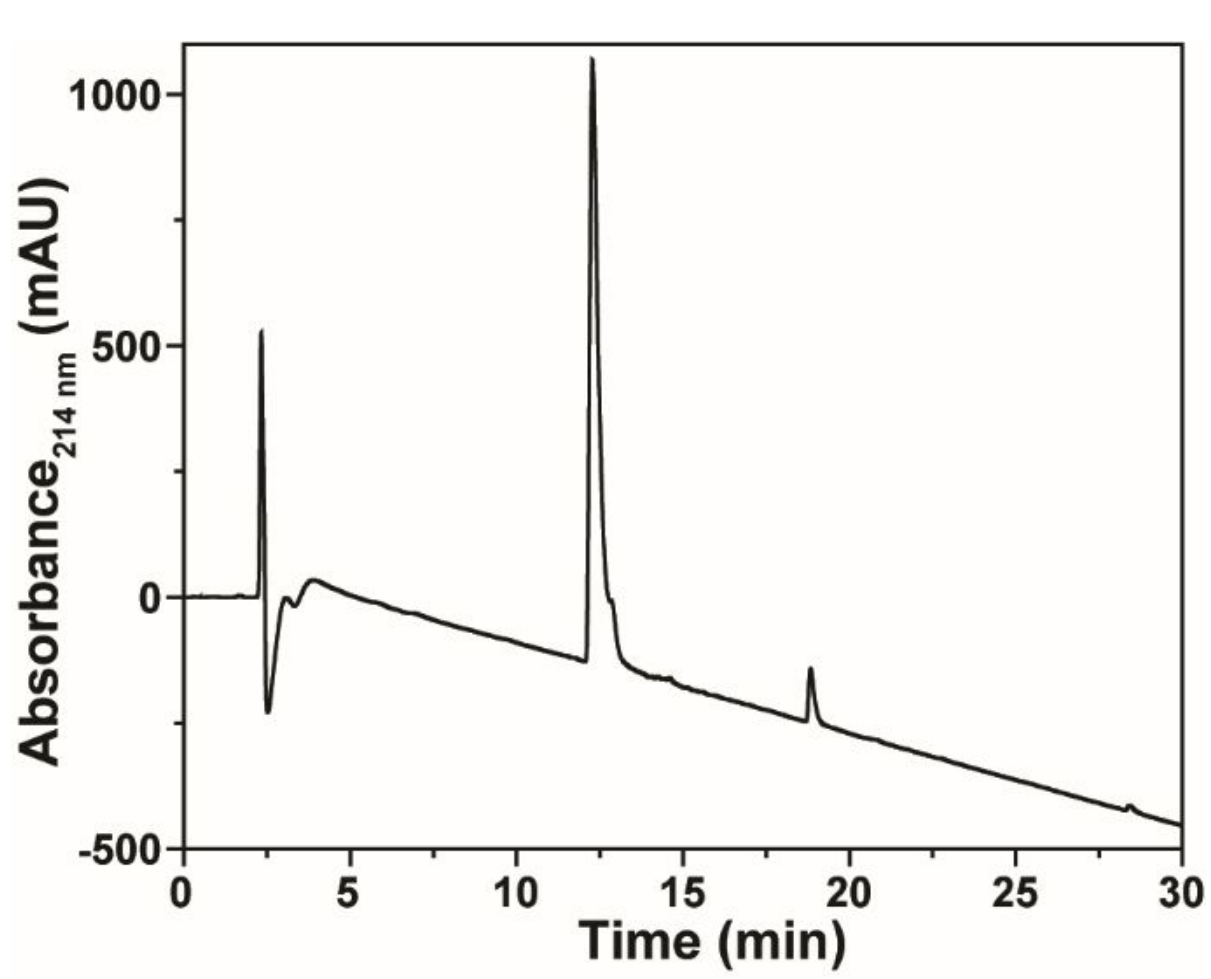

Supplementary Figure 31 Analytical HPLC result for BID.

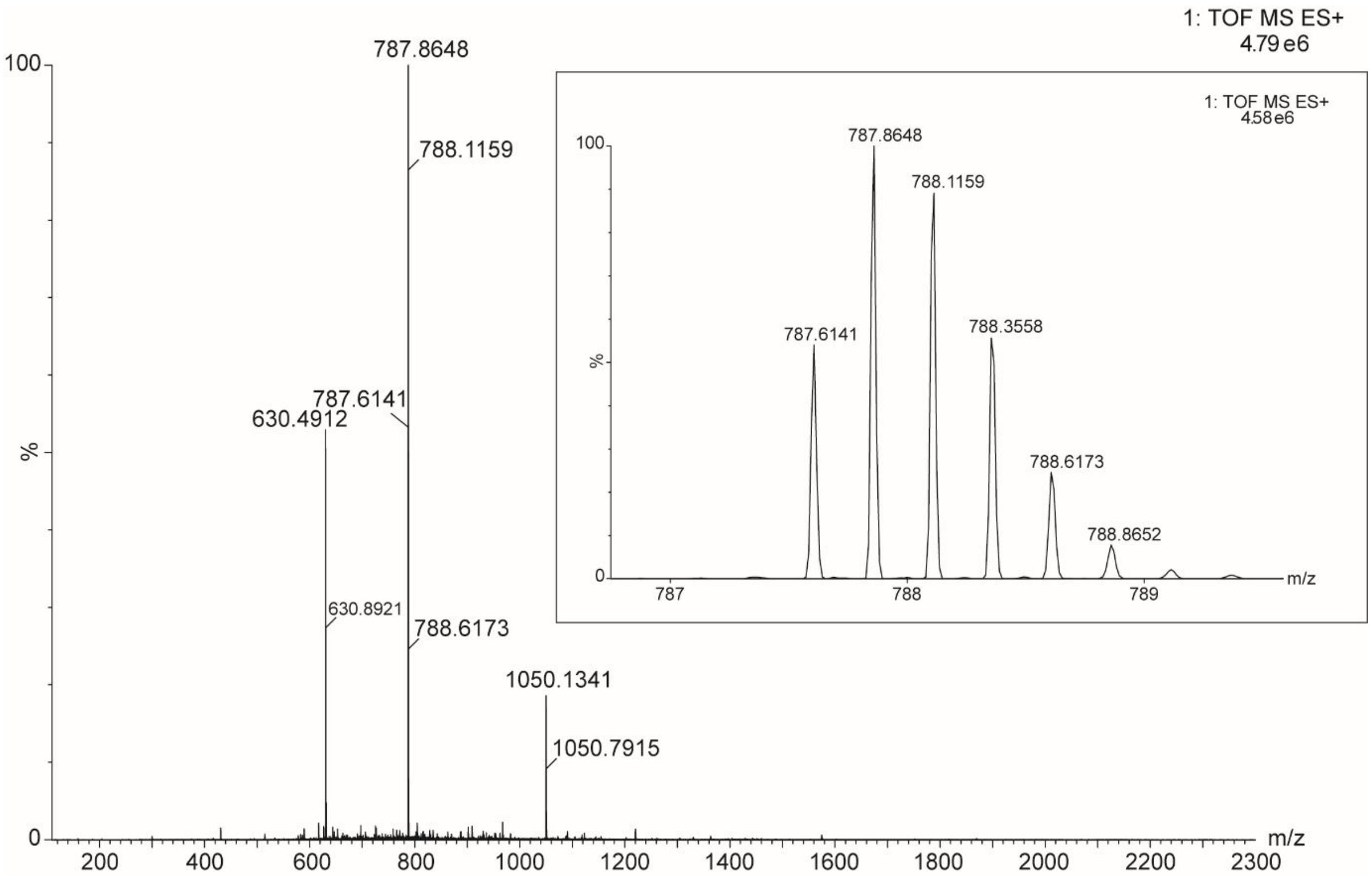

Supplementary Figure 32 Mass spectrometry result for FAM-BID Predicted 3,146.563. Observed 3,146.416.

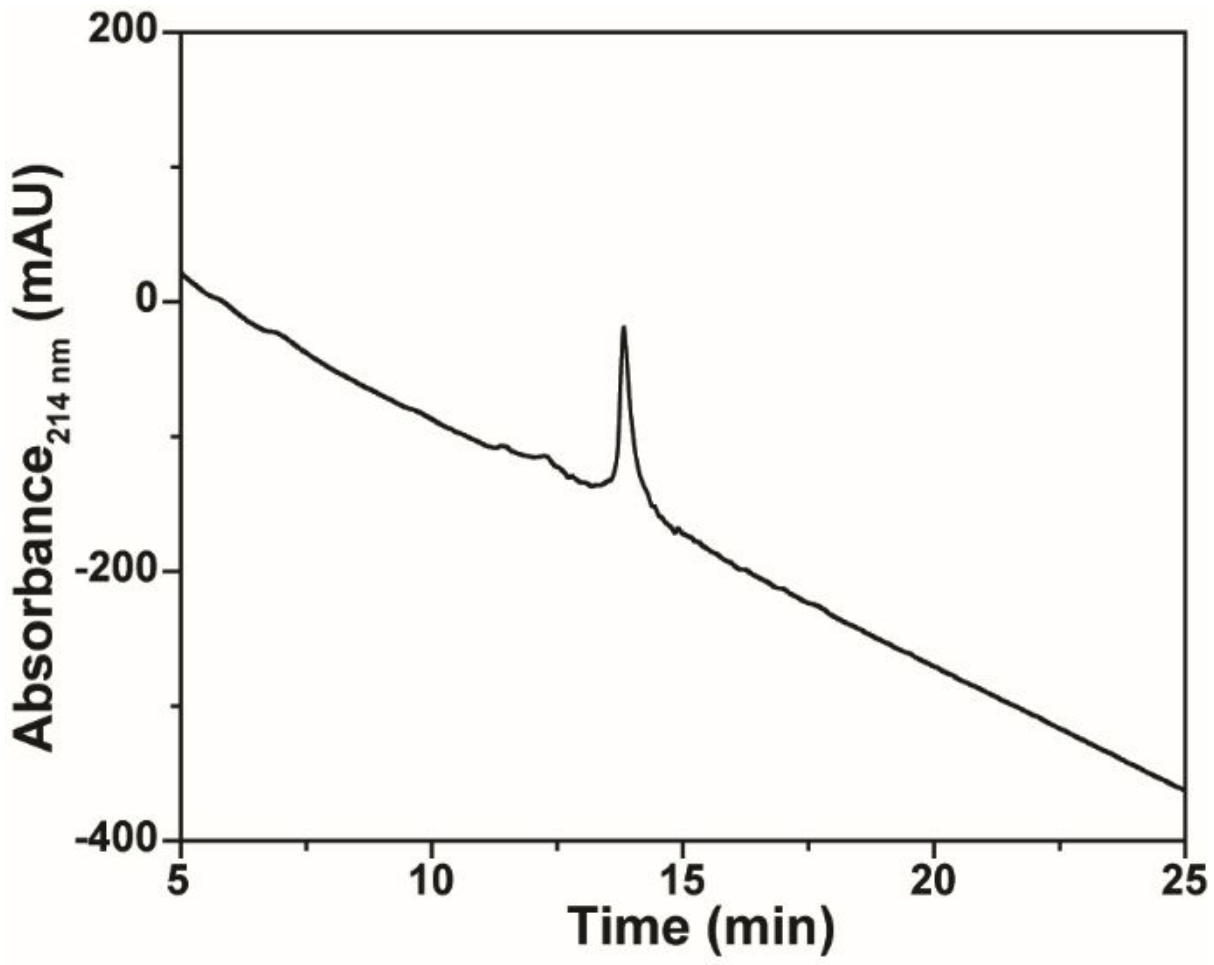

Supplementary Figure 33 Analytical HPLC result for FAM-BID

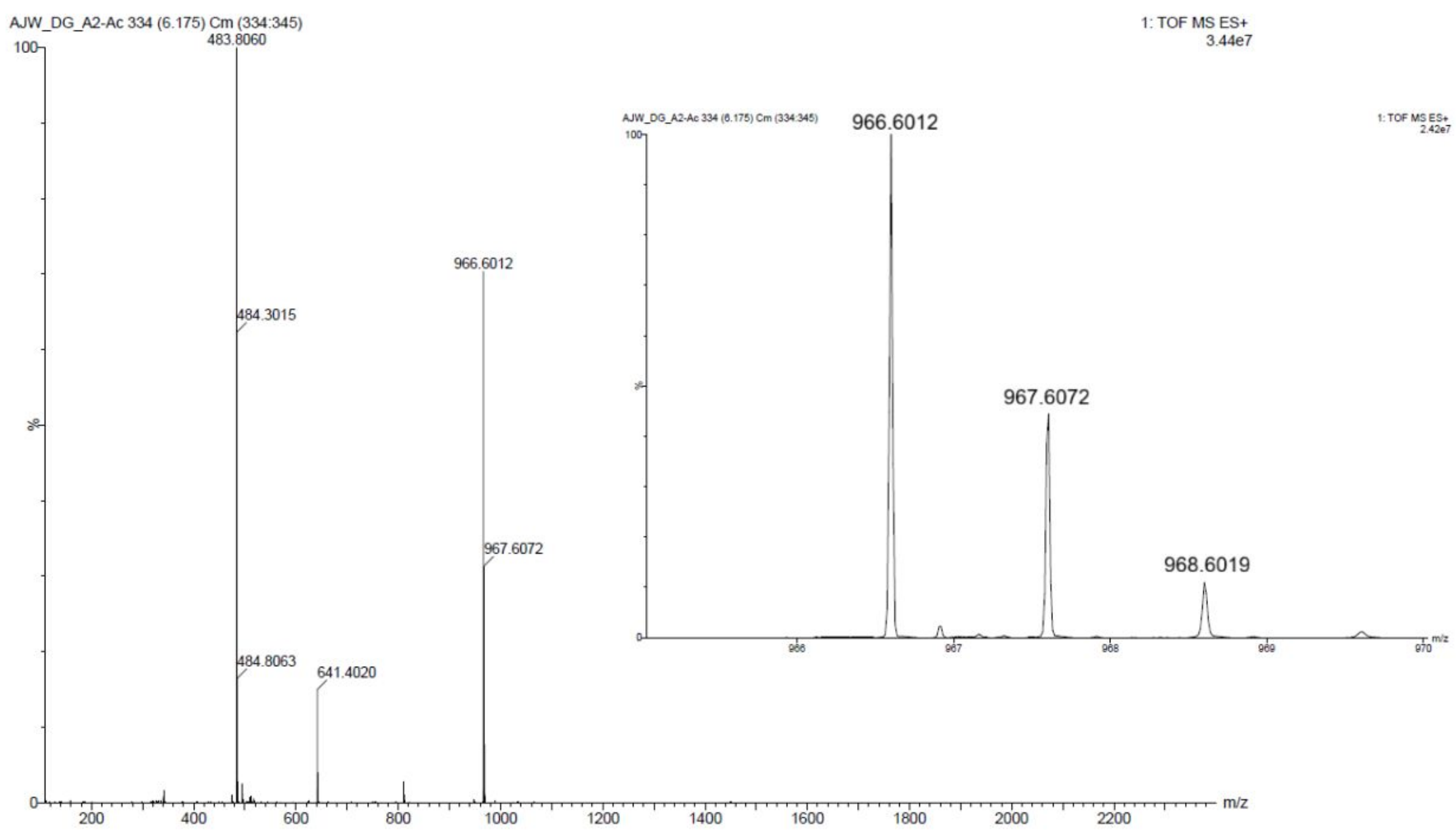

Supplementary Figure 34 Mass spectrometry result for DAPK1. Predicted 966.60 Observed 966.6

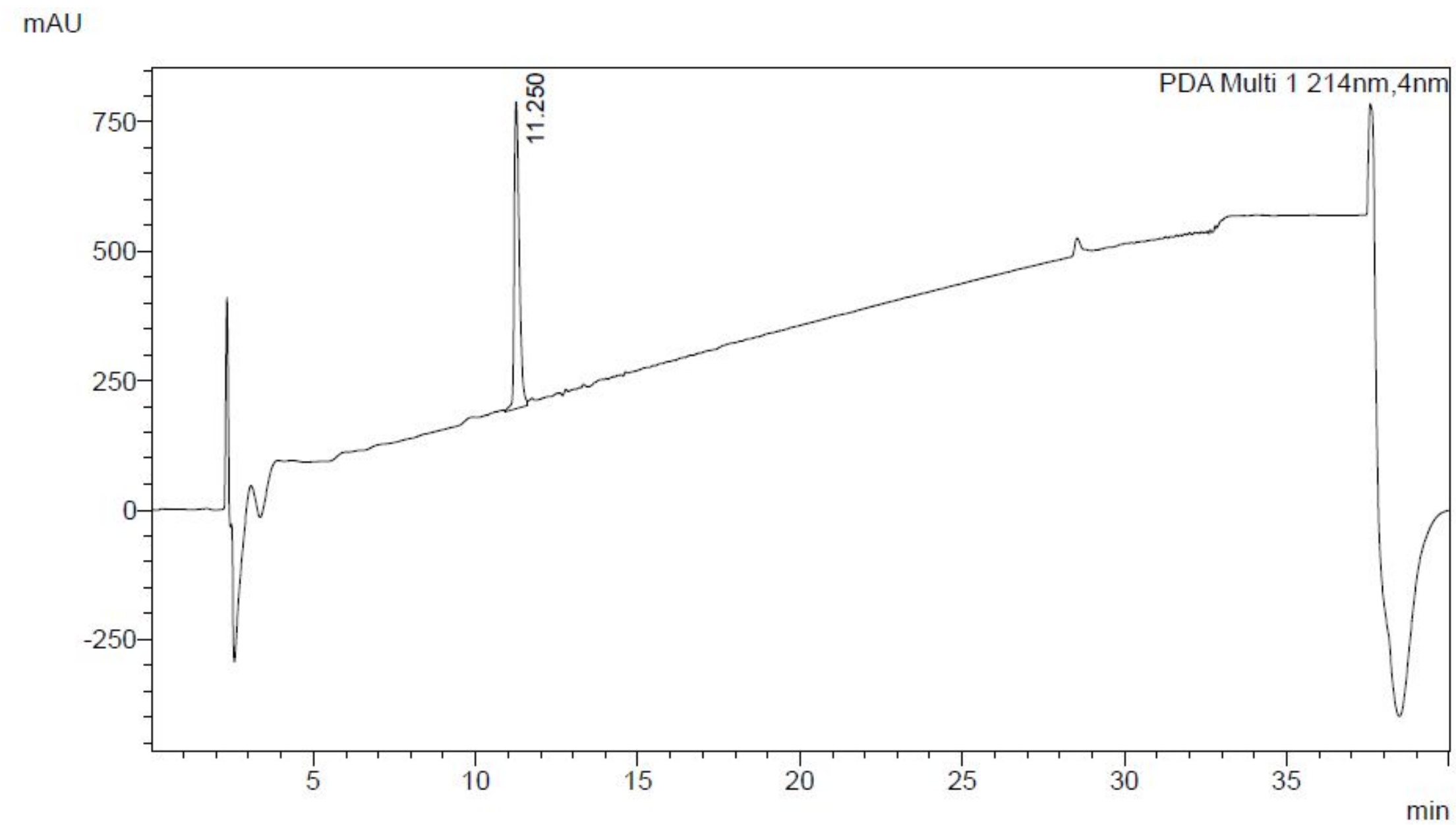

Supplementary Figure 35 Analytical HPLC result for DAPK1.

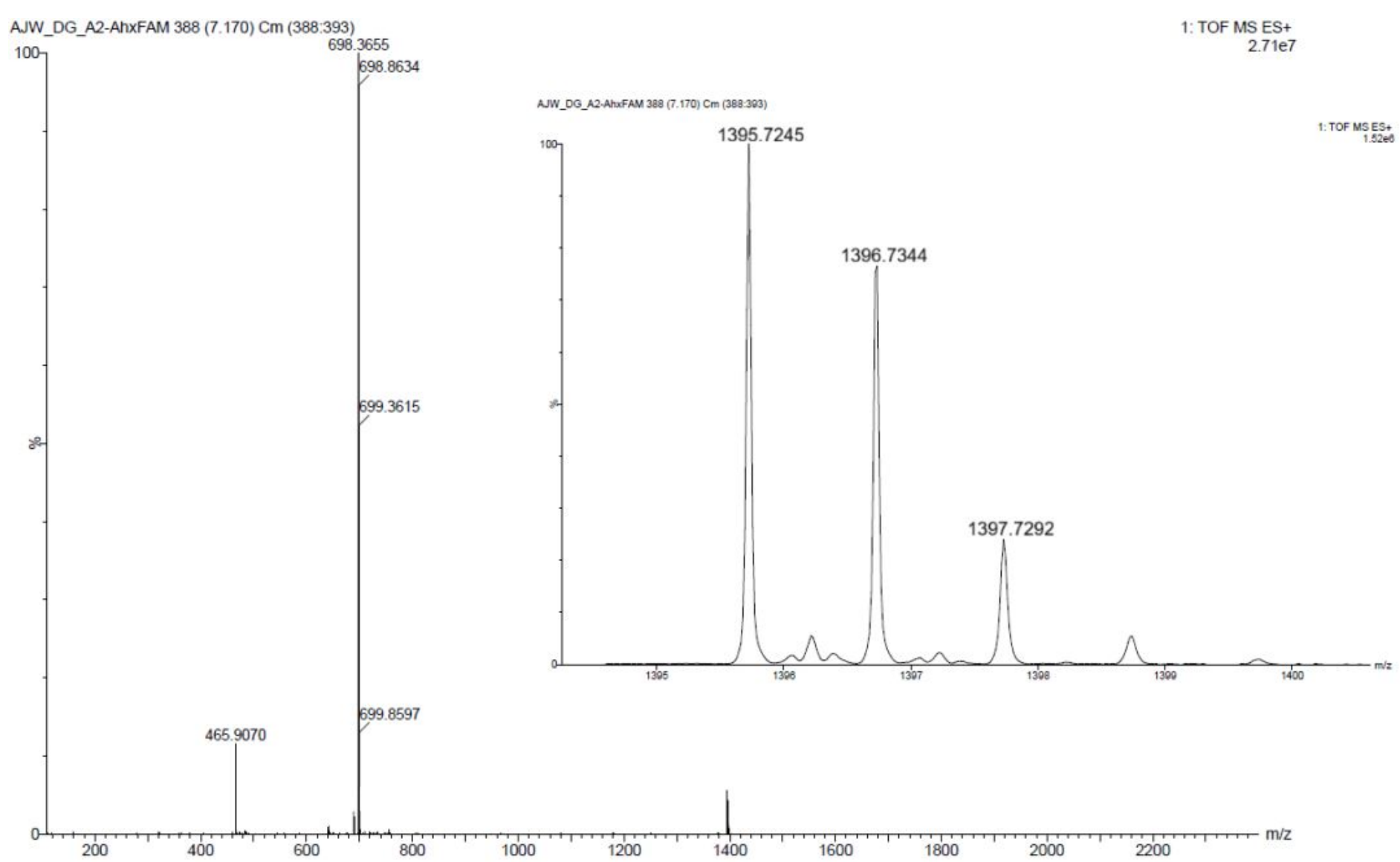

Supplementary Figure 36 Mass spectrometry result for FAM-DAPK1. Predicted 1395.72 Observed 1395.72

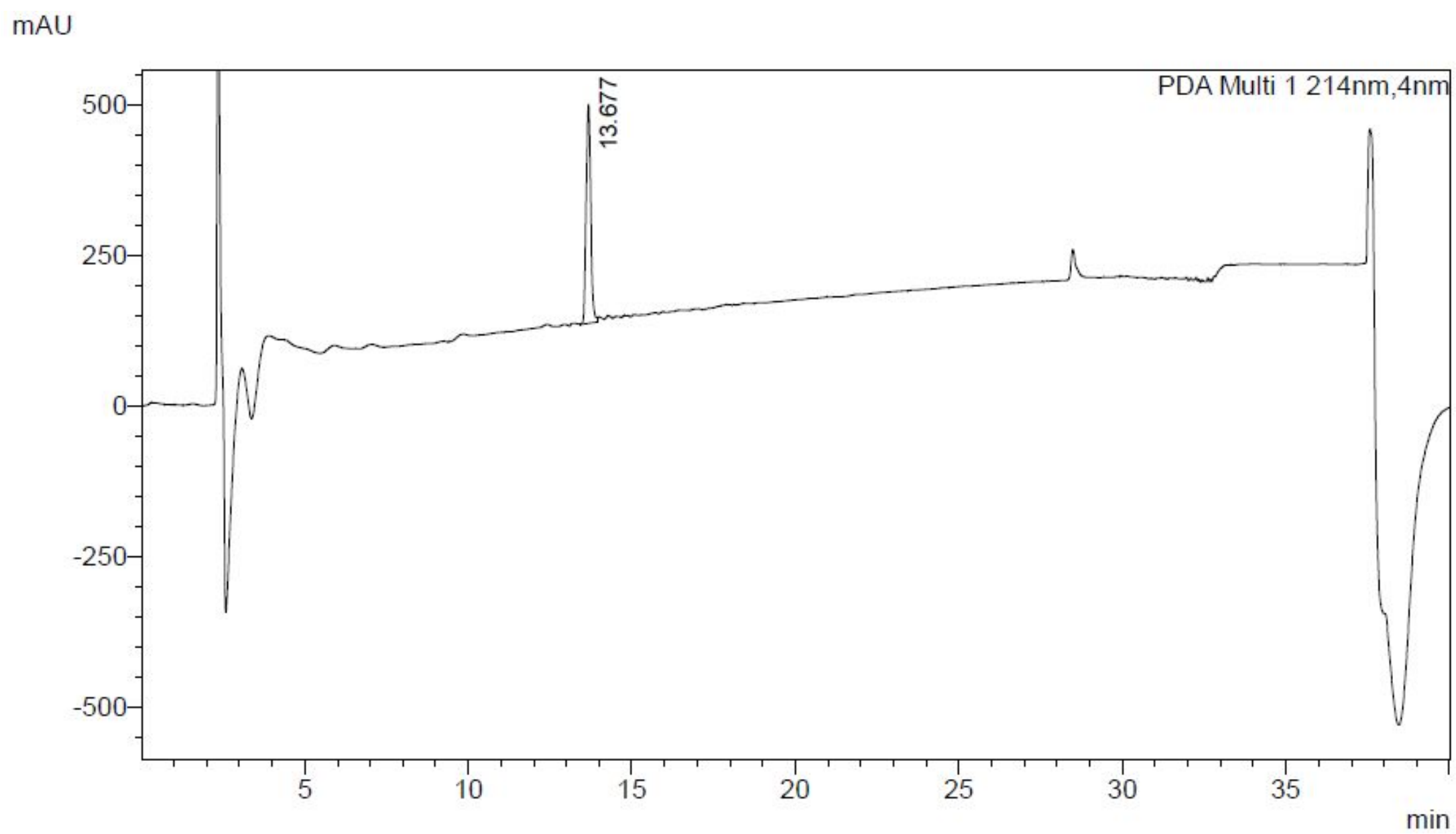

Supplementary Figure 37 Analytical HPLC result for FAM-DAPK1.

REFERENCES

1 Zhou, J. & Grigoryan, G. Rapid search for tertiary fragments reveals protein sequence–structure relationships. *Protein Sci.* **24**, 508-524 (2015). https://doi.org/https://doi.org/10.1002/pro.2610

2 Huang, P.-S. *et al.* RosettaRemodel: A Generalized Framework for Flexible Backbone Protein Design. *PLoS One* **6**, e24109 (2011). https://doi.org/10.1371/journal.pone.0024109

3 Rhys, G. G. *et al.* De novo designed peptides for cellular delivery and subcellular localisation. *Nat. Chem. Biol.* **18**, 999-1004 (2022). https://doi.org/10.1038/s41589-022-01076-6

4 Fletcher, J. M. *et al.* De novo coiled-coil peptides as scaffolds for disrupting protein–protein interactions. *Chem. Sci.* **9**, 7656-7665 (2018). https://doi.org/10.1039/C8SC02643B

5 Albanese, K. I. *et al.* Rationally seeded computational protein design of a-helical barrels. *Nat. Chem. Biol.* **20**, 991-999 (2024). https://doi.org/10.1038/s41589-024-01642-0

6 Acevedo-Jake, A. M. *et al.* Grafted Coiled-Coil Peptides as Multivalent Scaffolds for Protein Recognition. *ACS Chem. Biol.* **20**, 1309-1318 (2025). https://doi.org/10.1021/acschembio.5c00137

7 Zhao, H., Piszczek, G. & Schuck, P. SEDPHAT – A platform for global ITC analysis and global multi-method analysis of molecular interactions. *Methods* **76**, 137-148 (2015). https://doi.org/https://doi.org/10.1016/j.ymeth.2014.11.012

8 Scheuermann, T. H. & Brautigam, C. A. High-precision, automated integration of multiple isothermal titration calorimetric thermograms: New features of NITPIC. *Methods* **76**, 87-98 (2015). https://doi.org/https://doi.org/10.1016/j.ymeth.2014.11.024

9 Kabsch, W. XDS. *Acta Crystallogr. Sect. D.* **66**, 125-132 (2010). https://doi.org/doi:10.1107/S0907444909047337

10 Winter, G. *et al.* DIALS: implementation and evaluation of a new integration package. *Acta Crystallogr. Sect. D.* **74**, 85-97 (2018). https://doi.org/doi:10.1107/S2059798317017235

11 Evans, P. R. & Murshudov, G. N. How good are my data and what is the resolution? *Acta Crystallogr. Sect. D.* **69**, 1204-1214 (2013). https://doi.org/doi:10.1107/S0907444913000061

12 McCoy, A. J. *et al.* Phaser crystallographic software. *J. Appl. Crystallogr.* **40**, 658-674 (2007). https://doi.org/doi:10.1107/S0021889807021206

13 Adams, P. D. *et al.* PHENIX: building new software for automated crystallographic structure determination. *Acta Crystallogr. Sect. D.* **58**, 1948-1954 (2002). https://doi.org/doi:10.1107/S0907444902016657
